# Supplementary material for: Generalizable spelling using a speech neuroprosthesis in an individual with severe limb and vocal paralysis
Source: Nat Commun. 2022 Nov 8;13:6510. doi: 10.1038/s41467-022-33611-3 (PMC9643551; doi:10.1038/s41467-022-33611-3)
Supplement: Supplementary file 1 — Supplementary Information [file 41467_2022_33611_MOESM1_ESM.pdf]

Generalizable spelling using a speech neuroprosthesis in an individual with severe limb and vocal paralysis

Supplementary Information

Contents

List of investigators **3**

Supplementary notes **4**

Note S1. Assessment of the participant's articulatory inventory 4
Note S2. Participant survey on overt- versus silent-speech attempts 5
Note S3. Data re-normalization 7

Supplementary methods **8**

Method S1. Isolated-target task 8
Method S2. Speech-detection model 9
Method S3. Classification model 12
Method S4. Language modeling 18
Method S5. Adapted beam search 20

Supplementary figures **23**

Figure S1. Data collection timeline 23
Figure S2. Real-time signal-processing pipeline 24
Figure S3. Speech-detection model schematic 25
Figure S4. Effects of feature selection on code-word classification accuracy 26
Figure S5. Confusion matrix from isolated-target trial classification using HGA and LFS 27
Figure S6. Confusion matrix from isolated-target trial classification using only HGA 28
Figure S7. Confusion matrix from isolated-target trial classification using only LFS 29
Figure S8. Neural-activation statistics during overt- and silent-speech attempts . . 30

Supplementary tables **31**

Table S1. Copy-typing task sentences 31
Table S2. Statistical comparisons of character error rates across decoding-framework conditions 32
Table S3. Statistical comparisons of word error rates across decoding-framework conditions 33
Table S4. Statistical comparisons of classification accuracy across neural-feature types 34
Table S5. Statistical comparisons of the number of principal components required to explain more than 80% of the variance in the spatial dimension across neural-feature types 35
Table S6. Statistical comparisons of the number of principal components required to explain more than 80% of the variance in the temporal dimension across neural-feature types 36
Table S7. Statistical comparisons of classification accuracy across attempted-speech types with various training schemes 37
Table S8. Hyperparameter definitions and values 38

Supplementary references **39**

List of investigators

List of investigators (authors)

1. Sean L. Metzger*
2. Jessie R. Liu*
3. David A. Moses*
4. Kaylo T. Littlejohn
5. Maximilian E. Dougherty
6. Margaret P. Seaton
7. Josh Chartier
8. Gopala K. Anumanchipalli
9. Adelyn Tu-Chan
10. Karunesh Ganguly
11. Edward F. Chang

*These three authors contributed equally

Supplementary notes

Note S1. Assessment of the participant's articulatory inventory

The participant was diagnosed with anarthria by a certified speech-language pathologist. This section contains a summary of the results from an articulatory-inventory assessment with the participant conducted by this speech-language pathologist. The participant's articulation was characterized through a battery of tests.

An oral-mechanism test was used to assess gross movement of the jaw, tongue, and lips. The outcomes of this test indicated significant deficits in the articulatory performance of the participant. Residual jaw movement was relatively reliable, but all movements were very effortful and slow. The participant was also unable to produce lip rounding or puckering, and he could not maintain lip closure. Tongue performance was very poor and he could only produce a limited range of slow extension and elevation tongue movements.

A perceptual dysarthria assessment was used to measure the reliability of syllable-sequence production during overt-speech attempts. The participant was unable to produce multisyllabic sequences, with increasing breakdowns as the number of syllables per word increased and as the number of words per utterance increased. The participant's speech movements were characterized predominantly by spastic features, resulting in articulatory imprecision and slow rates of speech (about 1–2 syllables per breath).

A speech-articulation task was used to characterize performance on each phonetic consonant. The participant could only reliably produce nasals (/m/, /n/, /ng/) and a single palatal liquid (/y/). Other consonants included errors in voicing or were replaced with more reliable consonants; nasals would replace stops, and liquids would replace fricatives and affricates. Additionally, the participant's spasticity restricts the range and speed of articulatory motions, contributing to poor control of airflow and phonation.

Note S2. Participant survey on overt- versus silent-speech attempts

We asked the participant the following questions about controlling the spelling system using either silent or overt attempts to speak. The participant's responses are provided after each question.

1. How long do you think you could comfortably use the spelling system for communication with overt-speech attempts? **Response: 15 minutes**

2. How long do you think you could comfortably use the spelling system for communication with silent-speech attempts? **Response: 30 minutes**

3. Can you please rank your comfort using the spelling system with overt-speech attempts on a scale from 1–10?
   Response: 5

4. Can you please rank your comfort using the spelling system with silent-speech attempts on a scale from 1–10?
   Response: 8

5. What is the minimum amount of time you need between go cues to use the spelling system with overt-speech attempts?
   Response: 4 seconds

6. What is the minimum amount of time you need between go cues to use the spelling system with silent-speech attempts?
   Response: 2.5 seconds

7. How does using silent-speech attempts compare to using overt speech attempts to control the speller device?

   (a) Silent is much easier than overt
   (b) Silent is easier than overt
   (c) Silent is the same as overt
   (d) Silent is harder than overt
   (e) Silent is much harder than overt

   Response: (a) Silent is much easier than overt

The participant's responses are summarized below. Overall, the participant vastly prefers silent-speech attempts to control the spelling neuroprosthesis.

Question	Overt	Silent
How long could you comfortably use the device?	15 minutes	**30 minutes**
What is your comfort using the device for communication (1-10)?	5	**8**
What is the smallest amount of time you need between go cues?	4 seconds	**2.5 seconds**
How much easier is using silent speech attempts than overt?	(n/a)	**Much easier**

Note S3. Data re-normalization

To promote neural-feature consistency across recording sessions, we used a running 30-second z-score on all neural features (see Figure S2). However, the neural activity recorded during the participant's attempts to squeeze his right hand typically differed in signal magnitude when compared to activity recorded during silent-speech attempts. As a result, when using a running z-score, some isolated-target task blocks with only speech content (letter and NATO code-word trials) or only attempted hand-movement trials had different neural-feature baselines than isolated-target blocks with both speech and hand-movement trials.

To mitigate this, we jointly re-normalized letter and NATO code-word isolated-target blocks and attempted hand-movement isolated-target blocks that were recorded on the same day. For each recording day, and independently for each speech type (silent or overt), we combined all attempted speech trials and attempted hand-movement trials that were recorded on that day by concatenating (along the time dimension) time windows of neural features (high-gamma activity and low-frequency signals without z-score normalization) associated with these trials. These time windows of neural features ranged from 2 seconds before to 3.5 seconds after the go cue for each trial. To reduce the effect of potential signal artifacts in these un-normalized signals, we clipped the signal magnitude for each feature (each electrode channel for each feature type) to be within the 1st and 99th percentiles of the signal magnitudes recorded for that feature. Then, we re-normalized the neural features for each trial recorded on that day by subtracting the feature-wise mean and dividing by the feature-wise standard deviation of the concatenated data matrix. Note that some task blocks containing only attempted speech or only attempted hand-movements were not re-normalized in this manner (if there were not both types of data recorded on the same day). Additionally, because some attempted hand-movement blocks were recorded on days where both overtly and silently attempted NATO code-word isolated-target were also recorded, this meant that there were three possible types of attempted hand-movement blocks: blocks that were not re-normalized (these blocks were not recorded on the same day as blocks containing only attempted speech), blocks that were re-normalized with blocks that only contained overt-speech attempts, and blocks that were re-normalized with blocks that only contained silent-speech attempts. Data from task blocks that were not re-normalized used the running 30-second z-score normalization procedure and automatic artifact rejection described in Figure S2.

Supplementary methods

Method S1. Isolated-target task

We recorded the participant's neural activity as he silently (or sometimes overtly) attempted to say prompted utterances or perform prompted motor movements during an isolated-target task. As described in the Methods section of the main text, each trial of the isolated-target task began with the textual presentation of a single speech or motor target on the participant's screen with 4 dots on either side of the text. These dots disappeared one at a time (simultaneously on each side of the text) at a constant rate, providing task timing to the participant. As the final dot disappeared, the text target turned green, representing a go cue. At this go cue, the participant was instructed to attempt to produce the target. The text target remained on the participant's screen for a brief interval before the screen was cleared and the next trial began.

We collected the following four utterance sets with the isolated-target paradigm for training the speech detection and neural classification models:

1. 26 English letters
2. 26 NATO code words
3. 26 NATO code words and attempted hand squeeze
4. Attempted hand squeeze and 3 other attempted motor movements

Within each block of the isolated-target task, the rate at which the countdown dots disappeared τ_{p} and the duration that the target text remained on the screen after the go cue τ_{t} was identical across trials within a single block. However, these two task-interval parameters did vary across blocks. For the attempted motor movement blocks, we used $\tau_{\text{p}} \in [0.35, 0.5]$ seconds per dot and $\tau_{\text{t}} = 4.0$ seconds. For all other isolated-target blocks, we used $\tau_{\text{p}} \in [0.45, 1.5]$ seconds per dot and $\tau_{\text{t}} \in [0.45, 6.0]$ seconds.

Method S2. Speech-detection model

We designed a speech-detection model to analyze the neural features in real time to identify when a silently attempted speech event occurred. We used this speech detector to enable volitional engagement of the spelling system during real-time sentence spelling. All data used to train and evaluate the speech detector was either trials of attempted hand squeezes or of silently attempted speech (no overtly attempted speech data was used).

Data preparation

We trained the speech detector using data from isolated-target task blocks containing trials of the 26 NATO code words, blocks containing trials of the 26 NATO code words and the attempted right-hand squeeze, and blocks containing a variety of attempted motor movements including the attempted hand squeeze (from which we only used the attempted hand squeeze). We used four categories to label each time point of neural-feature data to train the speech detector: "speech preparation", "speech", "motor", and "rest". Time points between the appearance of a target NATO code word on the participant's screen and the associated go cue were labeled as speech preparation. Time points between a go cue and 1 second after that go cue for NATO code-word attempts were labeled as speech. Time points between a go cue and 2 seconds after that go cue for attempted hand squeezes were labeled as motor. Time points between the end of the allotted time period for an attempt (1 second after the go cue for speech or 2 seconds for hand-squeezes) and the end of that trial (when the screen cleared for an inter-trial interval) were not trained on. Training data for the speech detector included blocks of the attempted motor isolated target task. For blocks containing only attempted motor movements, time points during attempted motor trials that were not the attempted hand squeeze were ignored. All other time points were labeled as rest.

The speech detector used both low-frequency signals (LFS) and high-gamma activity (HGA) as features at 200 Hz. Note that this is different than the classifier, which also used these features but further downsampled them to 33.3 Hz.

Model architecture and training

We used `Python 3.6.6` and `PyTorch 1.6.0` to create and train the speech detector [1]. The speech detector contained a stack of 3 long short-term memory (LSTM) layers with 100, 50, and 50 nodes, respectively. The LSTM layers were followed by a single fully-connected layer that projected the latent dimensions to probabilities across the four classes (speech preparation, speech, rest, and motor). The model processed each time point continuously from the feature stream, outputting a continuous stream of probabilities (one predicted probability vector per neural-feature time point at 200 Hz). A schematic of the model is shown in Figure S3.

The speech-detection model was trained to minimize a modified cross-entropy loss. Cross-entropy loss is originally defined as:

$$\begin{aligned} \mathrm{H}_{P,Q}\left(\boldsymbol{\ell} \mid \mathbf{y}\right) &= \mathbb{E}_P\left[-\log Q\left(\boldsymbol{\ell} \mid \mathbf{y}\right)\right] \\ &\approx -\frac{1}{N}\sum_{n=1}^{N} \log Q\left(\ell_n \mid y_n\right), \end{aligned} \tag{S1}$$

where:

- P : The true distribution of the classes, determined by the assigned class labels $\boldsymbol{\ell}$.
- N : The number of samples.
- $\mathrm{H}_{P,Q}(\boldsymbol{\ell} \mid \mathbf{y})$: The cross entropy of the predicted distribution with respect to the true distribution for $\boldsymbol{\ell}$.
- $\log$: The natural logarithm.

We modified this loss to add an extra penalty on 3 types of incorrect predictions: time points that were labeled as motor but predicted to be speech, time points that were labeled as speech but predicted to be motor, and time points that were labeled as rest but predicted to be speech. In practice, we defined w_n as 1.1. With these modifications, the cross-entropy loss defined in Equation S1 is redefined as:

$$\mathrm{H}_{P,Q}(\boldsymbol{\ell} \mid \mathbf{y}) \approx -\frac{1}{N}\sum_{n}^{N} w_n \log Q(\ell_n \mid y_n), \tag{S2}$$

where w_n is the penalty weight for sample n and is defined as:

$$w_n := \begin{cases} 1.1 & \text{if } (\ell_n = \text{motor}) \text{ and } \left(\arg\max_{l \in L}\left[Q(l \mid y_n)\right] = \text{speech}\right) \\ 1.1 & \text{if } (\ell_n = \text{speech}) \text{ and } \left(\arg\max_{l \in L}\left[Q(l \mid y_n)\right] = \text{motor}\right) \\ 1.1 & \text{if } (\ell_n = \text{rest}) \text{ and } \left(\arg\max_{l \in L}\left[Q(l \mid y_n)\right] = \text{speech}\right) \\ 1 & \text{otherwise.} \end{cases} \tag{S3}$$

We used this penalty modification to reduce the likelihood that the speech detector would make false-positive mistakes (such as erroneously detecting an attempted-speech event when the participant was actually attempting to squeeze his hand).

As previously described in [2], we used truncated backpropagation through time (BPTT) to train the speech detector. In brief, we manually implemented BPTT by only letting the speech-detection model backpropagate 500 ms at a time to prevent the model from relying on task periodicity to make predictions. We used the Adam optimizer to minimize the cross-entropy loss given in Equation S2 [3], with a learning rate of 0.001 and default values for the remaining optimization parameters. To prevent overfitting, we used early stopping on a held-out validation set and a dropout of 0.5 on each LSTM layer except for the final layer. For all training steps, we balanced classes between (included the same number of training examples for) the 4 possible classes.

Event detection

During real-time sentence spelling, the speech detector continuously processed time points of LFS and HGA and yielded a stream of silent-speech probabilities. We identified silent-speech events from this stream of probabilities using the same approach described in Supplementary

Section S8 of [2]. In brief, the speech probabilities were first temporally smoothed using a moving window average. Then, we binarized the smoothed probabilities using a probability threshold. Finally, we "de-bounced" these binarized values by requiring that a change in binary state (from absence of speech to presence of speech, or vice versa) must last for longer than a certain duration of time before the change is deemed a speech onset or offset. These 3 parameter values were chosen via hyperparameter optimization and are listed in Table S8.

Hyperparameter optimization

The hyperparameter optimization process is identical to our previous work [2]. In brief, we used the `hyperopt` Python package [4] to optimize the 3 detection hyperparameters by minimizing a cost function based on a detection score. As defined in Supplementary Section S8 of [2]), the detection score is a measure encompassing both how accurately individual time points were predicted as speech or non-speech and how accurately the detector identified attempted-speech events in general. The cost function used to optimize the hyperparameters seeks to maximize the detection score while minimizing the time-threshold parameter (because we wanted to minimize the amount of time required to detect a silent-speech attempt. The cost function was defined as:

$$c_{\mathrm{hp}}(\Theta) = (1 - s_{\mathrm{detection}})^2 + \lambda_{\mathrm{time}}\,\theta_{\mathrm{time}}, \tag{S4}$$

where:

- $c_{\mathrm{hp}}(\Theta)$: The value of the objective function using the hyperparameter value combination Θ.
- λ_{time} : The penalty applied to the time-threshold duration.
- θ_{time} : The time-threshold duration value, which is one of the three parameters contained in Θ.

Here, we used $\lambda_{\mathrm{time}} = 0.00025$.

Because we only optimized the detection parameters that were applied to speech probabilities, we were able to compute the speech probability across a set of task blocks from a trained model and use the speech probabilities from these blocks to evaluate the hyperparameter combinations. After training a model on isolated-target blocks, we used the model to predict the speech probabilities for 12 held-out blocks of the isolated-target task containing NATO code-word silent-speech attempts and attempted hand squeezes. We chose to optimize over blocks containing both the silent-speech attempts and attempted hand squeezes because the real-time sentence-spelling task involved both of these types of attempts. After 1000 optimization iterations, we selected the final hyperparameters from the optimization run with the lowest cost value.

Method S3. Classification model

Data preparation

We trained the classifier using data from isolated-target task blocks containing trials of the 26 NATO code words, blocks containing trials of the 26 NATO code words and the attempted right-hand squeeze, and blocks containing a variety of attempted motor movements including the attempted hand squeeze (from which we only used the attempted hand squeeze). For the classifiers used during the feature-type, speech-type, and utterance-set comparisons, only data from isolated-target task blocks were used.

During training of the classifiers for real-time sentence spelling (and associated offline analyses), we also included sentence-spelling (copy-typing) trials in which the decoded sentence had a 0.0 character error rate (CER). These sentence-spelling trials constituted 3.06% of the data for overt-speech attempts (preliminary sentence-spelling trials with overt-speech attempts were collected but not used during evaluation) and 22.7% of the data for silent-speech attempts. For these classifiers, we also used a transfer-learning approach to pre-train on overt-speech attempts and then fine-tune on silent-speech attempts (except where otherwise noted; more details are provided later in this section). We never included sentence-spelling trials during classifier training that were recorded during the same session as (or, for associated offline analyses, a proceeding session of) any trials that were used during testing; classifiers were not recalibrated or updated during an evaluation session. The usages of certain datasets for certain evaluations are described below:

- For real-time sentence-spelling performance evaluation and related offline analyses, including the beam-search, language-model, vocabulary-set, and task-condition assessments (see Figures 2 and 6 in the main text), models were:
    1. Pre-trained using data collected during overtly attempted isolated-target and sentence-spelling tasks.
    2. Fine-tuned using data collected during silently attempted isolated-target and sentence-spelling tasks.
    3. Tested using held-out data collected during silently attempted sentence-spelling tasks.
- For offline feature-type (HGA versus LFS) comparisons (see Figure 3 in the main text), models were trained and tested using data collected during silently attempted isolated-target tasks.
- For offline utterance-set (letters versus code words) comparisons (see Figure 4 in the main text), models were trained and tested using silently attempted isolated-target data.
- For offline speech-type (silent versus overt) comparisons (see Figure 5 in the main text), models were trained and tested using overtly and silently attempted isolated-target data, including pre-training and fine-tuning steps where applicable.

There was no overlap between data used for evaluation and data used for hyperparameter optimization.

For each isolated-target trial, we defined the relevant time window of neural features (high-gamma activity (HGA) and low-frequency signal (LFS) features at 200 Hz) as 2 seconds before the go cue to 4 seconds after. This window of neural features was larger than the windows actually used for training and testing (detailed below in the "Architecture and training" sub-section) because we employed a time-jittering data augmentation, where smaller windows are pulled from this larger trial-relevant window. We then decimated the neural activity by a factor of 6 to 33.33 Hz with a 16.67 Hz anti-aliasing filter applied prior to decimation. We normalized each time sample to have an ℓ_2-norm of 1 across all neural features (each electrode channel and separately for the HGA and LFS feature types). For real-time inference and for offline evaluations, we used the combined (concatenated) HGA+LFS features during relevant time windows of neural activity. Thus, for each training example, we had a matrix of neural activity x_i of shape (T, C), where T is the number of time steps and C refers to the 256 features (2 features from each of the 128 electrodes). If only one feature stream was being used for a particular analysis, C would be equal to 128.

Modeling

Architecture and training

To model the temporal and spatial dynamics of the participant's neural activity during silent-speech attempts, we trained artificial neural networks to classify which NATO code word (or the imagined hand squeeze) the participant had produced given a 2.5-second window of neural features after the associated go cue. We used gated-recurrent unit (GRU) layers [5], which have been shown to outperform other recurrent architectures (such as long-short term memory networks) [6] on sequence tasks [7].

In the classifier, neural features were first processed by a 1-dimensional convolutional layer parameterized by weights W and bias term b. This results in an output representation h_n (the output of hidden layer n) defined as:

$$h_{1,j} = b_j + \sum_{k=0}^{C-1} W[j,k] * x_i[:,k], \tag{S5}$$

where $h_{1,j}$ is element j of the output of hidden layer 1, $*$ denotes the valid cross-correlation operator, and C refers to the number of neural features in the input matrix x_i.

This representation was then passed into a stack of n GRU layers. Each unit was parameterized by W_i, b_i, W_h, and b_h, which are weights and biases that acted on the input and hidden states, respectively. Portions of each matrix were dedicated to a reset gate r_t, an update gate z_t, and a new gate n_t.

At each time point t, the GRU computed:

$$\begin{aligned}
r_t &= \sigma(W_{ir}x_t + b_{ir} + W_{hr}h_{(t-1)} + b_{hr}),\\
z_t &= \sigma(W_{iz}x_t + b_{iz} + W_{hz}h_{(t-1)} + b_{hz}),\\
n_t &= \tanh(W_{in}x_t + b_{in} + r_t * (W_{hn}h_{(t-1)} + b_{hn})),\\
h_t &= (1 - z_t) * n_t + z_t * h_{(t-1)},
\end{aligned}$$

where $*$ denotes the Hadamard product, σ denotes the sigmoid function, and h_t is the output at each time point t for this layer. Basically, the GRU decided at each time point how much to update the hidden state from its previous value given the new activity (with the reset function incorporated) using z_t. Each layer's output h_n is used as the input to the next layer. During training, to minimize overfitting, we used dropout [8] to randomly set elements of h_n to 0.0 with probability p_{dropout}, which we determined through hyperparameter optimization.

To improve accuracy, we used bidirectional GRU layers. This means that at each GRU, the input was copied, flipped backwards, and then used as an input to the network. This enabled us to learn forward and backward representations and use them as context when predicting class probabilities.

To compute a predicted probability distribution over the 26 NATO code words and imagined hand squeeze given the final time point of the final GRU layer, we multiplied this by a matrix W_{out} and add a bias term b_{out}, where W_{out} has shape $(N_{h_n}, 27)$, with N_{h_n} corresponding to the number of hidden units in the final GRU layer. We then applied a softmax function to these activations, giving the value of the output vector $\hat{y}$ for each window i and each element (class) k to be:

$$\hat{y}_{i,k} = \frac{\exp((W_{\text{out}} h_n)_k)}{\sum_j (\exp((W_{\text{out}} h_n)_j)}, \tag{S6}$$

where $\hat{y}_i$ can be thought of as a multinomial distribution over the possible output classes given sample x_i and the parameters of our neural-network model θ.

The goal during training was to maximize the likelihood of our labeled training data given the neural activity and θ, which can be written as the optimization problem:

$$\begin{aligned} \theta^* &= \arg\max_\theta \prod_i p_\theta(y_i \mid x_i) \\ &= \arg\max_\theta \sum_i log(p_\theta(y_i \mid x_i)). \end{aligned} \tag{S7}$$

We approximated the solution to this problem using mini-batch stochastic gradient descent to solve the equivalent optimization problem:

$$\theta^* = \arg\min_\theta \sum_i -log(p_\theta(y_i \mid x_i)). \tag{S8}$$

Specifically, we use the Adam optimizer [9], which incorporates adaptive estimates of the mean and un-centered variance of the gradient to improve rates of convergence. We implemented the neural-network models and optimization procedures using `PyTorch 1.6.0` [10]. We early stopped models after 5 epochs with no improvement in validation set accuracy and used the model parameters corresponding to the highest validation-set accuracy.

For real-time inference, we ensembled models by averaging 10 model predictions to improve performance, as in [2].

We used models that were trained using a 2.69-second window of neural features then tested using 2.5-second windows. This discrepancy was caused by a change made to task timing before collection of the sentence-spelling evaluation blocks; specifically, we had originally

planned to use 2.69-second letter-decoding cycles during sentence spelling and trained the classifiers accordingly, but ultimately we decided to use 2.5-second letter-decoding cycles for a faster pacing. Because the classifier was designed to perform inference on inputs with flexible window lengths, we were able to evaluate the 2.5-second windows seamlessly and without any noticeable performance degradation.

Augmentations

To bolster classifier performance, we used data augmentations, which have been shown to improve generalization and reduce overfitting for both images [11, 12] and neural activity [2, 13]. The following augmentations were applied sequentially to each trial of neural activity x_i during training (but not testing), without changing the associated label y_i:

1. Time jittering: shift the neural features by a time shift τ, such that:

$$x_i(t) = x_i(t - \tau),$$

$$\tau \sim \mathcal{U}(-j, j),$$

   where j is a hyperparameter.

2. Temporal masking: set some time points of the neural features to 0, such that:

$$x_i[t_0 : t_1] = (1 - \delta_p),$$

$$t_1 = t_0 + s$$

   ,

$$s \sim \mathcal{U}(0, b),$$

   where t_0 is a randomly drawn time point within x_i and p is the probability of δ_p being one, and the time points being set to 0. Both b and p are hyperparameters.

3. Scaling: scale the magnitude of the neural features, such that:

$$x_i = \alpha x_i,$$

$$\alpha \sim \mathcal{U}[\alpha_{min}, \alpha_{max}],$$

   where α_{min} and α_{max} are hyperparameters.

4. Additive noise: add a matrix of random Gaussian noise to the neural features x_i, such that:

$$x_i = x_i + \mathcal{N}(0, \sigma_n^2),$$

   where σ_n is a hyperparameter.

5. Channel-wise noise: offset the neural features by a value randomly sampled from a Gaussian distribution to each channel c, such that:

$$x_i[:, c] = x_i + \mathcal{N}(0, \sigma_{ch}^2),$$

   where σ_{ch} is a hyperparameter and is shared across all features.

Model pre-training and fine-tuning

When training the ensemble of classifiers used for real-time sentence spelling, which were also subsequently used during offline analyses to evaluate the effect of the beam search, the language model, and different vocabulary sizes on the real-time copy-typing results, we first pre-trained models on overt-speech attempts and then fine-tuned them on silent-speech attempts. Specifically, we trained classifiers on an initial dataset containing overt-speech attempts with a learning rate of 10^{-3}. We split this initial dataset into training and validation sets, and we early stopped models after the accuracy on the validation set did not improve for 5 epochs in a row and reset the model parameters to those corresponding to the highest validation accuracy. Then, starting from those parameters, we fine-tuned the model on a second dataset containing silent-speech attempts, which involved training the pre-trained model on the new dataset with the same early-stopping process but with a smaller learning rate of 10^{-4}.

Hyperparameter optimization

For the classifiers, we optimized the number of layers, number of hidden nodes in each layer, kernel size, stride, dropout rate, and augmentation hyperparameters using the Asynchronous Hyperband (ASH) method [14] with the `Ray` software package. We used the `Hyperopt` software package to suggest the next set of hyperparameters after each evaluation run [15]. The search space and final values are detailed in S8, and we searched 300 possible sets of hyperparameters.

We used all of the neural data from the overtly and silently attempted trials from isolated-target blocks recorded before collecting any sentence-spelling task blocks as the held-out validation dataset during hyperparameter optimization. We used the remaining isolated-target trials as training data during this process. During each evaluation run in the hyperparameter search, we initialized a new model using a set of hyperparameters determined by the algorithm and then began training the model. Because we performed model pre-training before fine-tuning, we first trained and evaluated the model on data recorded during overt-speech attempts. After each epoch of training, we evaluated the model accuracy on these overtly attempted trials with the current hyperparameter set. Because ASH uses the accuracy at each step to terminate underperforming hyperparameter combinations early, we scaled the accuracy by 0.1 during this pre-training process to prevent it from terminating prematurely if accuracy decreased once fine-tuning began.

We early stopped models as usual, re-instating the parameters corresponding to the highest accuracy. Then, starting from those parameters, we fine-tuned (and evaluated) the model on the silently attempted portion of the dataset with a learning rate of 10^{-3}. Here, we purposefully used a greater learning rate than what was used during the final training procedure (which was 10^{-4}) to evaluate hyperparameter combinations more quickly. ASH monitored the un-scaled accuracy values during the fine-tuning process. We terminated hyperparameter-optimization iterations after the accuracy on the hyperparameter-optimization dataset did not improve for 5 epochs in a row, and we kept the best accuracy as the score for that set of hyperparameters.

We used the resulting optimal neural-classifier hyperparameters for all of the real-time sentence-spelling blocks and analyses, and the blocks used for hyperparameter optimization

were excluded from being used as evaluation blocks in all analyses.

Before each real-time sentence-spelling evaluation session, we trained 10 neural classifier models on all the data available prior to that day, including any previously recorded data from copy-typing sentence-spelling trials in which the decoded sentence had a CER of 0.0. Because our recording sessions were not back-to-back days, the most recent data available for training a new classifier was always at least 3 days prior to a given session (e.g. if the next recording session was on day 4, the most recent data would be from day 1, with no recording on days 2 and 3). We never updated models mid-session; we performed all real-time sentence-spelling evaluations without day-of model recalibration.

Method S4. Language modeling

n-gram modeling

During the beam-search process, as we were updating each beam with a new character, we used a trigram language model because it was reliable while also being capable of producing predictions more quickly than a large neural network-based language model.

The basic n-gram formulation is defined as having the probability of a word w_k in position k as:

$$p(w_k \mid w_{k-1}, ..., w_{k-n+1}) = \frac{C(w_k, ..., w_{k-n+1})}{C(w_{k-1}, ..., w_{k-n+1})}, \tag{S9}$$

where C is a function that counts the number of times each n-gram happens in a corpus.

Improved n-gram modeling can be achieved with back-off and discounting [16]. Back-off refers to using lower-order n-gram models to estimate the probability of higher-order n-grams, since high-order n-grams can be sparse. The n-gram probability $p(w_i \mid w_{i-n+1}^{i-1})$ directly depends on the lower-order n-gram $p(w_i \mid w_{i-n+2}^{i-1})$ (i.e. trigram probabilities depend on bigram and unigram probabilities), as shown in Equation S10. Discounting is a form of regularization of the n-gram probability distribution in which a constant number is removed from the count of each n-gram prior to computing the n-gram probabilities, and the probability mass that was removed in this manner is redistributed through a weighted lower-order n-gram model. For more details, see [17].

We used the following formulation to implement back-off with discounting:

$$p(w_i \mid w_{i-n+1}^{i-1}) = \frac{\max(C(w_{i-n+1}^{i}) - \delta, 0)}{\sum\limits_{w_i} C(w_{i-n+1}^{i})} + \alpha(w_{i-n+1}^{i-1}) p(w_i \mid w_{i-n+2}^{i-1}). \tag{S10}$$

Here, δ is the discount factor and $\alpha(w_{i-n+1}^{i-1})$ is defined as:

$$\alpha(w_{i-n+1}^{i-1}) = \frac{\delta N_{1+}(w_{i-n+1}^{i-1})}{\sum\limits_{w_i} C(w_{i-n+1}^{i})}, \tag{S11}$$

where N_{1+} represents the number of unique words that appear after the preceding $n-1$ words (the number of times the max selects something non-zero in equation S10). Whenever $\sum\limits_{w_i} C(w_{i-n+1}^{i}) = 0$, we use the lower-order model probability directly to avoid division by 0.

We also used Kneser-Ney smoothing ([18]) to improve the unigram model implicit in S10, replacing it with word fertility, which represents the number of distinct context types that a word occurs in. Using word context fertility, we can write the following proportion:

$$p(w) \propto |\{w' : C(w', w) > 0\}|, \tag{S12}$$

where w' is the word fertility and $|\cdot|$ refers to the cardinality operation.

We can now rewrite our unigram model as:

$$p(w) = \frac{|\{w' : C(w', w) > 0\}| + \alpha_{\text{kn}}}{\sum\limits_{w \in \mathcal{V}} |\{w' : C(w', w) > 0\}| + N\alpha_{\text{kn}}}, \tag{S13}$$

where $\mathcal{V}$ is the set of words in the training vocabulary, N is the total number of words in the vocabulary, and α_{kn} is a smoothing hyperparameter that prevents unseen words from having a probability of 0 and infrequent words from being penalized too heavily. In practice, we defined a fixed discount factor $\delta = 0.9$ and a fixed Kneser-Ney smooothing factor $\alpha_{\text{kn}} = 0.003$.

We used two corpora to train the language model: `nltk`'s Twitter corpus [19] and the Cornell movies corpus [20]. We selected these two corpora because of the casual and conversational nature of their speech content. With any given vocabulary, we trained the n-gram model on all of the trigrams from both corpora that were composed solely of words from that vocabulary. Before training, we inserted two start-of-sentence tokens before the start of each sentence in both corpora to enable modeling of sentence starts during inference.

Sentence-finalization language model

To score sentences after finalization during sentence spelling, we used the DistilGPT-2 neural network-based language model [21, 22], which is based on OpenAI's GPT-2 language model [23] but has fewer parameters. This was implemented using the `lm-scorer` Python package (version 0.4.2).

Method S5. Adapted beam search

As described in the Methods section of the main text, we used an adapted prefix beam search as in [24] to find the transcription ℓ^* containing the sequence of characters (including whitespace characters) that maximizes

$$p_{\text{nc}}(\ell \mid X) p_{\text{lm}}(\ell), \tag{S14}$$

over the set of possible transcriptions ℓ. Here X is the set of windows of neural activity $x_1, ..., x_T$, $p_{\text{nc}}(\ell \mid X)$ is the probability under the neural classifier of ℓ given X, and $p_{\text{lm}}(\ell)$ is the probability of transcription ℓ under a language-model prior. As in [24] we postulated that a language-model prior from an n-gram language model is too constrained, so we de-emphasized it using a weighting parameter (α) and added a word-insertion bonus β to make up for the implicit decreasing of the probability of a sentence ℓ as the number of words increases, revising the expression that the beam search tries to maximize to

$$p_{\text{nc}}(\ell \mid X) p_{\text{lm}}(\ell)^{\alpha} |\ell|^{\beta}, \tag{S15}$$

where $|\ell|$ is the cardinality of the word sequence yielded from transcription ℓ. Both α and β were hyperparameters found via hyperparameter optimization on held-out sentence-spelling data. We used an n-gram language model to approximate $p_{\text{lm}}(\ell)$. The full algorithm is detailed in Algorithm 1.

Sentence finalization

If the probability of the attempted hand movement (the sentence-finalization command) was greater than 80%, the predicted sentence was finalized. Specifically, we pruned the current list of candidate sentences (from the beam search) to remove sentences that contained incomplete or out-of-vocabulary words. We then updated the probability of each remaining candidate sentence ℓ as follows:

$$p_{\text{finalized}}(\ell) = p(\ell) p_{\text{gpt2}}(\ell)^{\alpha_{\text{gpt2}}}, \tag{S16}$$

where $p_{\text{finalized}}(\ell)$ is the finalized probability of sentence ℓ, $p(\ell)$ is the probability of the sentence ℓ under equation S15, $p_{\text{gpt2}}(\ell)$ is the probability of ℓ using Distil-GPT2 [22], and α_{gpt2} is a scaling parameter found through hyperparameter optimization. We then used the most likely sentence ℓ as the finalized sentence.

Hyperparameter optimization

To find the optimal hyperparameters α, β, α_{gpt2}, and B, we collected an optimization dataset containing copy-typing sentence-spelling data recorded across 3 sessions to tune these parameters prior to performance evaluation of the spelling system. During these 3 sessions, the participant attempted to spell 35 of the 75 copy-typing sentences. Of these 35 sentences, there were 15 randomly selected sentences that the participant attempted 10 times, 5 sentences that the participant attempted 9 times, and 15 sentences that the participant attempted once. The remaining 40 sentences were unseen by the participant prior to real-time evaluation. We then used these sentences offline to optimize α, β, α_{gpt2}, and B

Algorithm 1 Constrained beam search. Given T windows of neural activity and $p(c \mid x_{1:T})$ (where c is a character), this algorithm finds the most likely sentence ℓ^* composed of words within a constrained vocabulary $\mathcal{V}$. After a character is added to ℓ to give ℓ^+, we check that the final word in ℓ^+ is in $\mathcal{V}_{\text{partial}}$, which is composed of every possible word and partial word $\in \mathcal{V}$. The function w_{final} extracts all the characters after the final space. To automatically insert spaces, the vocabulary considers every text string in A^+, where $A^+ = A \cup A_{\text{space}}$, A is the set of text strings containing a single English letters ("a", "b", "c", ... , "z"), and A_{space} is the same set as A but with the whitespace character appended after each letter ("a ", "b ", "c ", ... , "z "). We set the probability for a character c with a space equal to $p(c \mid x_i)$ (the probability of that character without the space). Here, let the function $W(\ell)$ segment the sequence of characters ℓ at each space and truncate any characters trailing the last space, yielding a list of completed words in ℓ. Let $p_{\text{lm}}(W(\ell^+) \mid W(\ell))$ give the probability of the last word in ℓ^+ given the $n-1$ preceding words, enabling the use of an n-gram language model. The probability threshold for characters to be considered in the beam search was set to 10^{-3}. B is the beam width (the number of beams used in the beam search).

```
sents = {(∅, 0)}
for i = 1, ... ,T do
    new_sents = {}
    for ℓ, log p(ℓ) ∈ sents do
        for c in (A⁺) do
            if p(c|x_i) < 0.001 then
                continue to next character
            end if
            ℓ⁺ ← append c to ℓ
            if w_final(ℓ⁺) ∈ V_partial then
                if c ∈ A_space then
                    log p(ℓ⁺) ← log p(ℓ) + α log p_lm(W(ℓ⁺) | W(ℓ)) + log p(c | x_i)+
                                β log|W(ℓ⁺)| − β log(max(1, |W(ℓ)|))
                else
                    log p(ℓ⁺) ← log p(ℓ) + log p(c | x_i)
                end if
                add (ℓ⁺, log p(ℓ⁺)) to new_sents
            end if
        end for
    end for
    sents ← B most probable prefixes in new_sents
end for
return most probable prefix in sents
```

by simulating the beam-search process. As with the classifier, we used the Asynchronous Hyperband method [14] with the `Ray` package [15], using `Hyperopt` to suggest the next set of hyperparameters after each iteration. We searched 500 sets of hyperparameters and chose the set that produced the best word error rate to use for the first day of real-time sentence-spelling evaluation. After that first day of evaluation, we re-ran the hyperparameter optimization procedure using only the data collected during that day. We used the hyperparameter values found during this second optimization run during all proceeding real-time sentence-spelling evaluation sessions.

No-beam edge case

For 3 of the copy-typing sentence-spelling trials recorded during the real-time evaluation sessions, the beam search ran out of valid sentences. This occurred if the participant made a mistake such that no letter sequence that could make valid sentence candidates surpassed the threshold for consideration by the beam search (see the "Beam search" section in the Methods section of the main text).

On the first day of the real-time evaluation sessions, if this occurred, we would simply output the most likely letters obtained from the neural classifier (without any spaces). Before the second day of real-time evaluation, we modified the beam-search algorithm to output the most likely sentence candidate at that point (immediately before the beam search contained no valid sentence candidates) and then subsequently output the most likely letters obtained from the neural classifier for the remainder of the trial. Additionally, for the first day of the real-time evaluation sessions, the probability threshold for a letter to be considered in the beam search (see Algorithm 1) was set to 10^{-3}. For the second day of real-time evaluation, we kept the threshold the same, but modified the beam-search algorithm so that if less than 3 letters (and their counterparts with spaces) had probability $> 10^{-3}$, we considered the 13 most likely letters (and their counterparts with spaces) to avoid running out of valid beams.

Supplementary figures

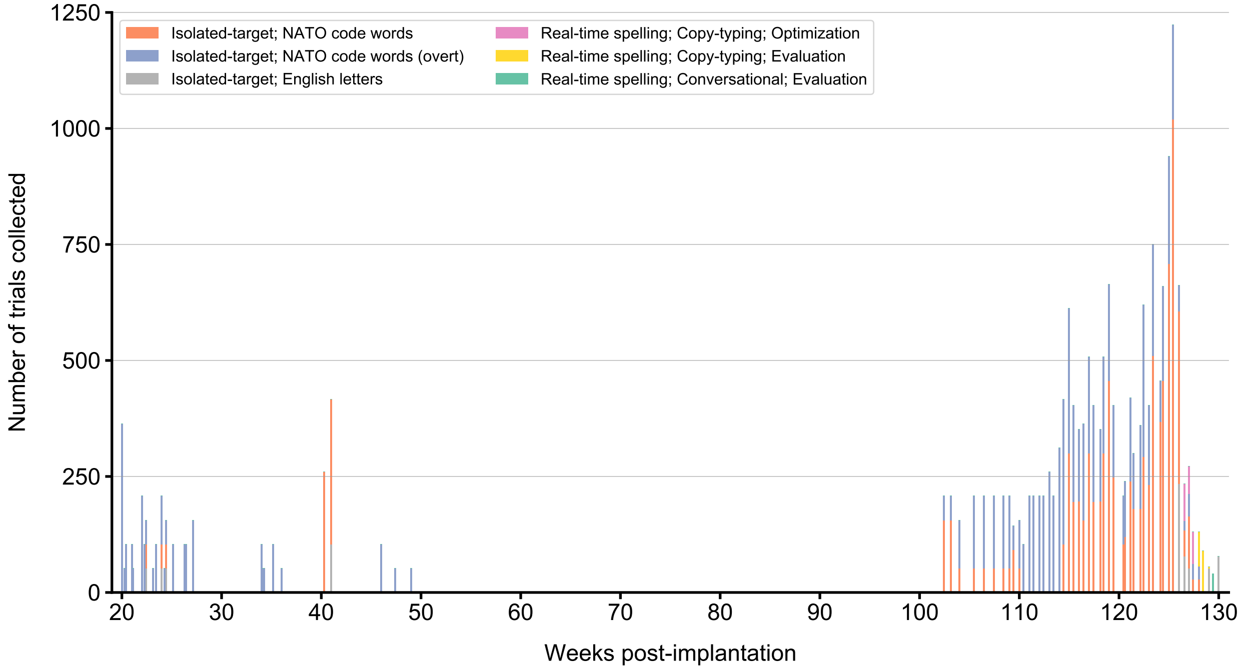

Figure S1. Data collection timeline. Each bar depicts the total number of trials collected on each day of recording. The participant and implant date are the same as in our previous work [2]. If more than one type of dataset was collected in a single day, the bar is colored by the proportion of each dataset collected. Each color represents a specific dataset (as specified in the legend). Datasets vary in task type (isolated-target or real-time sentence spelling), utterance set (English letters, NATO code words (which included the attempted hand squeeze), copy-typing sentences, or conversational sentences), and, for the real-time sentence-spelling datasets, the purpose of the data (for hyperparameter optimization or for performance evaluation). All speech-related trials were associated with silent-speech attempts, except for the dataset with "(overt)" in its legend label. Additionally, 3.06% of trials in this overt dataset were actually recorded during a version of the copy-typing sentence-spelling task in which the participant attempted to overtly produce the code words (see Section S3 for more details). Datasets were collected on an irregular schedule due to external and clinical time constraints that were unrelated to the neural implant. The gap from 55–88 weeks was specifically due to clinical guidelines during the start of the COVID-19 pandemic that limited or prevented in-person recording sessions.

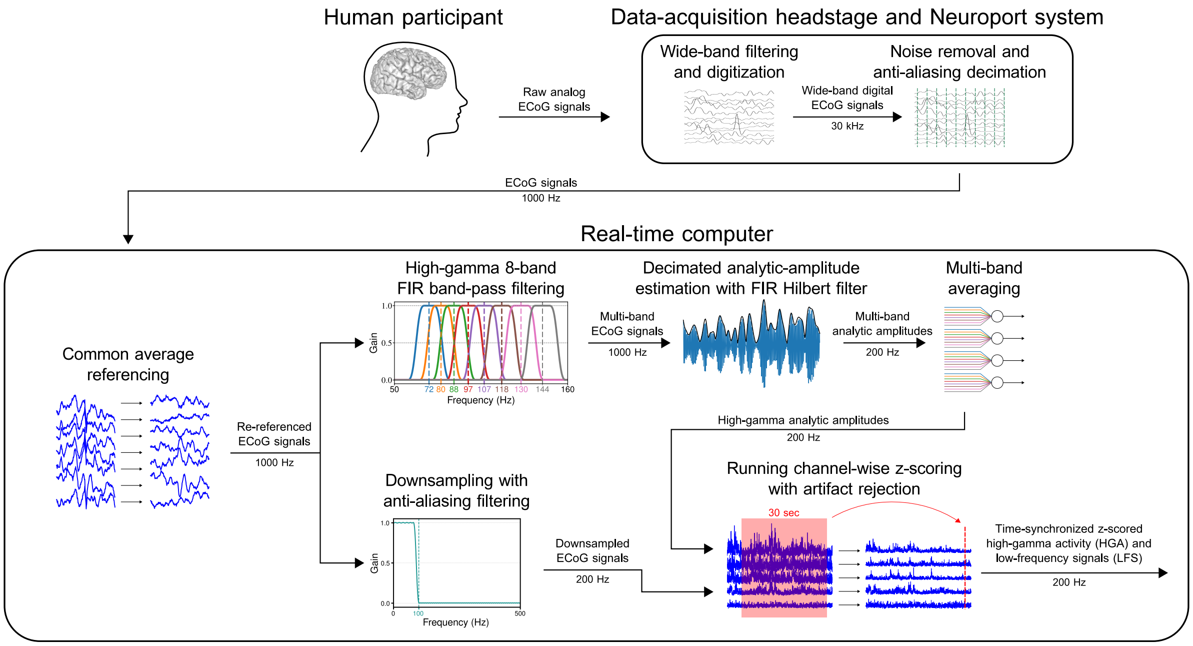

Figure S2. Real-time signal-processing pipeline. A detachable data-acquisition headstage (NeuroPlex E, Blackrock Microsystems) attached to the percutaneous pedestal connector applied a hardware-based wide-band Butterworth filter (between 0.3 Hz and 7.5 kHz) to the ECoG signals, digitized them with 16-bit, 250-nV per bit resolution, and transmitted them at 30 kHz through additional connections to a Neuroport system (Blackrock Microsystems), which processed the signals using software-based line noise cancellation and an anti-aliasing low-pass filter (at 500 Hz). Afterwards, the processed signals were streamed at 1 kHz to a separate computer for further real-time processing and analysis, where we applied a common average reference (across all electrode channels) to each time sample of the ECoG data. The re-referenced signals were then processed in two parallel streams to extract high-gamma activity (HGA) and low-frequency signal (LFS) features. To compute the HGA features, we applied eight 390th-order band-pass finite impulse response (FIR) filters to the re-referenced signals (filter center frequencies were within the high-gamma band at 72.0, 79.5, 87.8, 96.9, 107.0, 118.1, 130.4, and 144.0 Hz). Then, for each channel and band, we used a 170th-order FIR filter to approximate the Hilbert transform. Specifically, for each channel and band, we set the real component of the analytic signal equal to the original signal delayed by 85 samples (half of the filter order) and set the imaginary component equal to the Hilbert transform of the original signal (approximated by this FIR filter) [25]. We then computed the magnitude of each analytic signal at every fifth time sample, yielding analytic amplitude signals at 200 Hz. For each channel, we averaged the analytic amplitude values across the eight bands at each time point to obtain a single high-gamma analytic amplitude measure for that channel. To compute the LFS features, we downsampled the re-referenced signals to 200 Hz after applying a 130th-order anti-aliasing low-pass FIR filter with a cutoff frequency of 100 Hz. We then combined the time-synchronized values from the two feature streams (high-gamma analytic amplitudes and downsampled signals) into a single feature stream. Next, we z-scored the values for each channel and each feature type using Welford's method with a 30-second sliding window [26]. Finally, we implemented a simple artifact-rejection approach to prevent samples with uncommonly large z-score magnitudes from interfering with the running z-score statistics or downstream decoding processes. We adapted this figure from our previous works [2, 27], which implemented similar preprocessing pipelines to compute high-gamma features.

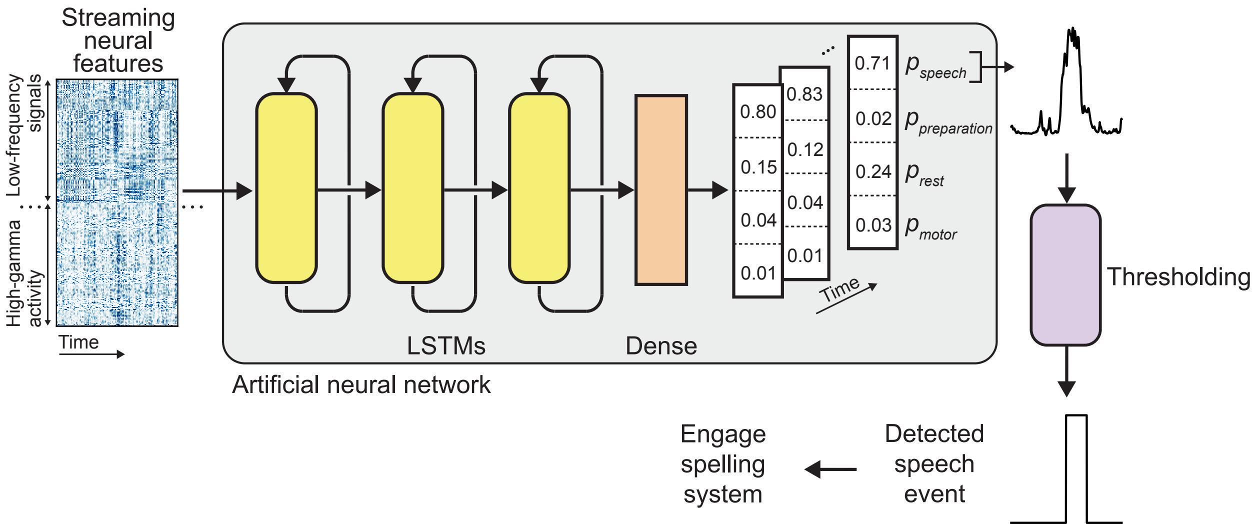

Figure S3. Speech-detection model schematic. To detect silent-speech attempts from the participant's neural activity during real-time sentence spelling, first the z-scored low-frequency signals (LFS) and high-gamma activity (HGA) for each electrode are processed continuously by a stack of 3 long short-term memory (LSTM) layers. Next, a single dense (fully connected) layer projects the latent dimensions of the final LSTM onto the 4 possible classes: speech, speech preparation, rest, and motor. The stream of speech probabilities is then temporally smoothed, probability thresholded, and time thresholded to yield onsets and offsets of full speech events. Once the participant attempts to silently say something and that speech attempt is detected, the spelling system is engaged and the paced spelling procedure begins. The depicted neural features, predicted speech-probability time series (upper right), and detected speech event (lower right) are the actual neural data and detection results for a 5-second time window at the beginning of a trial of the real-time sentence copy-typing task. This figure was adapted from our previous work [2], which implemented a similar speech-detection architecture.

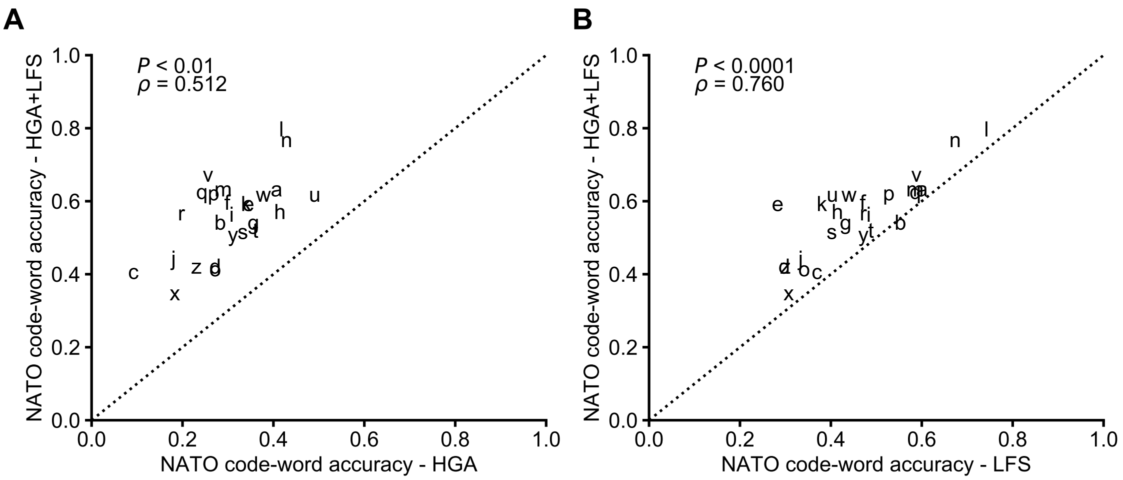

Figure S4. Effects of feature selection on code-word classification accuracy. A. Classification accuracy improves for each code word when using high-gamma activity (HGA) and low-frequency signals (LFS) together (the combined HGA+LFS feature set) instead of only HGA features. The accuracies are significantly correlated with a Spearman rank correlation of 0.512 ($P = 0.0085$, permutation testing with 2000 iterations). **B.** Classification accuracy improves for almost every code word when using HGA+LFS instead of LFS alone. The accuracies are significantly correlated with a Spearman rank correlation of 0.760 ($P \approx 0.00$, permutation testing with 2000 iterations). Because not all possible permutations were tested (the number of possible permutations for 26 elements is 4.03×10^{26}, so we approximate this test with 2000 iterations), the P-value is approximately 0.00 in this case. In both **A** and **B**, code words are represented as lower-case letters and the Spearman rank correlations are shown. The associated P-value was computed via permutation testing. In permutation testing, one group of observations (code-word accuracies for either HGA, LFS, or HGA+LFS) was shuffled before re-computing the correlation between that group of observations and the other group. 2000 iterations were used during permutation testing for each of the two comparisons. The P-value was computed as the proportion of the distribution of correlations computed during permutation testing that were greater in magnitude than the correlation computed on non-shuffled data.

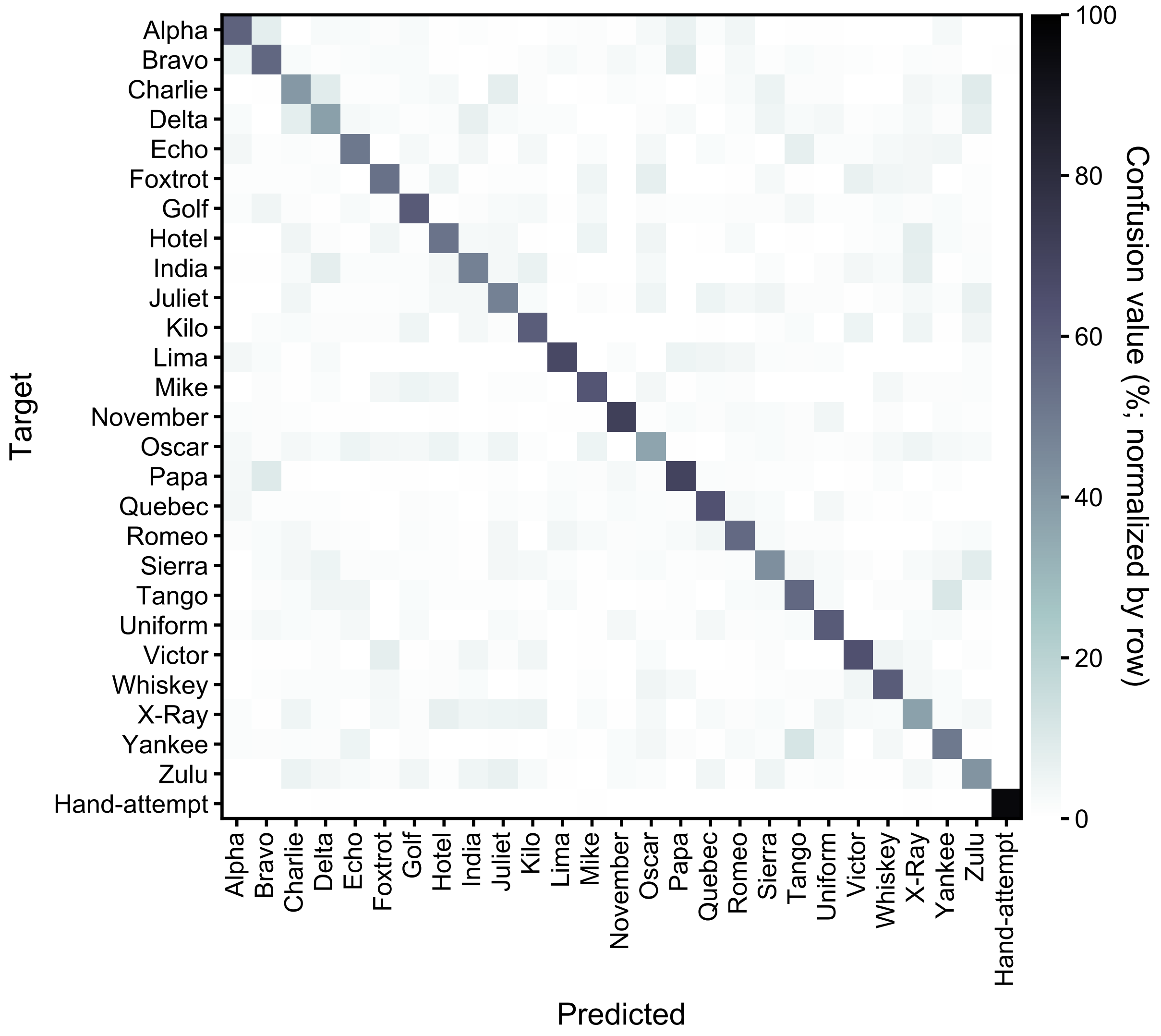

Figure S5. Confusion matrix from isolated-target trial classification using HGA and LFS. Confusion values, computed during offline classification of neural data (using both high-gamma activity and low-frequency signals) recorded during isolated-target trials, are shown for each NATO code word and the attempted hand squeeze. Each row corresponds to a target code word or the attempted hand squeeze, and the value in each column for that row corresponds to the percent of isolated-target task trials that were correctly classified as the target (if the value is along the diagonal) or misclassified ("confused") as another potential target (if the value is not along the diagonal). The values in each row sum to 100%. In general, silent-speech and hand-squeeze attempts were reliably classified. Including both the attempted NATO code word trials and the attempted hand squeeze trials, the 10-fold cross-validated median accuracy was 56.4% with a 99% confidence interval of $[54.3, 58.2]$.

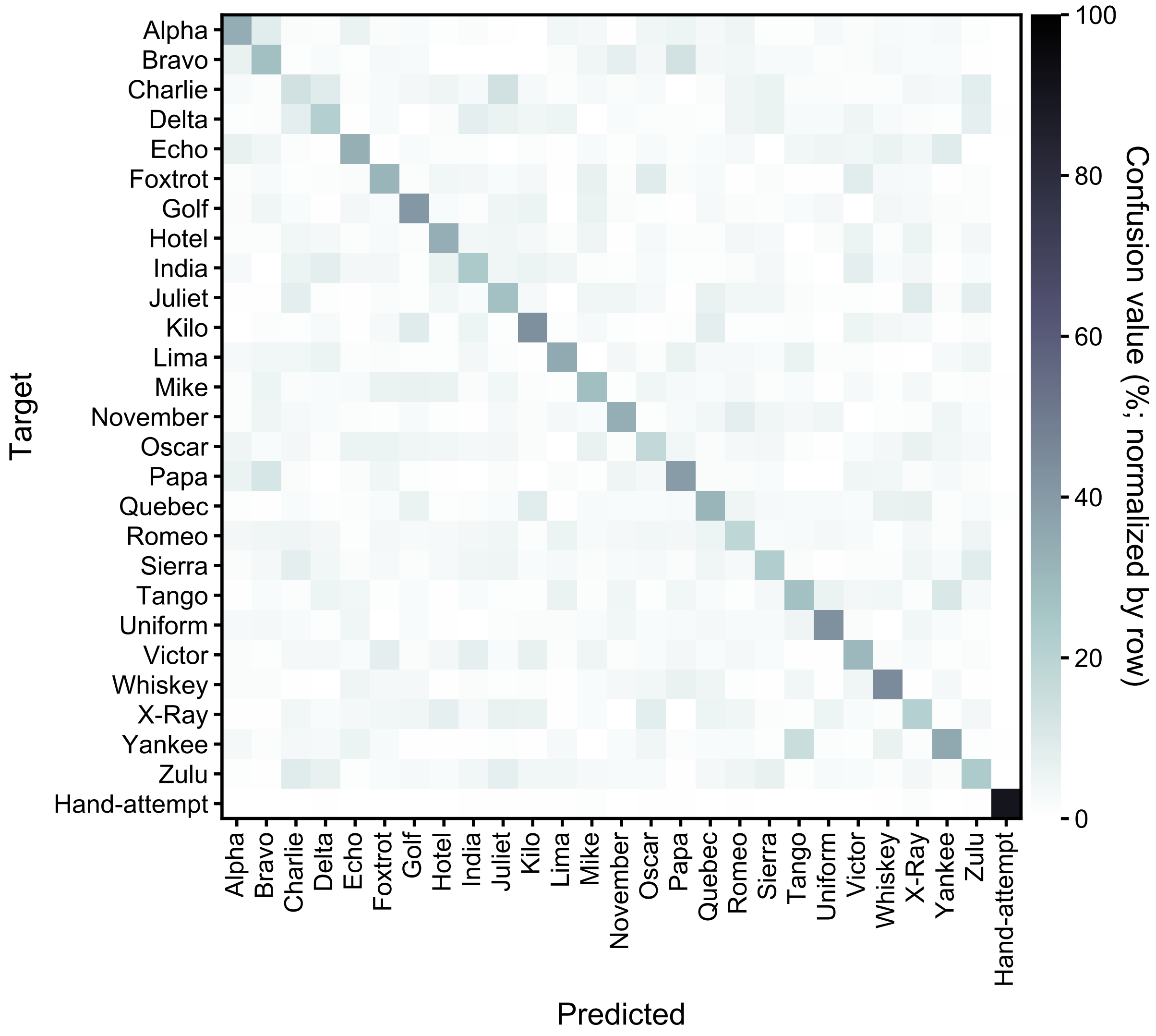

Figure S6. Confusion matrix from isolated-target trial classification using only HGA. Confusion values, computed during offline classification of neural data (using only high-gamma activity) recorded during isolated-target trials, are shown for each NATO code word and the attempted hand squeeze. Each row corresponds to a target code word or the attempted hand squeeze, and the value in each column for that row corresponds to the percent of isolated-target task trials that were correctly classified as the target (if the value is along the diagonal) or misclassified ("confused") as another potential target (if the value is not along the diagonal). The values in each row sum to 100%. Including both the attempted NATO code word trials and the attempted hand squeeze trials, the 10-fold cross-validated median accuracy was 32.7% with a 99% confidence interval of $[32.0, 33.6]$.

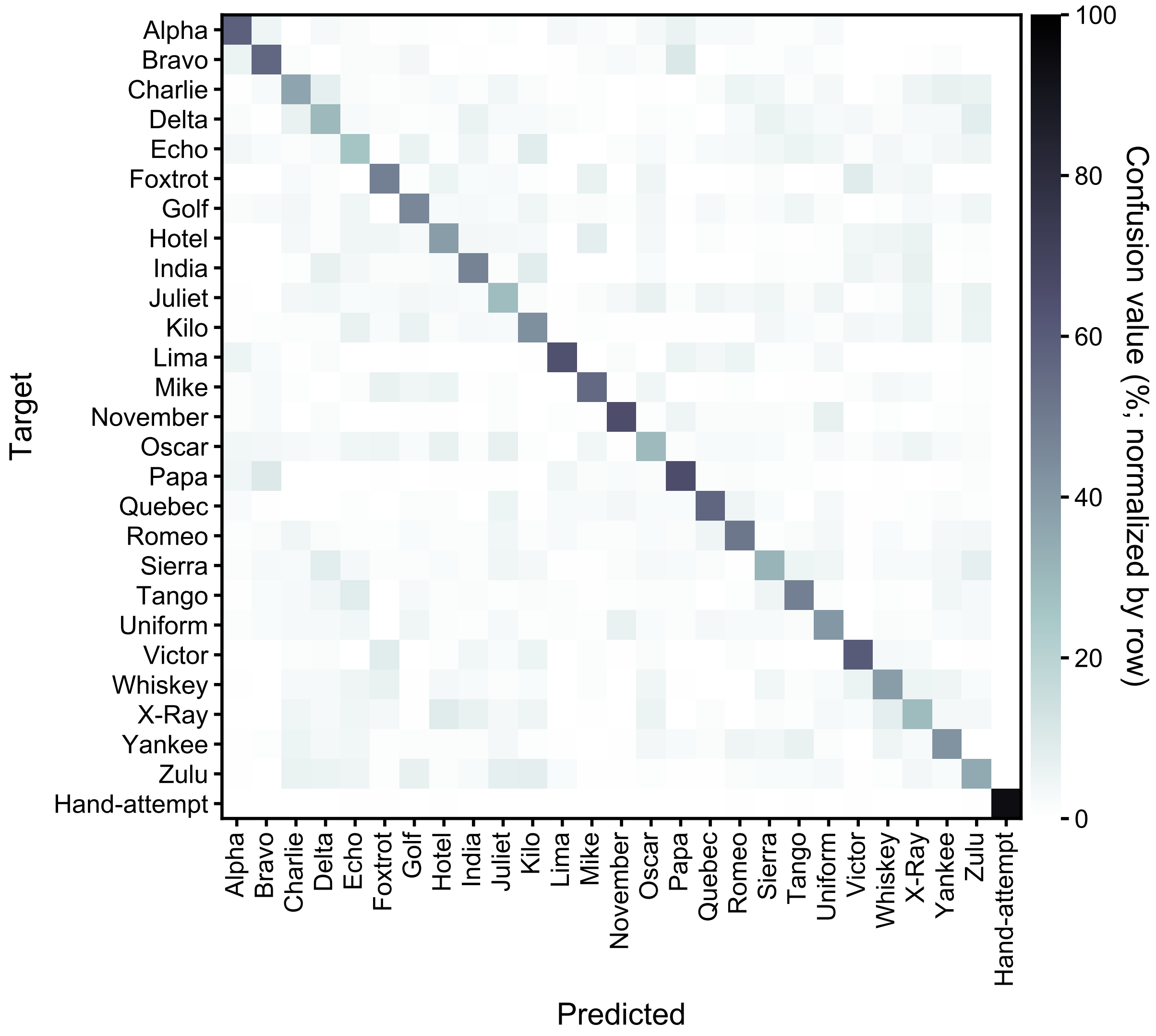

Figure S7. Confusion matrix from isolated-target trial classification using only LFS. Confusion values, computed during offline classification of neural data (using only low-frequency signals) recorded during isolated-target trials, are shown for each NATO code word and the attempted hand squeeze. Each row corresponds to a target code word or the attempted hand squeeze, and the value in each column for that row corresponds to the percent of isolated-target task trials that were correctly classified as the target (if the value is along the diagonal) or misclassified ("confused") as another potential target (if the value is not along the diagonal). The values in each row sum to 100%. Including both the attempted NATO code word trials and the attempted hand squeeze trials, the 10-fold cross-validated median accuracy was 48.2% with a 99% confidence interval of $[42.9, 49.7]$.

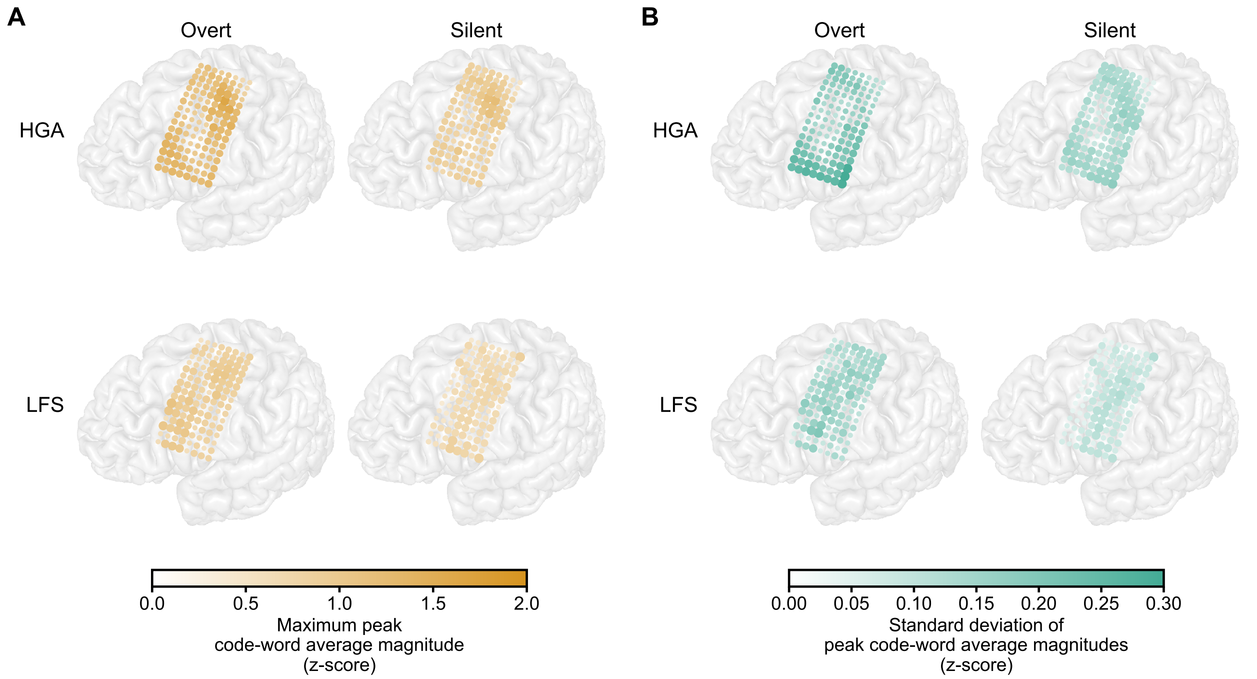

Figure S8. Neural-activation statistics during overt- and silent-speech attempts. A. Each image shows an MRI reconstruction of the participant's brain overlaid with electrode locations and the maximum neural activations for each electrode, type of speech attempt (overt or silent), and feature type (high-gamma activity (HGA) or low-frequency signals (LFS)), measured as maximum peak code-word average magnitudes. To calculate these values, the trial-averaged neural-feature time series was computed for each code word, electrode, type of speech attempt, and feature type using the isolated-target dataset (for each trial, the 2.5-second time window after the go cue was used). Then, the peak magnitude (maximum of the absolute value) of each of these trial-averaged time series was determined. The maximum peak code-word average magnitude for each electrode, type of speech attempt, and feature type was then computed as the maximum value of these peak magnitudes across code words for each combination. The two columns show the values for each type of speech attempt (overt then silent), and the two rows show the values for each feature type (HGA then LFS). **B.** The standard deviation of peak code-word average magnitudes. Here, the standard deviation (instead of the maximum used in **A**) of the peak average magnitudes across the code words for each electrode, type of speech attempt, and feature type is computed and plotted, depicting how much the magnitudes varied across speech targets for that combination. For **A** and **B**, the color of each plotted electrode indicates the true associated value for that electrode, and the size of each electrode depicts the associated value for that electrode relative to the values for the other electrodes (for a given type of speech attempt and feature type).

Supplementary tables

Supplementary Table S1. Copy-typing task sentences.

Target sentence	Decoded sentence in first trial	Decoded sentence in second trial
good morning	good morning	good for legs
you have got to be kidding	you have got to be kidding a	you have got to be kidding
what do you mean	what do you mean	what do you mean
good to see you	i do i leave you	good to see you
i think this is pretty good	i think this is pretty good	i think they is pretty good
i will check	i will check	i will the it
thank you	thank you	thank you
please sit down	please sit down	please believe
we have to stop	we have to stop	we have to stop
hand that to me please	hand that time please	have that time always
i know what you mean	i know what you mean	i know what you mean
what time is it	what time is it	what time is it
sit over here with me	sit over here with me	sit over here with me
no thanks	no thanks	not happen
you never know	you never know	you never know
great to see you again	great to show my case in	great to stay in town
forget about it	forget about it	forget about it
could you repeat what you said	dog lie on repeat what you said	could you repeat what you said
where do you live	where do you live	where do you live
do not be afraid to ask me questions	do not be afraid to ask me questions	do not be afraid to ask me questions
i cannot believe it	i can not believe it	i can not believe it
thanks for telling me	thank for reading me	thanks for telling me
i do not want that	i do not want that	i do not want that
that is wonderful	that is work from a	that is wonderful
what do you think about that	what do you think about that	what do you think about that
thank you very much	though it very much	thank you very much
i am glad you are here	i am glad you are here	i am glad you are here
how are you doing	how are you doing	how are you doing
i agree	i agree	i agree
i am okay	i am okay	i am okay
tell me what you are doing	tell me what your telling	tell me what you are doing
how long did it take	how long did it take	how long did it take
is there anything i can do	is there a nothing i can do	is there anything i can do
how are things going for you	how are things gives for you	how are things going for you
do you know what he did	do you know on the ice	do you know what he did
was there something else	was there to be a high else	was there something else
where are you going	while are you doing	where are you going
who is that	who is that	why is that
tell me about your family	tell me about your family	tell me about your family
i could probably do better	i could probably do better	i could probably do better
you can say that again	you can say that again	you can say that open
i am sorry to hear that	i am to get to hear that	i am sorry to hear that
will i see you later	will i see you later	well i keep by later
i am doing well	i am doing well	i am doing fine
can that wait until another time	can that wait until another time	can that wait until another time
how much more is there	how much more is there	how much were in there
come talk with me	come talk with me	some take with me
that will be fun	that will be fun	that will be fun
how often do you do this	how often do you do this	how often do you do this
how much will it cost	how much will it cost	how much will it cost
bring that over here	clinic hat for hat	bring that ever here
turn it off	turn it off	turn it off
i remember the last time i did that	i remember the last time i did that	i remember to plan new me i did that
i was just kidding	i was mike kidding	i was just kidding
i will meet you there	i will meet you there	i will meet you to eat
i do not really remember	i do not really remember	ddonoyrballyrlrefbhrh
i feel cold	i feel weird	i feel cold
excuse me for interrupting	excuse me for interrupt any	excuse me for interrupting
you are not going to believe this	you plan to go in on a bit love this	ypuaranpdggingloavlinesoeb
do you understand what i mean	do you understand what i mean	do you understand what i mean
what are you talking about	what are you talking about	what are you talking about
which one is it	which one edit	which one is it
would you like to go with me	a all i was like the white me	would you like to go with me
i do not understand	i do not understand	i do not understand
of course i do	of course its	of course him
anything is possible	anything is possible	anything is possible
do not do that again	do not do that again	do not do that again
let me see that	let me see that	let me see that
what have you been doing	what have you been doing	what have you been doing
i had a great time	i had a great time	what a great time
easy for you to say	easy for you to say	easy for you to say
i want to go	i want to go	i want to go
how do you feel	how do you feel	how do you feel
that is all right	that is all right	that is all right
i told you i do not know	i told you i do not know	i told you i do not know

Supplementary Table S2. Statistical comparisons of character error rates across decoding-framework conditions.

Statistical comparison[1]	$\lvert z\text{-value} \rvert$	P-value (corrected)[2]
Chance vs. Only Neural Decoding	7.09	8.08×10^{-12}
Chance vs. + Vocab. Constraints	7.09	8.08×10^{-12}
Chance vs. + LM (Real-time results)	7.09	8.08×10^{-12}
Only Neural Decoding vs. + LM (Real-time results)	6.94	1.21×10^{-11}
+ Vocab. Constraints vs. + LM (Real-time results)	5.53	6.34×10^{-8}
Only Neural Decoding vs. + Vocab. Constraints	4.51	6.37×10^{-6}

[1] Each comparison is a two-sided Wilcoxon Rank-Sum test across 34 real-time spelling blocks.
[2] 6-way Holm-Bonferroni correction for multiple comparisons.

Supplementary Table S3. Statistical comparisons of word error rates across decoding-framework conditions.

Statistical comparison[1]	$\lvert z\text{-value} \rvert$	P-value (corrected)[2]
Chance vs. + LM (Real-time results)	7.09	8.08×10^{-12}
Only Neural Decoding vs. + LM (Real-time results)	7.09	8.08×10^{-12}
Chance vs. + Vocab. Constraints	6.70	8.16×10^{-11}
Only Neural Decoding vs. + Vocab. Constraints	6.61	1.19×10^{-10}
+ Vocab. Constraints vs. + LM (Real-time results)	6.11	2.01×10^{-9}

[1] Each comparison is a two-sided Wilcoxon Rank-Sum test across 34 real-time spelling blocks.
[2] 6-way Holm-Bonferroni correction for multiple comparisons.

Supplementary Table S4. Statistical comparisons of classification accuracy across neural-feature types.

Statistical comparison[1]	$\lvert z\text{-value} \rvert$	P-value (corrected)[2]
HGA vs. LFS	3.78	4.71×10^{-4}
HGA vs. HGA+LFS	3.78	4.71×10^{-4}
LFS vs. HGA+LFS	3.78	4.71×10^{-4}

[1] Each comparison is a two-sided Wilcoxon Rank-Sum test across 10 cross-validation folds.
[2] 6-way Holm-Bonferroni correction for multiple comparisons.

Supplementary Table S5. Statistical comparisons of the number of principal components required to explain more than 80% of the variance in the spatial dimension across neural-feature types.

Statistical comparison[1]	$\mid z\text{-value} \mid$	P-value (corrected)[2]
HGA vs. LFS	12.22	7.57×10^{-34}
HGA vs. HGA+LFS	12.22	7.57×10^{-34}
LFS vs. HGA+LFS	12.02	2.66×10^{-33}

[1] Each comparison is a two-sided Wilcoxon Rank-Sum test across 100 bootstrap iterations.
[2] 3-way Holm-Bonferroni correction for multiple comparisons.

Supplementary Table S6. Statistical comparisons of the number of principal components required to explain more than 80% of the variance in the temporal dimension across neural-feature types.

Statistical comparison[1]	$\lvert z\text{-value} \rvert$	P-value (corrected)[2]
HGA vs. LFS	12.22	7.57×10^{-34}
LFS vs. HGA+LFS	12.22	7.57×10^{-34}
HGA vs. HGA+LFS	2.68	0.007 27

[1] Each comparison is a two-sided Wilcoxon Rank-Sum test across 100 bootstrap iterations.
[2] 3-way Holm-Bonferroni correction for multiple comparisons.

Supplementary Table S7. Statistical comparisons of classification accuracy across attempted-speech types with various training schemes.

Group 1		Group 2			P-value
Train	Test	Train	Test	$\lvert z\text{-value} \rvert$	(corrected[2])
Silent	Silent	Silent	Overt	3.78	4.4×10^{-3}
Silent	Silent	Overt	Overt	3.78	4.4×10^{-3}
Silent	Silent	Overt	Silent	3.78	4.4×10^{-3}
Silent	Silent	Overt pre-train, silent fine-tune	Silent	3.78	4.4×10^{-3}
Silent	Silent	Silent pre-train, overt fine-tune	Overt	3.78	4.4×10^{-3}
Silent	Overt	Overt	Overt	3.78	4.4×10^{-3}
Silent	Overt	Overt pre-train, silent fine-tune	Silent	3.78	4.4×10^{-3}
Silent	Overt	Overt pre-train, silent fine-tune	Overt	3.78	4.4×10^{-3}
Silent	Overt	Silent pre-train, overt fine-tune	Silent	3.78	4.4×10^{-3}
Silent	Overt	Silent pre-train, overt fine-tune	Overt	3.78	4.4×10^{-3}
Overt	Overt	Overt	Silent	3.78	4.4×10^{-3}
Overt	Overt	Overt pre-train, silent fine-tune	Overt	3.78	4.4×10^{-3}
Overt	Overt	Silent pre-train, overt fine-tune	Silent	3.78	4.4×10^{-3}
Overt	Silent	Overt pre-train, silent fine-tune	Silent	3.78	4.4×10^{-3}
Overt	Silent	Overt pre-train, silent fine-tune	Overt	3.78	4.4×10^{-3}
Overt	Silent	Silent pre-train, overt fine-tune	Silent	3.78	4.4×10^{-3}
Overt	Silent	Silent pre-train, overt fine-tune	Overt	3.78	4.4×10^{-3}
Overt pre-train, silent fine-tune	Silent	Overt pre-train, silent fine-tune	Overt	3.78	4.4×10^{-3}
Overt pre-train, silent fine-tune	Silent	Silent pre-train, overt fine-tune	Silent	3.78	4.4×10^{-3}
Overt pre-train, silent fine-tune	Overt	Silent pre-train, overt fine-tune	Overt	3.78	4.4×10^{-3}
Silent pre-train, overt fine-tune	Silent	Silent pre-train, overt fine-tune	Overt	3.78	4.4×10^{-3}
Overt pre-train, silent fine-tune	Silent	Silent pre-train, overt fine-tune	Overt	3.70	4.4×10^{-3}
Silent	Overt	Overt	Silent	3.17	8.99×10^{-3}
Overt pre-train, silent fine-tune	Overt	Silent pre-train, overt fine-tune	Silent	2.76	2.9×10^{-2}
Overt	Overt	Silent pre-train, overt fine-tune	Overt	2.65	3.26×10^{-2}
Overt	Overt	Overt pre-train, silent fine-tune	Silent	2.57	3.26×10^{-2}
Silent	Silent	Overt pre-train, silent fine-tune	Overt	1.51	2.61×10^{-1}
Silent	Silent	Silent pre-train, overt fine-tune	Silent	0.76	4.5×10^{-1}

[1] Each comparison is a two-sided Wilcoxon Rank-Sum test across 10 cross-validation folds.
[2] 28-way Holm-Bonferroni correction for multiple comparisons.

Supplementary Table S8. Hyperparameter definitions and values.

Model	Hyperparameter description	Search-space type[1]	Value range	Optimal values[2]
Speech detector	Smoothing size	Uniform (int)	$[1, 80]$	78
	Probability threshold	Uniform	$[0.1, 0.9]$	0.304
	Time threshold duration	Uniform (int)	$[25, 150]$	105
Word classifier	Number of GRU layers	Uniform (int)	$[1, 4]$	2
	Nodes per GRU layer	Uniform (int)	$[128, 512]$	274
	Dropout fraction	Uniform	$[0.3, 0.8]$	0.545
	Convolution kernel size and skip	Uniform (int)	$[1, 10]$	4
	Jitter amount (seconds), j	Uniform	$[0.0, 2.0]$	0.474
	Additive noise level, σ_n	Uniform	$[0.0, 1.0]$	0.0027
	Scale min., α_{min}	Uniform	$[0.8, 1.0]$	0.955
	Scale max., α_{max}	Uniform	$[1.0, 1.2]$	1.07
	Max. temporal-masking length (seconds), b	Uniform	$[0.00, 1.35]$	0.871
	Temporal masking probability, p	Uniform	$[0.0, 0.5]$	0.0478
	Channel-wise noise, σ_c	Uniform	$[0.0, 1.0]$	0.0283
Beam search	Language-model scaling factor, α	Uniform	$[0.01, 1.0]$	$(0.642, 0.744)$
	Word-insertion weight, β	Uniform	$[0.0, 30.0]$	$(4.03, 10.5)$
	Number of beams maintained, B	Uniform (int)	$[0, 750]$	$(457, 739)$
	Distil-GPT2 scaling factor, α_{gpt2}	Uniform	$[0.0, 100.0]$	$(1.53, 1.13)$

[1] "Uniform (int)" indicates that hyperparameter values were forced to be integers.
[2] For the language modeling and beam-search hyperparameters, two values are listed: the first is the optimal value found when optimizing on the copy-typing sentence-spelling trials prior to the first day of sentence-spelling evaluations (used during this first day), and the second is the optimal value found when optimizing on the copy-typing sentence-spelling trials from the first day of sentence-spelling evaluations (used for the second day and all subsequent days).

Supplementary references

1. Paszke A, Gross S, Massa F, et al. PyTorch: An Imperative Style, High-Performance Deep Learning Library. In: Advances in Neural Information Processing Systems 32. Ed. by Wallach H, Larochelle H, Beygelzimer A, d'Alché-Buc F, Fox E, and Garnett R. Curran Associates, Inc., 2019:8024–35.
2. Moses DA, Metzger SL, Liu JR, et al. Neuroprosthesis for Decoding Speech in a Paralyzed Person with Anarthria. New England Journal of Medicine 2021;385:217–27.
3. Kingma DP and Ba J. Adam: A Method for Stochastic Optimization. arXiv:1412.6980 2017.
4. Bergstra J, Yamins DLK, and Cox DD. Making a Science of Model Search: Hyperparameter Optimization in Hundreds of Dimensions for Vision Architectures. Icml 2013:115–23.
5. Cho K, Van Merriënboer B, Bahdanau D, and Bengio Y. On the properties of neural machine translation: Encoder-decoder approaches. arXiv preprint arXiv:1409.1259 2014.
6. Hochreiter S and Schmidhuber J. Long short-term memory. Neural computation 1997;9:1735–80.
7. Chung J, Gulcehre C, Cho K, and Bengio Y. Empirical evaluation of gated recurrent neural networks on sequence modeling. arXiv preprint arXiv:1412.3555 2014.
8. Hinton GE, Srivastava N, Krizhevsky A, Sutskever I, and Salakhutdinov RR. Improving neural networks by preventing co-adaptation of feature detectors. arXiv preprint arXiv:1207.0580 2012.
9. Kingma DP and Ba J. Adam: A method for stochastic optimization. arXiv preprint arXiv:1412.6980 2014.
10. Paszke A, Gross S, Massa F, et al. PyTorch: An Imperative Style, High-Performance Deep Learning Library. In: Advances in Neural Information Processing Systems 32. Ed. by Wallach H, Larochelle H, Beygelzimer A, d'Alché-Buc F, Fox E, and Garnett R. Curran Associates, Inc., 2019:8024–35. URL: `http://papers.neurips.cc/paper/9015-pytorch-an-imperative-style-high-performance-deep-learning-library.pdf`.
11. Krizhevsky A, Sutskever I, and Hinton GE. Imagenet classification with deep convolutional neural networks. Advances in neural information processing systems 2012;25:1097–105.
12. Reed CJ, Metzger S, Srinivas A, Darrell T, and Keutzer K. Selfaugment: Automatic augmentation policies for self-supervised learning. In: Proceedings of the IEEE/CVF Conference on Computer Vision and Pattern Recognition. 2021:2674–83.
13. Willett FR, Avansino DT, Hochberg LR, Henderson JM, and Shenoy KV. High-performance brain-to-text communication via handwriting. Nature 2021;593:249–54.
14. Li L, Jamieson K, Rostamizadeh A, et al. Massively parallel hyperparameter tuning. 2018.

15. Moritz P, Nishihara R, Wang S, et al. Ray: A distributed framework for emerging AI applications. In: 13th USENIX Symposium on Operating Systems Design and Implementation (OSDI 18). 2018:561–77.

16. Chen SF and Goodman J. An empirical study of smoothing techniques for language modeling. Computer Speech & Language 1999;13:359–94.

17. Jurafsky D and Martin JH. Speech and language processing. Vol. 3. US: Prentice Hall 2014.

18. Kneser R and Ney H. Improved backing-off for m-gram language modeling. In: 1995 international conference on acoustics, speech, and signal processing. Vol. 1. IEEE. 1995:181–4.

19. Bird S, Klein E, and Loper E. Natural language processing with Python: analyzing text with the natural language toolkit. " O'Reilly Media, Inc.", 2009.

20. Danescu-Niculescu-Mizil C and Lee L. Chameleons in imagined conversations: A new approach to understanding coordination of linguistic style in dialogs. In: Proceedings of the Workshop on Cognitive Modeling and Computational Linguistics, ACL 2011. 2011.

21. Sanh V. Smaller, faster, cheaper, lighter: Introducing DistilBERT, a distilled version of BERT. 2019. URL: `https://medium.com/huggingface/distilbert-8cf3380435b5`.

22. Sanh V, Debut L, Chaumond J, and Wolf T. DistilBERT, a distilled version of BERT: smaller, faster, cheaper and lighter. 2020. arXiv: `1910.01108 [cs.CL]`.

23. Radford A, Wu J, Child R, Luan D, Amodei D, Sutskever I, et al. Language models are unsupervised multitask learners. OpenAI blog 2019;1:9.

24. Hannun AY, Maas AL, Jurafsky D, and Ng AY. First-pass large vocabulary continuous speech recognition using bi-directional recurrent dnns. arXiv preprint arXiv:1408.2873 2014.

25. Romero DET and Jovanovic G. Digital FIR Hilbert Transformers: Fundamentals and Efficient Design Methods. In: MATLAB - A Fundamental Tool for Scientific Computing and Engineering Applications - Volume 1. 2012:445–82. URL: `http://www.intechopen.com/books/matlab-a-fundamental-tool-for-scientific-computing-and-engineering-applications-volume-1/digital-fir-hilbert-transformers-fundamentals-and-efficient-design-methods`.

26. Welford BP. Note on a Method for Calculating Corrected Sums of Squares and Products. Technometrics 1962;4:419–9.

27. Moses DA, Leonard MK, Makin JG, and Chang EF. Real-time decoding of question-and-answer speech dialogue using human cortical activity. Nature Communications 2019;10:3096.

Generalizable spelling using a speech neuroprosthesis in an individual with severe limb and vocal paralysis

This supplement contains the following items relating to the clinical-trial protocol registered on ClinicalTrials.gov (with ID NCT03698149), which the present work was performed under:

1. Original protocol (registered on October 4, 2018)
2. Final protocol (updated on June 22, 2020)
3. Summary of changes between the final and original protocols
4. Note about the exploratory nature of the clinical trial

CLINICAL PROTOCOL
(original)

Title: A High-Performance ECoG-based Neural Interface for Communication and Neuroprosthetic Control

Study Sponsors/Investigators:

Redacted

Protocol Synopsis

**Title**	**A High-Performance ECoG-based Neural Interface for Communication and Neuroprosthetic Control**
**Study Phase**	Phase I
**Device(s)**	**Device Information:** Devices to be used in this study are grouped below according to FDA approval.<br><br>**Cleared for temporary (<30 days) recording and monitoring of brain electrical activity under 510k:**<br>• **NeuroPort Array, PN 6248 (K070272, K110010)**<br>• **NeuroPort System, PN 5416 (K060523, K090957)**<br><br>**Cleared for temporary (<30 day) use with recording, monitoring, and stimulation equipment for the recording, monitoring and stimulation of electrical signals on the surface of the brain under 510k:**<br>• **PMT Subdural Cortical Electrodes, Model #2110TX-128-005 (K082474)**<br><br>The Blackrock Microsystems Neuroport Array connector pedestal, a subcomponent of the Blackrock Microsystems NeuroPort Array, will be laser bonded to the PMT Subdural Cortical Electrode by Blackrock Microsystems. As documented below, this specific approach has already been tested in non-human primates (over at least an 18-month period, see below regarding published report).<br><br>**Request for off-label use of the combined investigational device for 1 year, for the equivalent indication of recording and monitoring of brain electrical activity.**
**Indication**	Adults with neurological disorders (e.g. amyotrophic lateral sclerosis/ALS, spinal cord injury, multiple sclerosis, stroke) often develop disorders of movement and communication. We aim to determine the feasibility of ECoG based brain computer interface control of complex neuroprosthetic devices.
**Sponsor Contacts**	*Redacted*
**Data Safety Monitor Board (DSMB)**	*Redacted*

**Treatment**	The Blackrock Microsystems NeuroPort Array connector pedestal, NeuroPort System, and PMT Subdural Cortical Electrodes are currently cleared for monitoring of patients for up to 30 days. Here we to aim to use these systems combined for at least a 1-year period in subjects with neurological illness and disorders of communication to test feasibility for both neuroprosthetic control and for decoding speech from neural activity.
**Study Sites**	• *Redacted*
**Study Design**	This is a **single-center early feasibility study** of the use of an ECoG-based neural interface for testing the feasibility of using ECoG signals to control complex devices for motor and speech control in adults affected by neurological disorders of movement.<br><br>A PMT Subdural Cortical Electrode array, bonded to the Blackrock Microsystems NeuroPort Array pedestal, will be surgically placed directly on the brain surface over the motor and language cortices of subjects with disorders of motor control. After implantation of the electrode and pedestal, the Neuroport Biopotential Processing System will be connected to the Neuroport Array pedestal to monitor and record neural signals. With this ECoG-based neural interface, study patients will undergo training and assessment of their ability to control a wearable hand robotic exoskeleton and to determine if ECoG brain signals can be decoded for language communication. This will be performed in two phases.<br><br><u>Phase 1: Optimize BCI system</u><br>In Phase 1, we will optimize the entire system to reliably detect neural activity to ensure that the recorded signals are stable and free of artifacts. Moreover, we will ensure that the neural signals are converted in real-time into cursor movements. During this phase, we will primarily examine cursor based control, decoding of parameters and 'disembodied' control (i.e. the subjects' arm will not interact with the mechanical system). This phase will be conducted in the outpatient office setting and/or the patient's home environment, based on patient preference and needs. We anticipate that this phase will take approximately 1 month; however, this may be longer or shorter for each subject depending on the level of control achieved.<br><br><u>Phase 2: Testing of BCI Control</u><br>In Phase 2, we will test feasibility for both neuroprosthetic control and for decoding speech from neural activity. We will begin to perform experimental testing with the system for control of a custom wearable hand exoskeleton robot that

	meets criteria for a non-significant risk device. As outlined in the sections below, we have multiple safety features to ensure that there is minimal risk for injury during the embodiment phase (i.e. the subjects arm is interacting with the exoskeleton). Throughout this period, neural signals will be recorded and analyzed, and tasks will be performed toward the development, assessment, and improvement of the neural interface system. We will assess quality of performance using kinematic parameters while performing required tasks. We will analyze stability of neural recordings and performance. To measure stability of the neural representation we will analyze the neural correlates of imagined movements. We will also assess changes in spatial correlation scales and other redundancy measures during learning and stable performance. We anticipate that testing will be conducted in the outpatient office or home setting based on the patient's preference and needs.<br><br>In this phase, we will also continue to perform experimental testing with the system for control of a virtual communicating interface. Throughout this period, neural signals will be recorded and analyzed, and tasks will be performed toward the development, assessment, and improvement of the communication interface.
**Objectives**	In eight patients with severe disorders of movement control, we will surgically implant PMT Subdural Cortical Electrodes on the brain surface over the motor and language cortices. The electrode will be bonded to a Blackrock Neuroport Array pedestal, which can be connected to the NeuroPort System in order to process and record neural activity in real time. Using this neural interface, we will condition the patient to be able to control a wearable hand robotic exoskeleton, communicate with a computer system for typing and perform speech output tasks. We will utilize optimal neural plasticity mechanisms, a novel decoder framework, and advanced language modeling during BCI conditioning.<br><br>Hypothesis: The underlying hypothesis is that ECoG recordings will allow severely paralyzed individuals to skillfully control complex neuroprosthetic devices for movement and communication. A closely related hypothesis is that the well-known stability of ECoG signals will allow us to maximally engage neural mechanism of plasticity and thereby optimize long-term skilled acquisition.
**Patient Population**	Study subjects will be adults with severe motor impairment secondary to a neurological disorder.

Inclusion Criteria

- Age > 21
- Limited ability to use upper limbs, based on neurological examination, due to stroke, amyotrophic lateral sclerosis (ALS), multiple sclerosis, cervical spinal cord injury, brainstem stroke, muscular dystrophy, myopathy or severe neuropathy
- Disability, defined by a 4 or greater score on the Modified Rankin Scale, must be severe enough to cause loss of independence and inability to perform activities of daily living.
- If stroke or spinal cord injury, at least 1 year has passed since onset of symptoms
- Must live within a two-hour drive of UCSF

Exclusion Criteria

- Pregnancy or breastfeeding
- Inability to understand and/or read English
- Inability to give consent
- Dementia, based on history, physical exam, and MMSE
- Active depression (BDI > 20) or other psychiatric illness (active general anxiety disorder, schizophrenia, bipolar disorder, obsessive-compulsive disorder (OCD), or personality disorders (e.g. multiple personality disorder, borderline personality disorder, etc.)
- History of suicide attempt or suicidal ideation
- History of substance abuse
- Co-morbidities including ongoing anticoagulation, uncontrolled hypertension, cancer, or major organ system failure
- Inability to comply with study follow-up visits
- Any prior intracranial surgery
- History of seizures
- Immunocompromised
- Has an active infection
- Has a CSF drainage system or an active CSF leak
- Requires diathermy, electroconvulsive therapy (ECT), or transcranial magnetic stimulation (TMS) to treat a chronic condition
- Has an implanted electronic device such as a neurostimulator, cardiac pacemaker/defibrillator or medication pump
- Allergies or known hypersensitivity to materials in the Blackrock NeuroPort Array pedestal (i.e. silicone, titanium) or the PMT Subdural Cortical Electrode (silicone, platinum iridium, nichrome)

**Sample Size**	8 patients
**Efficacy Assessments**	Primary Endpoints: Feasibility of control of a wearable exoskeleton device and a communication interface.
**Safety Assessments**	• Physical examination at all study visits<br>• Perform functional test of the PMT/Blackrock combined neural interface system prior to implantation<br>• Surgical/ or nonsurgical protocol-defined adverse events recorded on adverse events case report forms and use of protocol-defined procedures for adverse event management<br>• Assessment of suicidality using Columbia Suicide Severity Rating Scale, and assessment of changes using the Beck Depression and Anxiety Inventories, at monthly intervals.

1.0 Patient Eligibility

Study subjects will be adults with severe motor impairment secondary to a neurological disorder.

Inclusion Criteria

- Age > 21
- Limited ability to use upper limbs, based on neurological examination, due to stroke, amyotrophic lateral sclerosis (ALS), multiple sclerosis, cervical spinal cord injury, brainstem stroke, muscular dystrophy, myopathy or severe neuropathy
- Disability, defined by a 4 or greater score on the Modified Rankin Scale, must be severe enough to cause loss of independence and inability to perform activities of daily living.
- If stroke or spinal cord injury, at least 1 year has passed since onset of symptoms
- Must live within a two-hour drive of UCSF

Exclusion Criteria

- Pregnancy or breastfeeding
- Inability to understand and/or read English
- Inability to give consent
- Dementia, based on history, physical exam, and MMSE
- Active depression (BDI > 20) or other psychiatric illness (active general anxiety disorder, schizophrenia, bipolar disorder, obsessive-compulsive disorder (OCD), or personality disorders (e.g. multiple personality disorder, borderline personality disorder, etc.)
- History of suicide attempt or suicidal ideation
- History of substance abuse
- Co-morbidities including ongoing anticoagulation, uncontrolled hypertension, cancer, or major organ system failure
- Inability to comply with study follow-up visits
- Any prior intracranial surgery
- History of seizures
- Immunocompromised
- Has an active infection
- Has a CSF drainage system or an active CSF leak
- Requires diathermy, electroconvulsive therapy (ECT), or transcranial magnetic stimulation (TMS) to treat a chronic condition
- Has an implanted electronic device such as a neurostimulator, cardiac pacemaker/defibrillator or medication pump
- Allergies or known hypersensitivity to materials in the Blackrock NeuroPort Array pedestal (i.e. silicone, titanium) or the PMT Subdural Cortical Electrode (silicone, platinum iridium, nichrome)

2.0 Study Device(s)

Cleared for same indication with off-label use of device for at least 1 year:

- Blackrock NeuroPort Array, PN 6248
- Blackrock NeuroPort System, PN 5416
- PMT Subdural Cortical Electrodes, Model #2110TX-128-005

3.0 Study Procedure

Study Duration: The duration of this pilot study will be 6 years. We expect that all 8 patients will be recruited in the first 4-5 years, and data collection, device development and analysis will be completed in 6 years.

Patient recruitment and clinical characterization: Patients with motor impairments secondary to neurological disorders will be recruited from clinics specializing in the treatment of stroke, ALS, and general neurological disorders, at UCSF and the San Francisco VA Medical Center.

Enrollment procedures: Prior to enrollment into the study, an informal phone interview to schedule an office-based evaluation will take place, followed by three outpatient screening visits. During the first outpatient visit we will describe the trial in detail and answer all questions. Should the participant choose to continue, we will schedule another visit to conduct a physical exam and to perform screening labs to determine eligibility. During this visit we will screen for eligibility by 1) acquiring patient demographics, 2) reviewing medical history and measuring vital signs, 3) ensuring patients are not currently pregnant or plan to become pregnant, 4) obtaining a list of current medications being taken, 5) ensuring MMSE scores are within a reasonable range (≥18, also accounting for motor difficulties with taking the test), 6) obtain baseline patient-rated and investigator rated clinical global impression scales, 7) assess baseline health status with SF-36 Health Survey, 8) assess current depression and anxiety states with Beck Depression and Anxiety inventories (BDI and BAI, respectively), 9) determine suicidal ideation risk is minimal with Columbia Suicide Severity Rating Scale (C-SSRS) 10) perform a baseline neurological physical exam and 11) determine the disability rating using the modified Rankin Scale. An MRI and CT of the brain will also be obtained for future surgical planning and to further determine eligibility. Moreover, a ECG and chest x-ray will also be obtained. We will then schedule a third follow-up visit to review this data and to answer remaining questions.

Pre-operative care: Subjects will be administered Kefzol 2g IV within 60 minutes prior to surgical incision, and continued for 24 hours post-operatively. In case of allergy to Kefzol, Vancomycin 1gm IV will be used.

Surgery: After obtaining informed consent, subjects will undergo brief general anesthesia (typically ~ 3-4 hours) for the surgical procedure. This may be either IV or inhaled anesthetics. Patient will be given IV antibiotics prior to incision, and re-dosed if required. Induction and wake-up will take place in the operating room, and patients will be monitored in the post-anesthesia care unit (PACU) after surgery for 2-3 hours during the peri-operative period. Subject will then continue to recover on the surgical ward for 2 days.

Subjects will undergo surgical placement of an ECoG array over the nondominant (usually right-sided) sensorimotor and speech cortices. The basic operative procedure is similar to what is commonly performed for non-penetrating subdural grid placement in patients with intractable epilepsy, which is well tolerated and has few complications [35]. This surgery will be smaller in exposure given that coverage will be limited to the regions of interest, and

not broadly applied as used for epilepsy localization. Standard procedures for craniotomy at the surgical site will be followed. Briefly, a 5-6 cm curvilinear incision will be made over the anatomic hand representation of the cortex (i.e."hand knob") which is located 3 cm lateral to the midline. Localization will be confirmed using intraoperative Brainlab stereotactic neuronavigation. A craniotomy will then be performed, exposing the dural surface. A wide slit in the dura will be opened.

After identifying the location for the electrode grid implant, the position for the pedestal connector is determined on the adjacent or contra-lateral skull surface and marked. A separate 2 cm scalp incision will be made for the pedestal connector. Holes will be drilled for connector placement and the connector will be secured to the skull with 8 small titanium screws. Once the connector is placed, the electrodes and wire bundle are gently manipulated to position the electrodes so that they are resting on the cortical surface over the area of interest. The nonpenetrating ECoG microarray will be sutured to the dura to secure its position. After successful placement of the electrode grid, the dura will be sutured closed in a watertight fashion and the bone flap will be replaced and secured in place with a standard titanium cranial fixation plates and screws.

The surgical site will be irrigated with antibiotic lactated ringers solution. The fascia and skin will be closed with absorbable sutures over the covered craniotomy with a slit accommodating the passage of the connector. The wound will be dressed with a sealed surgical bandage. The expected blood loss for this procedure is 50 cc. The expected operative time is 3-6 hours.

The ECoG electrode array is manufactured by PMT Corporation, and is already FDA-cleared for temporary (<30 days) clinical monitoring of neural signals (K082474). The PMT Subdural Cortical Electrodes are a chronically implantable array containing 128 electrodes, with an electrode spacing of 4 mm, capable of recording from areas of the central nervous system for extended periods of time. The electrode contacts are composed of medical grade platinum iridium, and embedded in a thin sheet of medical grade silastic. The model number 2110 indicates a platinum iridium wire and platinum iridium contacts. Platinum iridium is a mechanically robust alloy electrode material used in commercial DBS leads (Petrossians, Whalen and Weiland 2016). The electrodes' contacts are molded into a silicone rubber matrix in a fixed pattern. This identical electrode is used routinely at our institution and others for seizure localization. In the past ten years, we have not encountered any adverse inflammatory reactions or bleeding specific to the electrode arrays itself (over 100 cases). Insulated wires extend from each electrode through a flexible silicone tube to the connector on the pedestal.

Post-Operative Care: Subjects will be recovered in the intensive care unit and observed for 24 hours before transferring to the ward. A postoperative head CT will be obtained to evaluate for any hemorrhage and to confirm the position of the array. IV antibiotics (Kefzol will used if there are no allergies) will be administered for 24 hours after surgery, followed by prophylactic antibiotics given up to suture removal plus 2 days. Patients will be switched to oral antibiotics as soon as possible. Subjects will undergo physical exam every day with monitoring of vital signs, neurological exam, and basic respiratory, cardiac, and gastrointestinal exams. In addition, the wound site will be inspected at all study visits.

Routine hygiene will consist of handwashing with soap/water and donning gloves using sterile technique when coming in contact with the percutaneous connector. The caregiver

and patient will be trained to clean the pedestal site according to specific instructions that will be given to them, to be performed at least once every seven days or as required.

An infection control protocol will be strictly followed. At early signs of infection or irritation the patient/caregiver should contact the study physician immediately. The surgical site will need to be cleaned twice or more during the day, while using extra meticulous hand hygiene. If skin erythema, edema, pain and/or warmth are present, the available drainage will be cultured and oral antibiotics will be started. Plain radiographs of the involved area, ESR, CRP, WBC, and blood cultures will be obtained. If there is no improvement within 72 hours, rapid progression of erythema, symptoms worsen, or if there are signs of systemic toxicity, parenteral antibiotics is warranted. If high fever or severe pain is present, nuchal rigidity, or progressive deterioration in level of consciousness, the participant should go to the hospital emergency room. All implanted hardware will be surgically removed if there is evidence of hardware infection.

Pain related to small craniotomies is usually self-limited. Pain scale ratings will be assessed every 4 hours and routine postoperative pain management will be used. This includes the following medications as needed: acetaminophen and/or Percocet (acetaminophen/hydrocodone). IV pain meds (morphine sulfate or dilaudid) will also be administered if needed. In our experience, most patients do not require IV analgesics beyond the first operative day for smaller craniotomies.

Study visits: As outlined above, system testing will occur through two phases. In the first phase, we will simply optimize the system; in the second phase, we will commence testing of the motor and communication neural interface systems.

In **Phase 1**, the main goal is to ensure reliable neural signal monitoring and optimization of the real-time systems. Initial study visits will occur at defined time points, similar to those normally used in clinical care. For example, we anticipate a visit at post-op day (POD) 10 and 14. During this time, we will simply monitor the neural signals and briefly test the real-time communication with the computer interface. We anticipate additional visits 1-3x/week, based on the patient's availability and preference, to continue to monitor signal stability and check for wound healing. During this phase, we will also offer in home testing of the system to minimize the burden. While the equipment may be kept at the subject's home, testing will only occur when study personnel are present. The total time period of Phase 1 will be approximately 1 months, the exact time will be customized for each patient.

In **Phase 2**, we will test feasibility for both neuroprosthetic control and for decoding speech from neural activity. We will begin to perform experimental testing with the system for control of a custom wearable hand exoskeleton robot that can be classified as a non-significant risk device. As shown in panel A, the exoskeleton system consists of a table top frame that allows x,y,z movement of the arm/hand (i.e. supported by a brace attached to the platform mount). Movements are limited to the natural workspace of each subject. The hand exoskeleton (panel B) will be mounted using a readily releasable magnetic mount. The hand system aims to allow control of the fingers and thumb using a motorized cable system attached to motors. This system will allow us to test restoration of reach to grasp functions in our subjects. As outlined in the Risk Analysis (Appendix D), we have multiple safety features to ensure that there is minimal risk for injury during the embodiment phase (i.e. the subjects interact with the exoskeleton).

Throughout this period, neural signals will be recorded and analyzed, and tasks will be performed toward the development, assessment, and improvement of the neural interface system. We will assess quality of performance using kinematic parameters while performing required tasks. We will analyze stability of neural recordings and performance. To measure stability of the neural representation we will analyze the neural correlates of imagined movements. We will also examine the stability of neural correlates on neuroprosthetic exoskeleton movements (e.g. spectral content, timing, spatial recruitment). We will assess changes in the spatial correlation scales and other redundancy measures during learning and stable performance. We anticipate that testing will be conducted in the outpatient office or home setting based on the patient's preference and needs.

In this phase, we will also continue to perform experimental testing with the system for control of a virtual communicating interface. Throughout this period, neural signals will be recorded and analyzed, and tasks will be performed toward the development, assessment, and improvement of the neural interface system. We will assess the ability to control a computer communication interface. To measure stability of the neural representation we will analyze the neural correlates of imagined speech. We will also examine the stability of neural correlates on a neuroprosthetic communication device (e.g. spectral content, timing, spatial recruitment).

Neural activity monitoring: Continuous neural signal data will be acquired from the 128-channel implanted PMT Subdural Cortical Electrodes and processed with the NeuroPort System hardware by Blackrock Microsystems. Broadly, the neural data will consist of ECoG neural activity from neurons in the vicinity of each recording electrode. The neural data will be actively monitored and recorded via the graphical user interface associated with the NeuroPort hardware commercially available via Blackrock. Data read and download are non-invasive and will be performed with the patient comfortably rested.

The NeuroPort System has been successfully deployed in monitoring neural activity in patients with motor control disorders (e.g. (Hochberg et al. 2012a, Pandarinath et al. 2015)). Blackrock Microsystems provides commercial software that allows real-time filtering, recording, and visualization of acquired neural data and also allows interfacing with other programming languages such as MATLAB (Mathworks, MA). Together, this allows the ability to create custom software such as communication device based on neural spiking activity, detailed further in the following section. Overall, the mix of commercial and custom software will allow for real-time signal processing, synchronization and control of the peripheral communication device, with parallel data streams to store data for offline analyses.

Online BMI control of a Wearable Hand Robotic Exoskeleton Device:
ECoG signals will be filtered and processed in real-time using a customized portable multi-channel neurophysiology workstation NeuroPort Biopotential Signal Processing System. We will bandpass each channel into multiple bands. Past experiments, including our own, suggest that movement related information is encoded in these bands.

Initialization Phase. We will use an adaptive filter to create a 'decoder' that maps neural activity to movement of the wearable exoskeleton device. Recent experiments suggest that such a filter can rapidly allow control of neuroprosthetic devices. We will train the filter

using ‘imagined movements’. As paretic/paralyzed patients will not have access to normal overt movement related neural signals, we will use the neural basis of imagined movements for training. We anticipate that during the initial training phase, the filter will establish a set of weights between the object and neural signals. During this training phase, patients will observe a computer cursor on a screen. In order to compare visual versus visual + proprioceptive/tactile feedback signals during decoder conditioning, we will use the exoskeleton setup for decoder conditioning. The arm and hand will move in a stereotyped fashion while the subjects are instructed to ‘imagine’ actively tracking its path. Of note, we have experience with similar control of an exoskeleton system ((Ganguly et al. 2011, Ganguly et al. 2009)). As prior and as outlined below, the current system is developed by UC Berkeley. They have long-standing experience with kinematic monitoring, limb dynamics and exoskeleton development (Matthew et al. 2015, Matthew et al. 2016, Oskarsson et al. 2016).

Training Phase. Subjects will be allowed to practice tasks associated with arms/hand and object manipulation. The position of the exoskeleton end effector coordinates (x, y, z) will be under direct neural control. Preliminary experiments with an exoskeleton system showed that end effector control (position and orientation of the wrist) was more intuitive and efficient then position control of individual joints. For this initial phase, the motion will be restricted to a 2-dimensional plane for reaching and grasping objects. There are currently 9 tasks which involve interactions with both static and dynamic environments with various fixtures. We will first limit movements to a 2D environment. The additional degree of freedom involving grasp will be included based on proficiency. In addition, given that ‘motivation’ and reward are known to influence the overall learning process, a gamed-based training environment with specific goals and scoring systems were developed to engage the subject intellectually and to provide additional enrichment during the training phase.

Testing Phase. To assess robustness and stability of control over days we will assess performance characteristics in three tasks. A) Standard center-out task where subjects have to move to the center, engage a grasp, then move to a target and disengage the grasp to release of an object such as a ball. The workspace will be at 95% of the patient’s natural reach. Target size will be kept at 5 cm. B) Reaching from a randomized starting and end position in the workspace of Task A. C) Task B except with the need to plan around obstacles that are placed in the direct path.

Online BMI control: Also during phase 2, neural activity will be used to control a real-time communication device using state of the art closed-loop decoders based on rapid changes in neural activity (Shanechi, Orsborn and Carmena 2016). The main advantage of such a decoder lies in its enhanced ability in discriminating user intent and its speed of operating at every event. Such decoders operate at much higher speeds (typically at 200Hz) over previously developed decoders that rely on averaging neural activity (typically at 10Hz). In addition, we will compare this decoder to more standard decoders (e.g. the Weiner Filter, the LMS filter, the Kalman filter and variants). As documented below, the main outcome measure will be the rate of communication using these approaches.

A virtual communication effector will be presented on a computer screen, custom written in the MATLAB programming environment. The interface between MATLAB and the NeuroPort System will be via commercial software provided by Blackrock Microsystems. The novel decoder will map neural activity to the communication interface. A language processing engine will be concurrently running in the background to model and predict the words and sentences. The following metrics will be utilized to measure performance of the decoder and communication device: selections per minute, accuracy, correct characters per minute (Bacher et al. 2015b) and the bitrate, an information theoretic approach to relate accuracy, time of task completion and complexity (Nuyujukian et al. 2014b, Thompson et al. 2014a). The performance of the novel decoder based communication device will be compared to traditional state space filtering decoders such as the Kalman Filter that has been previously successfully deployed in similar BMI paradigms (Gilja et al. 2012, Bacher et al. 2015b).

Development life cycle of the BMI motor control and communication software:

a) Scope: The intended use of the decoder is to allow the patient to achieve control of external devices and thereby select characters and letters to form sentences, as well as control a wearable hand exoskeleton. As such the operation of the BCI is therefore dependent on the functionality of the software.

b) Platform: The software will be developed on MATLAB (The Mathworks, MA) and MATLAB supported C/C++ complied programs (MEX files) and will be running on the data acquisition PC that interfaces with the Blackrock NeuroPort Array pedestal connector. We will use the software libraries that are part of the Blackrock Neuroport system to stream neural data into MATLAB in real-time.

c) Inputs and outputs: The inputs to the software will be the neural signals from the PMT Subdural Cortical Electrodes grid and the output of the software will consist of user controlled (via the user's neural signals) effector position, selections of characters, letters and numbers for communication purposes, in addition to control of a movements of a wearable hand exoskeleton.

d) Components: There are four distinct aspects of the software, three that operate 'behind the scenes' and one that serves as a Graphical User Interface for display. First, is the decoder itself that translates neural signals into user intentions. Second, is another parallel decoder that serves to discriminate when the patient has made a particular selection (or e.g. grasping actions). Third, is the software engine, that keeps track of the current selections made and generates a list of probable options using a statistical model of movement direction and language (Nadkarni, Ohno-Machado and Chapman 2011b). The fourth and final aspect of the decoder is the Graphical User Interface (GUI) that displays and controls the real-time position of end effector.

e) Safety: The software provides only visual feedback to the user and does not directly interface with the neural signal data acquisition process. The software only serves to allow the use to control the communication interface and the exoskeleton position.

f) Planning phase: In the planning phase, we will identify off-the-shelf components (such as language processing engines) and will aim to further refine and customize it in-house concurrently with the decoders and GUI.

g) Development phase: In the development phase, all components will be developed in parallel as discrete subunits of the overall functional system. A code repository will be maintained to keep track of the life cycle versions and code will be commented wherever

appropriate. During the development phase, debugging will be performed at every iteration and documented. The documentation and code will be maintained on secure hard drives.
h) Testing phase: There are two aspects of this phase. One is the performance testing of each of the four individual components and the other is the testing of the entire software. In lieu of actual neural signals, simulated neural signals will be delivered as input, with a known mapping between the input and output state as the ground truth is known a priori. This will allow testing the performance of the decoders (accuracy in estimated positions). The testing of the GUI and the software engine will be performed independently of the decoders by manually controlling the position of the effector. The testing of the GUI and the software engine will be assessed by the stability and reliability in updating effector position and exoskeleton movements, in the turnaround time of displaying the list of predictive words and actions based on current selections. The overall system testing will employ a combination of simulated neural signals and manual position control to assess the ability of the software in allowing a user to communicate and control a hand neuroprosthetic.
i) Error handling: Code will be written to specifically monitor potential sources of errors in real-time decoding due to either noise in neural signals or decoder weight drift that would necessitate recalibration and resetting of the GUI and software engine.
j) Software validation: The validation and formal design review for the overall software will be performed by members of the PI's laboratory not involved with the development and testing of the software prior to software deployment.
k) Resolution and maintenance: Active documentation and daily logs will be noted to keep track of the performance of the software and address issues such as version control, robustness and immediate resolution of unforeseen errors in the software.

Progression of phases: We anticipate phase 1 will last approximately 1month, but will vary based on each subject. Phase 2 will last at a minimum 10-11 months, a total amount of time of at least 1 year after PMT Subdural Cortical Electrodes implantation and neural interface monitoring and testing, a timeframe which has been performed or exceeded without adverse effects by previous studies using the PMT Subdural Cortical Electrodes and Blackrock Microsystems NeuroPort Array pedestal and NeuroPort System. In this study, recordings were made over 666 days in a non-human primate with no adverse events related to the implanted devices (Degenhart et al. 2016). The ECoG array is identical to that used for subdural grid placement in patients with intractable epilepsy, which is well tolerated and has few complications (Chang et al. 2010).

Activation of the brain recording function: All data collection in the study visits will be initiated by the study staff.

Conclusion of study: For each enrolled subject, if there have been no serious adverse events, we will present the option to continue with the study at the end of a 1-year period. If the subject chooses, we will continue with testing for another year. We will formally present this option every year for a period of 5 years. Notably, the subject will be reminded that he/she will have the option for surgical removal of the device at any point.

Removal of the ECoG grid and Connector pedestal: At the conclusion of the study, or earlier if medically indicated, the subdural cortical electrodes and connector pedestal will be

surgically removed. The skin incision and bone flap will be reopened and the electrode will be removed and discarded. The dura will be sutured tightly. The galea and scalp will be sutured closed. The expected blood loss is minimal (less than 10 cc), and the expected operative time is 30 minutes.

4.0 Clinical Measurements

Primary

This is a pilot study to test feasibility in eight subjects.

For BCI motor control, we will use outcome measures that are frequently used in preclinical studies of neuroprostheses (e.g. accuracy and reliability of cursor and limb control). As outlined below, the primary goal will be to gather statistics regarding the best achievable control using ECoG signals and state-of-the-art methods to allow motor neuroprosthetic control. For each of the parameters below we aim to describe the statistics as the mean and the variance (Bacher et al. 2015a, Nadkarni et al. 2011a, Nuyujukian et al. 2014a, Shanechi et al. 2016, Thompson et al. 2014b). Ultimately, we will compare these values to a wealth of published data regarding movements in able-bodied subjects, e.g.(Bacher et al. 2015a).

1. *Quality of performance* will be assessed using kinematic parameters while performing the required tasks. We will assess stability of the trajectories in the tasks. We will then assess ability for generalization from any region in the workspace to another random point. Position errors from the selected trajectory of the task as well as velocity and acceleration will be studied in both joint space and the end effector space.

2. *Recording and Performance Stability.* We will use previously established metrics to analyze stability of neural recordings (Shanechi et al. 2016, Thompson et al. 2014b) and performance. To measure stability of the neural representation we will analyze the neural correlates of daily imagined movements. We will also examine the stability of the neural correlates of neuroprosthetic movements (e.g. spectral content, timing, spatial recruitment).

3. *Spatial Scale.* An important question for ECoG recordings is the optimal spatial scale for the electrode grid. This has implications for maximizing the amount of information that can be obtained from the recording setup but also for defining the design specifications of implantable electronics (e.g. power requirements could vary greatly depending on the spatial and temporal resolution of the neural data required). We will look at changes in the spatial correlation scales and other redundancy measures during learning and stable performance.

The following metrics will further be utilized to measure performance of the decoder and communication device: selections per minute, accuracy, correct characters per minute (Bacher et al. 2015b) and the bitrate, an information theoretic approach to relate accuracy, time of task completion and complexity (Nuyujukian et al. 2014b, Thompson et al. 2014a). Physiological measurements related to neural activity will include statistical assessments of z-scored activity from single electrodes as well as population dynamics. Physiological measurements related to oscillatory activity will include: wide spectrum power-spectral

analysis as well as using specific frequency domain for mean log power (i.e. in the delta, theta, alpha, beta, gamma bands), coupling between the phase of low frequency rhythms and broadband gamma amplitude (phase-amplitude coupling, abbreviated PAC) (Canolty et al. 2006, Miller et al. 2010, Tort et al.).

Clinical measures and physiological measurements will be collected and recorded by members of the research and clinical team.

5.0 Data Management

All clinical and physiological data will be stored in encrypted and password-protected computers in the PI's laboratory that is always locked. If the Neuroport System is stored in the participant's residence, all research data will be maintained in accordance with UCSF standard encryption policy. In publications or presentations of the data, data will be grouped by case number in chronological order with no name identification. All patients will be asked to sign a separate consent for audio-video recording. When presenting videotape data at scientific conferences, we will utilize only videos from patients who have consented to have their videos shown. De-identified electrophysiological data may be shared with other researchers at other institutions.

6.0 Statistical Methods and Data Analysis

To assess the performance of the decoder, analyses will be performed on the kinematics associated with control, such as time to reach a target, trajectory curvature etc., in conjunction with measures associated with the communication device such as the bit rate, accuracy, characters per minute. The statistical reliability of the decoder will be assessed by non-parametric data permutation wherein the learned mapping between neural activity and the effector position will be artificially broken down and shuffled. Field potential data will be analyzed using wide spectrum power-spectral analysis as well as using specific frequency domain for mean log power (i.e. in the delta, theta, alpha, beta, gamma bands). We will also examine coherence and cross-frequency coupling between the channels (Canolty et al. 2006, Miller et al. 2010, Tort et al.). Using a repeated measures ANOVA statistical analysis, summary statistics for power in relevant frequency bands, control related power changes, and indices of phase-amplitude coupling will be compared at different time-points of control. Additionally, bootstrap statistical tests and general linear mixed models can be utilized to investigate potential statistical effects, given the small sample size. Mean, median, variance and median absolute deviation describing the statistics of each of the measured outcome (such as accuracy, effector position control) will be recorded for each subject.

Sample size calculation: This is a pilot study to assess the feasibility of an ECoG based implantable BCI device in patients with motor control disorders using intracranial recordings, a communication interface and a wearable hand exoskeleton. The collected pilot data will aid in determining the feasibility, reliability and future directions of the brain machine interface for communication and motor control. In addition, the pilot data will be used to formulate more detailed hypothesis on neural plasticity and BMI control in humans. As such, there is no formal sample size requirement for this pilot study.

Criteria for study success that would justify a larger subsequent trial:

1) Ability to use ECoG-based neural activity to control a neuroprosthetic device and communication interface.
2) No permanent serious adverse events occur (such as trauma with long-term motor deficit).
3) Benefits to the patient in regaining a sense of control over the ability to exert motor control and communicate in an efficient manner.

7.0 Regulatory Requirements

Prior to the start of the study, the following documents will be collected and filed:

- Signed protocol signature page
- Curriculum vitae of the PIs and Sub-investigators, updated within 2 years
- Current medical licenses for the PIs and all Sub-investigators
- Financial disclosure form signed by the PIs and all Sub-investigators
- Copy of the IRB approval letter for the study and the IRB Membership List
- Investigator Agreement

Investigator Obligations

Redacted will be responsible for ensuring that all study site personnel, adhere to all FDA regulations and guidelines regarding clinical trials, including guidelines for GCP (including the archiving of essential documents), both during and after study completion. Additionally, they are responsible for the subject's compliance to the study protocol.

All information obtained during the conduct of the study with respect to the patients' state of health will be regarded as confidential. This is detailed in the written information provided to the patient. An agreement for disclosure of any such information will be obtained in writing and will be signed by the patient.

Informed Consent

The investigators will obtain and document informed consent for each patient screened for this study. All patients will be informed in writing of the nature of the protocol and investigational therapy, its possible hazards, and their right to withdraw at any time, and will sign a form indicating their consent to participate prior to the initiation of study procedures.

Institutional Review Board

This protocol and relevant supporting data are to be submitted to the appropriate IRB for review and approval before the study can be initiated (UCSF, Human Research Protection Program, 3333 California Street, Suite 315, San Francisco, CA, 94118, FWA#00000068; IRB Registration 00000229, Lisa Denney, HRRP Director). Amendments to the protocol will also be submitted to the IRB prior to implementation of the change. The PIs are responsible for informing the IRB of the progress of the study and for obtaining annual IRB renewal. The IRB must be informed at the time of completion of the study and should be provided with a summary of the results of the study by the PIs. The PIs must notify the IRB in writing of any SAE or any unexpected AE according to ICH guidelines.

Data safety monitory board (DSMB) and safety monitoring plan

Treatment emergent adverse events that are assessed by the principal investigators as possibly, probably, or definitely related to surgical implantation or chronic cortical recording AND are unexpected or meet seriousness criteria (death, immediately life threatening, hospitalization >24 hours, persistent or significant disability, or significant

intervention required to prevent one of the previously-stated outcomes) will be recorded and reported to the IRB, device manufacturer and the FDA via the MedWatch online voluntary reporting form within 10 working days of the study team's knowledge of the event.

All such events will also be reported to the data safety monitor board (DSMB), led by a neurosurgeon at our home institution, who does not have direct involvement in this study but who has expertise in implantable devices, pain management and neurosurgery. The DSMB will meet regularly to review data related to the clinical trial, provide guidance and feedback, and review any adverse event reports. Treatment-related adverse events assessed as definitely, probably, or possibly related to study procedures and either serious or unexpected, noted by any study personnel will be reported within 10 working days of their knowledge of the event to the DSMB. The DSMB will then advise the PI on potential changes in procedures to improve safety. The safety endpoint will consist of all adverse events.

Throughout the clinical trial, should a serious adverse event occur that is assessed to be surgery related or not, or related to the presence of the electrode system, such as infection, the device will be removed and the study halted for the patient. Removal will be accomplished by re-opening the original incisions, temporary removal of the bone, and removal of the PMT Subdural Cortical Electrodes from the brain and NeuroPort Array pedestal from the skull. The dura will be re-sewn together and the bone fixed again with titanium screws

Furthermore, if there is a serious surgical or non-surgical adverse event, or with the onset of suicidality, the study will be halted for the patient.

If two patients meet one or more of these criteria (i. serious surgical or nonsurgery-related adverse event, or ii. onset of suicidality), the study will be halted until information is reviewed by the DSMB and FDA.

8.0 References

Ajiboye, A. B., J. D. Simeral, J. P. Donoghue, L. R. Hochberg & R. F. Kirsch (2012) Prediction of imagined single-joint movements in a person with high-level tetraplegia. *IEEE Trans Biomed Eng,* 59, 2755-65.

Anderson, K. D. (2004) Targeting recovery: priorities of the spinal cord-injured population. *J Neurotrauma,* 21, 1371-83.

Bacher, D., B. Jarosiewicz, N. Y. Masse, S. D. Stavisky, J. D. Simeral, K. Newell, E. M. Oakley, S. S. Cash, G. Friehs & L. R. Hochberg (2015a) Neural Point-and-Click Communication by a Person With Incomplete Locked-In Syndrome. *Neurorehabil Neural Repair,* 29, 462-71.

Bacher, D., B. Jarosiewicz, N. Y. Masse, S. D. Stavisky, J. D. Simeral, K. Newell, E. M. Oakley, S. S. Cash, G. Friehs & L. R. Hochberg (2015b) Neural point-and-click communication by a person with incomplete locked-in syndrome. *Neurorehabilitation and neural repair,* 29, 462-471.

Bensmaia, S. J. & L. E. Miller (2014) Restoring sensorimotor function through intracortical interfaces: progress and looming challenges. *Nat Rev Neurosci,* 15, 313-25.

Birbaumer, N., N. Ghanayim, T. Hinterberger, I. Iversen, B. Kotchoubey, A. Kubler, J. Perelmouter, E. Taub & H. Flor (1999) A spelling device for the paralysed. *Nature,* 398, 297-8.

Bouchard, K. E., N. Mesgarani, K. Johnson & E. F. Chang (2013) Functional organization of human sensorimotor cortex for speech articulation. *Nature,* 495, 327-32.

Bouton, C. E., A. Shaikhouni, N. V. Annetta, M. A. Bockbrader, D. A. Friedenberg, D. M. Nielson, G. Sharma, P. B. Sederberg, B. C. Glenn, W. J. Mysiw, A. G. Morgan, M. Deogaonkar & A. R. Rezai (2016) Restoring cortical control of functional movement in a human with quadriplegia. *Nature,* 533, 247-50.

Canolty, R. T., E. Edwards, S. S. Dalal, M. Soltani, S. S. Nagarajan, H. E. Kirsch, M. S. Berger, N. M. Barbaro & R. T. Knight (2006) High gamma power is phase-locked to theta oscillations in human neocortex. *Science,* 313, 1626-8.

Carmena, J. M., M. A. Lebedev, R. E. Crist, J. E. O'Doherty, D. M. Santucci, D. F. Dimitrov, P. G. Patil, C. S. Henriquez & M. A. Nicolelis (2003) Learning to control a brain-machine interface for reaching and grasping by primates. *PLoS Biol,* 1, E42.

Chang, E. F., J. W. Rieger, K. Johnson, M. S. Berger, N. M. Barbaro & R. T. Knight (2010) Categorical speech representation in human superior temporal gyrus. *Nat Neurosci,* 13, 1428-32.

Chao, Z. C., Y. Nagasaka & N. Fujii (2010) Long-term asynchronous decoding of arm motion using electrocorticographic signals in monkeys. *Front Neuroengineering,* 3, 3.

Chestek, C. A., V. Gilja, P. Nuyujukian, R. J. Kier, F. Solzbacher, S. I. Ryu, R. R. Harrison & K. V. Shenoy (2009) HermesC: Low-Power Wireless Neural Recording System for Freely Moving Primates. *Ieee Transactions on Neural Systems and Rehabilitation Engineering,* 17, 330-338.

Churchland, M. M., J. P. Cunningham, M. T. Kaufman, J. D. Foster, P. Nuyujukian, S. I. Ryu & K. V. Shenoy (2012) Neural population dynamics during reaching. *Nature,* 487, 51-6.

Collinger, J. L., S. Foldes, T. M. Bruns, B. Wodlinger, R. Gaunt & D. J. Weber (2013) Neuroprosthetic technology for individuals with spinal cord injury. *J Spinal Cord Med,* 36, 258-72.

Degenhart, A. D., J. Eles, R. Dum, J. L. Mischel, I. Smalianchuk, B. Endler, R. C. Ashmore, E. C. Tyler-Kabara, N. G. Hatsopoulos, W. Wang, A. P. Batista & X. T. Cui (2016) Histological evaluation of a chronically-implanted electrocorticographic electrode grid in a non-human primate. *J Neural Eng,* 13, 046019.

Ganguly, K. & J. M. Carmena (2009) Emergence of a stable cortical map for neuroprosthetic control. *PLoS Biol,* 7, e1000153.

Ganguly, K., D. F. Dimitrov, J. D. Wallis & J. M. Carmena (2011) Reversible large-scale modification of cortical networks during neuroprosthetic control. *Nat Neurosci,* 14, 662-7.

Ganguly, K., L. Secundo, G. Ranade, A. Orsborn, E. F. Chang, D. F. Dimitrov, J. D. Wallis, N. M. Barbaro, R. T. Knight & J. M. Carmena (2009) Cortical representation of ipsilateral arm movements in monkey and man. *J Neurosci,* 29, 12948-56.

Gilja, V., C. A. Chestek, I. Diester, J. M. Henderson, K. Deisseroth & K. V. Shenoy (2011) Challenges and opportunities for next-generation intracortically based neural prostheses. *IEEE Trans Biomed Eng,* 58, 1891-9.

Gilja, V., P. Nuyujukian, C. A. Chestek, J. P. Cunningham, M. Y. Byron, J. M. Fan, M. M. Churchland, M. T. Kaufman, J. C. Kao & S. I. Ryu (2012) A high-performance neural prosthesis enabled by control algorithm design. *Nature neuroscience,* 15, 1752-1757.

Hochberg, L. R., D. Bacher, B. Jarosiewicz, N. Y. Masse, J. D. Simeral, J. Vogel, S. Haddadin, J. Liu, S. S. Cash & P. van der Smagt (2012a) Reach and grasp by people with tetraplegia using a neurally controlled robotic arm. *Nature,* 485, 372-375.

Hochberg, L. R., D. Bacher, B. Jarosiewicz, N. Y. Masse, J. D. Simeral, J. Vogel, S. Haddadin, J. Liu, S. S. Cash, P. van der Smagt & J. P. Donoghue (2012b) Reach and grasp by people with tetraplegia using a neurally controlled robotic arm. *Nature,* 485, 372-5.

Hochberg, L. R., M. D. Serruya, G. M. Friehs, J. A. Mukand, M. Saleh, A. H. Caplan, A. Branner, D. Chen, R. D. Penn & J. P. Donoghue (2006) Neuronal ensemble control of prosthetic devices by a human with tetraplegia. *Nature,* 442, 164-71.

Homer, M. L., A. V. Nurmikko, J. P. Donoghue & L. R. Hochberg (2013) Sensors and decoding for intracortical brain computer interfaces. *Annu Rev Biomed Eng,* 15, 383-405.

Huggins, J. E., P. A. Wren & K. L. Gruis (2011) What would brain-computer interface users want? Opinions and priorities of potential users with amyotrophic lateral sclerosis. *Amyotroph Lateral Scler,* 12, 318-24.

Kennedy, P. R. (1994) 'Locked-in' patients. *Neurology,* 44, 366-7.

Kennedy, P. R. & R. A. Bakay (1998) Restoration of neural output from a paralyzed patient by a direct brain connection. *Neuroreport,* 9, 1707-11.

Kim, S. P., J. D. Simeral, L. R. Hochberg, J. P. Donoghue & M. J. Black (2008a) Neural control of computer cursor velocity by decoding motor cortical spiking activity in humans with tetraplegia. *Journal of Neural Engineering,* 5, 455-76.

--- (2008b) Neural control of computer cursor velocity by decoding motor cortical spiking activity in humans with tetraplegia. *J Neural Eng,* 5, 455-76.

Kubler, A., B. Kotchoubey, J. Kaiser, J. R. Wolpaw & N. Birbaumer (2001) Brain-computer communication: unlocking the locked in. *Psychol Bull,* 127, 358-75.

Leuthardt, E. C., K. J. Miller, G. Schalk, R. P. Rao & J. G. Ojemann (2006) Electrocorticography-based brain computer interface--the Seattle experience. *IEEE Trans Neural Syst Rehabil Eng,* 14, 194-8.

Leuthardt, E. C., G. Schalk, J. Roland, A. Rouse & D. W. Moran (2009) Evolution of brain-computer interfaces: going beyond classic motor physiology. *Neurosurg Focus,* 27, E4.

Leuthardt, E. C., G. Schalk, J. R. Wolpaw, J. G. Ojemann & D. W. Moran (2004) A brain-computer interface using electrocorticographic signals in humans. *J Neural Eng,* 1, 63-71.

Matthew, R. P., E. J. Mica, W. Meinhold, J. A. Loeza, M. Tomizuka & R. Bajcsy (2015) Initial investigation into the effect of an Active/Passive exoskeleton on hammer curl performance in healthy subjects. *Conf Proc IEEE Eng Med Biol Soc,* 2015, 3607-10.

Matthew, R. P., V. Shia, G. Venture & R. Bajcsy (2016) Generating physically realistic kinematic and dynamic models from small data sets: An application for sit-to-stand actions. *Conf Proc IEEE Eng Med Biol Soc,* 2016, 2173-2178.

Miller, K. J., D. Hermes, C. J. Honey, M. Sharma, R. P. Rao, M. den Nijs, E. E. Fetz, T. J. Sejnowski, A. O. Hebb, J. G. Ojemann, S. Makeig & E. C. Leuthardt (2010) Dynamic modulation of local population activity by rhythm phase in human occipital cortex during a visual search task. *Front Hum Neurosci,* 4, 197.

Monti, M. M., A. Vanhaudenhuyse, M. R. Coleman, M. Boly, J. D. Pickard, L. Tshibanda, A. M. Owen & S. Laureys (2010) Willful modulation of brain activity in disorders of consciousness. *N Engl J Med,* 362, 579-89.

Morrell, M. J. & R. N. S. S. i. E. S. Group (2011) Responsive cortical stimulation for the treatment of medically intractable partial epilepsy. *Neurology,* 77, 1295-304.

Nadkarni, P. M., L. Ohno-Machado & W. W. Chapman (2011a) Natural language processing: an introduction. *J Am Med Inform Assoc,* 18, 544-51.

Nadkarni, P. M., L. Ohno-Machado & W. W. Chapman (2011b) Natural language processing: an introduction. *Journal of the American Medical Informatics Association,* 18, 544-551.

Nicolelis, M. A. & M. A. Lebedev (2009) Principles of neural ensemble physiology underlying the operation of brain-machine interfaces. *Nat Rev Neurosci,* 10, 530-40.

Nuyujukian, D. S., J. Voutsinas, L. Bernstein & S. S. Wang (2014a) Medication use and multiple myeloma risk in Los Angeles County. *Cancer Causes Control,* 25, 1233-7.

Nuyujukian, P., J. C. Kao, J. M. Fan, S. D. Stavisky, S. I. Ryu & K. V. Shenoy (2014b) Performance sustaining intracortical neural prostheses. *Journal of neural engineering,* 11, 066003.

Oskarsson, B., N. C. Joyce, E. De Bie, A. Nicorici, R. Bajcsy, G. Kurillo & J. J. Han (2016) Upper extremity 3-dimensional reachable workspace assessment in amyotrophic lateral sclerosis by Kinect sensor. *Muscle Nerve,* 53, 234-41.

Pandarinath, C., V. Gilja, C. H. Blabe, P. Nuyujukian, A. A. Sarma, B. L. Sorice, E. N. Eskandar, L. R. Hochberg, J. M. Henderson & K. V. Shenoy (2015) Neural population dynamics in human motor cortex during movements in people with ALS. *Elife,* 4, e07436.

Pasley, B. N., S. V. David, N. Mesgarani, A. Flinker, S. A. Shamma, N. E. Crone, R. T. Knight & E. F. Chang (2012) Reconstructing speech from human auditory cortex. *Plos Biology,* 10, e1001251.

Schalk, G., K. J. Miller, N. R. Anderson, J. A. Wilson, M. D. Smyth, J. G. Ojemann, D. W. Moran, J. R. Wolpaw & E. C. Leuthardt (2008) Two-dimensional movement control using electrocorticographic signals in humans. *J Neural Eng,* 5, 75-84.

Schwartz, A. B. (2004) Cortical neural prosthetics. *Annu Rev Neurosci,* 27, 487-507.

Schwartz, A. B., X. T. Cui, D. J. Weber & D. W. Moran (2006) Brain-controlled interfaces: movement restoration with neural prosthetics. *Neuron,* 52, 205-20.
Selzer, M. E., S. Clarke, L. G. Cohen, G. Kwakkel & R. H. Miller. 2014. *Textbook of neural repair and rehabilitation*. Cambridge: Cambridge University Press.
Shanechi, M. M., A. L. Orsborn & J. M. Carmena (2016) Robust Brain-Machine Interface Design Using Optimal Feedback Control Modeling and Adaptive Point Process Filtering. *PLoS Comput Biol,* 12, e1004730.
Shenoy, K. V. & J. M. Carmena (2014) Combining decoder design and neural adaptation in brain-machine interfaces. *Neuron,* 84, 665-80.
Simeral, J. D., S. P. Kim, M. J. Black, J. P. Donoghue & L. R. Hochberg (2011) Neural control of cursor trajectory and click by a human with tetraplegia 1000 days after implant of an intracortical microelectrode array. *J Neural Eng,* 8, 025027.
Slutzky, M. W., L. R. Jordan, E. W. Lindberg, K. E. Lindsay & L. E. Miller (2011) Decoding the rat forelimb movement direction from epidural and intracortical field potentials. *J Neural Eng,* 8, 036013.
Spataro, R., M. Ciriacono, C. Manno & V. La Bella (2014) The eye-tracking computer device for communication in amyotrophic lateral sclerosis. *Acta Neurol Scand,* 130, 40-5.
Taylor, D. M., S. I. Tillery & A. B. Schwartz (2002) Direct cortical control of 3D neuroprosthetic devices. *Science,* 296, 1829-32.
Thompson, D. E., L. R. Quitadamo, L. Mainardi, S. Gao, P.-J. Kindermans, J. D. Simeral, R. Fazel-Rezai, M. Matteucci, T. H. Falk & L. Bianchi (2014a) Performance measurement for brain–computer or brain–machine interfaces: a tutorial. *Journal of neural engineering,* 11, 035001.
Thompson, D. E., L. R. Quitadamo, L. Mainardi, K. U. Laghari, S. Gao, P. J. Kindermans, J. D. Simeral, R. Fazel-Rezai, M. Matteucci, T. H. Falk, L. Bianchi, C. A. Chestek & J. E. Huggins (2014b) Performance measurement for brain-computer or brain-machine interfaces: a tutorial. *J Neural Eng,* 11, 035001.
Tort, A. B., R. Komorowski, H. Eichenbaum & N. Kopell (2010) Measuring phase-amplitude coupling between neuronal oscillations of different frequencies. *J Neurophysiol,* 104, 1195-210.
Wolpaw, J. R., N. Birbaumer, D. J. McFarland, G. Pfurtscheller & T. M. Vaughan (2002) Brain-computer interfaces for communication and control. *Clin Neurophysiol,* 113, 767-91.

CLINICAL PROTOCOL
(updated 6/22/2020)

Title: A High-Performance ECoG-based Neural Interface for Communication and Neuroprosthetic Control

Study Sponsors/Investigators:
Redacted

Protocol Synopsis

**Title**	**A High-Performance ECoG-based Neural Interface for Communication and Neuroprosthetic Control**
**Study Phase**	Phase I
**Device(s)**	**Device Information:** Devices to be used in this study are grouped below according to FDA approval.<br><br>**Cleared for temporary (<30 days) recording and monitoring of brain electrical activity under 510k:**<br>• **NeuroPort Array, PN 6248 (K070272, K110010)**<br><br>**Seeking Special 510(k) (not yet submitted to FDA):**<br>• **Digital NeuroPort System(Modification to existing NeuroPort System - K042626)**<br><br>**Cleared for temporary (<30 day) use with recording, monitoring, and stimulation equipment for the recording, monitoring and stimulation of electrical signals on the surface of the brain under 510k:**<br>• **PMT Subdural Cortical Electrodes, Model #2110TX-128-005 (K082474)**<br><br>The Blackrock Microsystems Neuroport Array connector pedestal, a subcomponent of the Blackrock Microsystems NeuroPort Array, will be laser bonded to the PMT Subdural Cortical Electrode by Blackrock Microsystems. As documented below, this specific approach has already been tested in non-human primates (over at least an 18-month period, see below regarding published report).<br><br>**Request for off-label use of the combined investigational device for 1 year, for the equivalent indication of recording and monitoring of brain electrical activity.**
**Indication**	Adults with neurological disorders (e.g. amyotrophic lateral sclerosis/ALS, spinal cord injury, multiple sclerosis, stroke) often develop disorders of movement and communication. We aim to determine the feasibility of ECoG based brain computer interface control of complex neuroprosthetic devices.
**Sponsor Contacts**	*Redacted*
**Data Safety Monitor Board (DSMB)**	*Redacted*
**Treatment**	The Blackrock Microsystems NeuroPort Array connector pedestal, NeuroPort system, and PMT Subdural Cortical Electrodes are currently cleared for monitoring of patients for up to 30 days. Blackrock has modifications to the NeuroPort system for which they are seeking special 510(k)

	clearance. Here we to aim to use these systems combined for at least a 1-year period in subjects with neurological illness and disorders of communication to test feasibility for both neuroprosthetic control and for decoding speech from neural activity.
**Study Sites**	• *Redacted*
**Study Design**	This is a **single-center early feasibility study** of the use of an ECoG-based neural interface for testing the feasibility of using ECoG signals to control complex devices for motor and speech control in adults affected by neurological disorders of movement.<br><br>A PMT Subdural Cortical Electrode array, bonded to the Blackrock Microsystems NeuroPort Array pedestal, will be surgically placed directly on the brain surface over the motor and language cortices of subjects with disorders of motor control. After implantation of the electrode and pedestal, the Neuroport Biopotential Processing System will be connected to the Neuroport Array pedestal to monitor and record neural signals. With this ECoG-based neural interface, study patients will undergo training and assessment of their ability to control a wearable hand robotic exoskeleton and to determine if ECoG brain signals can be decoded for language communication. This will be performed in two phases.<br><br><u>Phase 1: Optimize BCI system</u><br>In Phase 1, we will optimize the entire system to reliably detect neural activity to ensure that the recorded signals are stable and free of artifacts. Moreover, we will ensure that the neural signals are converted in real-time into cursor movements. During this phase, we will primarily examine cursor based control, decoding of parameters and 'disembodied' control (i.e. the subjects' arm will not interact with the mechanical system). This phase will be conducted in the outpatient office setting and/or the patient's home environment, based on patient preference and needs. We anticipate that this phase will take approximately 1 month; however, this may be longer or shorter for each subject depending on the level of control achieved.<br><br><u>Phase 2: Testing of BCI Control</u><br>In Phase 2, we will test feasibility for both neuroprosthetic control and for decoding speech from neural activity. We will begin to perform experimental testing with the system for control of a custom wearable hand exoskeleton robot that meets criteria for a non-significant risk device. As outlined in the sections below, we have multiple safety features to ensure that there is minimal risk for injury during the embodiment phase (i.e. the subjects arm is interacting with the exoskeleton). Throughout this period, neural signals will

	be recorded and analyzed, and tasks will be performed toward the development, assessment, and improvement of the neural interface system. We will assess quality of performance using kinematic parameters while performing required tasks. We will analyze stability of neural recordings and performance. To measure stability of the neural representation we will analyze the neural correlates of imagined movements. We will also assess changes in spatial correlation scales and other redundancy measures during learning and stable performance. We anticipate that testing will be conducted in the outpatient office or home setting based on the patient's preference and needs.<br><br>In this phase, we will also continue to perform experimental testing with the system for control of a virtual communicating interface. Throughout this period, neural signals will be recorded and analyzed, and tasks will be performed toward the development, assessment, and improvement of the communication interface.
**Objectives**	In eight patients with severe disorders of movement control, we will surgically implant PMT Subdural Cortical Electrodes on the brain surface over the motor and language cortices. The electrode will be bonded to a Blackrock Neuroport Array pedestal, which can be connected to the Digital NeuroPort system in order to process and record neural activity in real time. Using this neural interface, we will condition the patient to be able to control a wearable hand robotic exoskeleton, communicate with a computer system for typing and perform speech output tasks. We will utilize optimal neural plasticity mechanisms, a novel decoder framework, and advanced language modeling during BCI conditioning.<br><br>Hypothesis: The underlying hypothesis is that ECoG recordings will allow severely paralyzed individuals to skillfully control complex neuroprosthetic devices for movement and communication. A closely related hypothesis is that the well-known stability of ECoG signals will allow us to maximally engage neural mechanism of plasticity and thereby optimize long-term skilled acquisition.
**Patient Population**	Study subjects will be adults with severe motor impairment secondary to a neurological disorder.<br><br><u>Inclusion Criteria</u><br>• Age > 21<br>• Limited ability to use upper limbs, based on neurological examination, due to stroke, amyotrophic lateral sclerosis (ALS), multiple sclerosis, cervical spinal cord injury, brainstem stroke, muscular dystrophy, myopathy or severe neuropathy<br>• Disability, defined by a 4 or greater score on the Modified

	Rankin Scale, must be severe enough to cause loss of independence and inability to perform activities of daily living.<br>• If stroke or spinal cord injury, at least 1 year has passed since onset of symptoms<br>• Must live within a two-hour drive of UCSF<br><br><u>Exclusion Criteria</u><br>• Pregnancy or breastfeeding<br>• Inability to understand and/or read English<br>• Inability to give consent<br>• Dementia, based on history, physical exam, and MMSE<br>• Active depression (BDI > 20) or other psychiatric illness (active general anxiety disorder, schizophrenia, bipolar disorder, obsessive-compulsive disorder (OCD), or personality disorders (e.g. multiple personality disorder, borderline personality disorder, etc.)<br>• History of suicide attempt or suicidal ideation<br>• History of substance abuse<br>• Co-morbidities including uncontrolled hypertension, cancer, or major organ system failure.<br>• Ongoing anticoagulation which cannot be stopped in the peri-procedural period.<br>• Inability to comply with study follow-up visits<br>• Any prior intracranial surgery<br>• History of seizures<br>• Immunocompromised<br>• Has an active infection<br>• Has a CSF drainage system or an active CSF leak<br>• Requires diathermy, electroconvulsive therapy (ECT), or transcranial magnetic stimulation (TMS) to treat a chronic condition<br>• Has an implanted electronic device such as a neurostimulator, cardiac pacemaker/defibrillator or medication pump<br>• Allergies or known hypersensitivity to materials in the Blackrock NeuroPort Array pedestal (i.e. silicone, titanium) or the PMT Subdural Cortical Electrode (silicone, platinum iridium, nichrome)
**Sample Size**	8 patients
**Efficacy Assessments**	<u>Primary Endpoints</u>: Feasibility of control of a wearable exoskeleton device and a communication interface.
**Safety Assessments**	• Physical examination at all study visits<br>• Perform functional test of the PMT/Blackrock combined neural interface system prior to implantation<br>• Surgical/ or nonsurgical protocol-defined adverse events recorded on adverse events case report forms and use of

	protocol-defined procedures for adverse event management<br>• Assessment of suicidality using Columbia Suicide Severity Rating Scale, and assessment of changes using the Beck Depression and Anxiety Inventories, at monthly intervals.

Table of Contents

Title Page

i. Protocol Synopsis

ii Table of Contents

iii Investigator Agreement / Protocol Signature Page

1. Investigational Plan

2. Patient Eligibility

3. Study Device

4. Study Procedure

5. Clinical Measurements and Procedures

6. Data Management

7. Statistical Methods and Data Analysis

8. Regulatory Requirements

9. References

Investigator Agreement / Protocol Signature Page

Redacted

1.0 Investigational Plan

We aim to conduct a small pilot study to assess the feasibility of translating an ECoG-based interface to control complex neuroprosthetic devices in a severely disabled patient population. **Our underlying hypothesis is that ECoG recordings will allow disabled individuals to skillfully exert motor control of a wearable hand exoskeleton and generate verbal output using a computer communication interface.**

In a cohort of 8 subjects, we propose to record and monitor neural signals using subdural electrodes and a mounting and processing system which are already FDA cleared for the general indications of recording and monitoring of brain electrical activity. Specifically, the 510(k) documentation for the Blackrock NeuroPort system and Neuroport Array states that it "is for temporary (< 30 days) recording and monitoring of brain electrical activity" (Please see attached 510K approval K060523 and K090957). The 510K documentation for the PMT Subdural Cortical Electrodes states that it is "cleared for temporary (<30 day) use with recording, monitoring, and stimulation equipment for the recording, monitoring and stimulation of electrical signals on the surface of the brain" (Please see attached 510K approval K082474). The Blackrock NeuroPort Biopotential Signal Processing System and NeuroPort Electrode Array have been used in human translational clinical studies to record neural activity over a number of months and years and enabled tetraplegic patients to volitionally control computer cursor position and an anthropomorphic prosthetic limb with 7 degrees-of-freedom (Ajiboye et al. 2012, Bouton et al. 2016, Collinger et al. 2013, Hochberg et al. 2012b, Hochberg et al. 2006, Kim et al. 2008b, Simeral et al. 2011). The PMT Subdural Cortical Electrodes have been used extensively in patients for epilepsy monitoring. The combination device of Blackrock Microsystems NeuroPort system, Neuroport Array pedestal, and PMT Subdural Cortical Electrodes has been implanted in a non-human primate to successfully monitor overt reaching movements for 666 days without signal degradation or without significant damage to the brain cortex. We propose to perform long-term monitoring and testing with the Blackrock/PMT combined system over similar time scales as the aforementioned studies, with the aim of motor and communication control.

We further propose that our investigational device will bear the statement, "CAUTION – Investigational device. Limited by United States law to investigational use."; all devices that will be used in this study will be labeled for investigational use and all labels and labeling will contain the investigational device statement .

Upon UCSF Institutional Review Board (IRB) approval of our investigational plan and protocol, we propose to conduct testing in two phases for each subject. We anticipate that study visits will be conducted in the outpatient office and/or home setting based on participant preference and other practicalities.

This is a single-center study of the use of a ECoG-based neural interface for testing the feasibility of using ECoG signals to control complex devices for motor and speech control in adults affected by neurological disorders with severely impaired motor and communication.

PMT Subdural Cortical Electrodes will be surgically placed directly on the brain surface over the motor and language cortices of subjects with severe disorders of motor control and communication. After implantation of the electrode, study patients will undergo training and assessment of their ability to control a wearable hand robotic exoskeleton and determine if

ECoG brain signals can be decoded for language communication. This will be performed in two phases.

Phase 1: Optimize BCI system

In Phase 1, we will optimize the entire system to reliably detect neural activity to ensure that the recorded signals are stable and free of artifacts. Moreover, we will ensure that the neural signals can be converted in real-time into cursor or external object movements. This phase will be conducted in the outpatient office setting and/or the patient's home environment, based on patient preference and needs. We anticipate that this phase will take ~1 month; we will, however, specifically tailor the period for each subject.

Phase 2: Testing of BCI Control

In Phase 2, we will test feasibility for both neuroprosthetic control and for decoding speech from neural activity. We will begin experimental testing with the system for control of a custom wearable hand exoskeleton robot that is classified as a non-significant risk device. As outlined in the sections below, we have multiple safety features to ensure that there is minimal risk for injury during the embodiment phase (i.e. the subjects are interacting with the exoskeleton). Throughout this period, neural signals will be recorded and analyzed, and tasks will be performed toward the development, assessment, and improvement of the neural interface system. We anticipate that testing will be conducted in the outpatient office or home setting based on the patient's preference and needs. In this phase, we will also continue to perform experimental testing with the communication interface; we will assess the ability to control a communication interface. We anticipate that this phase will take a minimum of 10-11 months.

2.0 Patient Eligibility

Study subjects will be adults with severe motor impairment secondary to a neurological disorder.

Inclusion Criteria

- Age > 21
- Limited ability to use upper limbs, based on neurological examination, due to stroke, amyotrophic lateral sclerosis (ALS), multiple sclerosis, cervical spinal cord injury, brainstem stroke, muscular dystrophy, myopathy or severe neuropathy
- Disability, defined by a 4 or greater score on the Modified Rankin Scale, must be severe enough to cause loss of independence and inability to perform activities of daily living.
- If stroke or spinal cord injury, at least 1 year has passed since onset of symptoms
- Must live within a two-hour drive of UCSF

Exclusion Criteria

- Pregnancy or breastfeeding
- Inability to understand and/or read English
- Inability to give consent
- Dementia, based on history, physical exam, and MMSE
- Active depression (BDI > 20) or other psychiatric illness (active general anxiety disorder, schizophrenia, bipolar disorder, obsessive-compulsive disorder (OCD), or personality disorders (e.g. multiple personality disorder, borderline personality disorder, etc.)
- History of suicide attempt or suicidal ideation
- History of substance abuse
- Co-morbidities including uncontrolled hypertension, cancer, or major organ system failure.
- Ongoing anticoagulation which cannot be stopped in the peri-procedural period.
- Inability to comply with study follow-up visits
- Any prior intracranial surgery
- History of seizures
- Immunocompromised
- Has an active infection
- Has a CSF drainage system or an active CSF leak
- Requires diathermy, electroconvulsive therapy (ECT), or transcranial magnetic stimulation (TMS) to treat a chronic condition
- Has an implanted electronic device such as a neurostimulator, cardiac pacemaker/defibrillator or medication pump
- Allergies or known hypersensitivity to materials in the Blackrock NeuroPort Array pedestal (i.e. silicone, titanium) or the PMT Subdural Cortical Electrode (silicone, platinum iridium, nichrome)

3.0 Study Device(s)

Cleared for same indication with off-label use of device for at least 1 year:

- Blackrock NeuroPort Array, PN 6248
- Blackrock NeuroPort system, PN 5416
- PMT Subdural Cortical Electrodes, Model #2110TX-128-005

Modifications to Blackrock NeuroPort System with same indication with off-label use of device for at least 1 year:

- Digital Hub, 10480
- NeuroPlex E, 10908

4.0 Study Procedure

Study Duration: The duration of this pilot study will be 6 years. We expect that all 8 patients will be recruited in the first 4-5 years, and data collection, device development and analysis will be completed in 6 years.

Patient recruitment and clinical characterization: Patients with motor impairments secondary to neurological disorders will be recruited from clinics specializing in the treatment of stroke, ALS, and general neurological disorders, at UCSF and the San Francisco VA Medical Center.

Enrollment procedures: Prior to enrollment into the study, an informal phone interview to schedule an office-based evaluation will take place, followed by three outpatient screening visits. During the first outpatient visit we will describe the trial in detail and answer all questions. Should the participant choose to continue, we will schedule another visit to conduct a physical exam and to perform screening labs to determine eligibility. During this visit we will screen for eligibility by 1) acquiring patient demographics, 2) reviewing medical history and measuring vital signs, 3) ensuring patients are not currently pregnant or plan to become pregnant, 4) obtaining a list of current medications being taken, 5) ensuring MMSE scores are within a reasonable range (≥18, also accounting for motor difficulties with taking the test), 6) obtain baseline patient-rated and investigator rated clinical global impression scales, 7) assess baseline health status with SF-36 Health Survey, 8) assess current depression and anxiety states with Beck Depression and Anxiety inventories (BDI and BAI, respectively), 9) determine suicidal ideation risk is minimal with Columbia Suicide Severity Rating Scale (C-SSRS) 10) perform a baseline neurological physical exam and 11) determine the disability rating using the modified Rankin Scale. An MRI and CT of the brain will also be obtained for future surgical planning and to further determine eligibility. Moreover, a ECG and chest x-ray will also be obtained. We will then schedule a third follow-up visit to review this data and to answer remaining questions.

Pre-operative care: Subjects will be administered Kefzol 2g IV within 60 minutes prior to surgical incision, and continued for 24 hours post-operatively. In case of allergy to Kefzol, Vancomycin 1gm IV will be used.

Surgery: After obtaining informed consent, subjects will undergo brief general anesthesia (typically ~ 3-4 hours) for the surgical procedure. This may be either IV or inhaled anesthetics. Patient will be given IV antibiotics prior to incision, and re-dosed if required. Induction and wake-up will take place in the operating room, and patients will be monitored in the post-anesthesia care unit (PACU) after surgery for 2-3 hours during the peri-operative period. Subject will then continue to recover on the surgical ward for 2 days.

Subjects will undergo surgical placement of an ECoG array over the nondominant (usually right-sided) sensorimotor and speech cortices. The basic operative procedure is similar to what is commonly performed for non-penetrating subdural grid placement in patients with intractable epilepsy, which is well tolerated and has few complications [35]. This surgery will be smaller in exposure given that coverage will be limited to the regions of interest, and not broadly applied as used for epilepsy localization. Standard procedures for craniotomy at the surgical site will be followed. Briefly, a 5-6 cm curvilinear incision will be made over the anatomic hand representation of the cortex (i.e."hand knob") which is located 3 cm lateral to the midline. Localization will be confirmed using intraoperative Brainlab stereotactic

neuronavigation. A craniotomy will then be performed, exposing the dural surface. A wide slit in the dura will be opened.

After identifying the location for the electrode grid implant, the position for the pedestal connector is determined on the adjacent or contra-lateral skull surface and marked. A separate 2 cm scalp incision will be made for the pedestal connector. Holes will be drilled for connector placement and the connector will be secured to the skull with 8 small titanium screws. Once the connector is placed, the electrodes and wire bundle are gently manipulated to position the electrodes so that they are resting on the cortical surface over the area of interest. The nonpenetrating ECoG microarray will be sutured to the dura to secure its position. After successful placement of the electrode grid, the dura will be sutured closed in a watertight fashion and the bone flap will be replaced and secured in place with a standard titanium cranial fixation plates and screws.

The surgical site will be irrigated with antibiotic lactated ringers solution. The fascia and skin will be closed with absorbable sutures over the covered craniotomy with a slit accommodating the passage of the connector. The wound will be dressed with a sealed surgical bandage. The expected blood loss for this procedure is 50 cc. The expected operative time is 3-6 hours.

The ECoG electrode array is manufactured by PMT Corporation, and is already FDA-cleared for temporary (<30 days) clinical monitoring of neural signals (K082474). The PMT Subdural Cortical Electrodes are a chronically implantable array containing 128 electrodes, with an electrode spacing of 4 mm, capable of recording from areas of the central nervous system for extended periods of time. The electrode contacts are composed of medical grade platinum iridium, and embedded in a thin sheet of medical grade silastic. The model number 2110 indicates a platinum iridium wire and platinum iridium contacts. Platinum iridium is a mechanically robust alloy electrode material used in commercial DBS leads (Petrossians, Whalen and Weiland 2016). The electrodes' contacts are molded into a silicone rubber matrix in a fixed pattern. This identical electrode is used routinely at our institution and others for seizure localization. In the past ten years, we have not encountered any adverse inflammatory reactions or bleeding specific to the electrode arrays itself (over 100 cases). Insulated wires extend from each electrode through a flexible silicone tube to the connector on the pedestal.

Post-Operative Care: Subjects will be recovered in the intensive care unit and observed for 24 hours before transferring to the ward. A postoperative head CT will be obtained to evaluate for any hemorrhage and to confirm the position of the array. IV antibiotics (Kefzol will used if there are no allergies) will be administered for 24 hours after surgery, followed by prophylactic antibiotics given up to suture removal plus 2 days. Patients will be switched to oral antibiotics as soon as possible. Subjects will undergo physical exam every day with monitoring of vital signs, neurological exam, and basic respiratory, cardiac, and gastrointestinal exams. In addition, the wound site will be inspected at all study visits.

Routine hygiene will consist of handwashing with soap/water and donning gloves using sterile technique when coming in contact with the percutaneous connector. The caregiver and patient will be trained to clean the pedestal site according to specific instructions that will be given to them, to be performed at least once every seven days or as required.

An infection control protocol will be strictly followed. At early signs of infection or irritation the patient/caregiver should contact the study physician immediately. The surgical site will need to be cleaned twice or more during the day, while using extra meticulous hand hygiene. If skin erythema, edema, pain and/or warmth are present, the available drainage will be cultured and oral antibiotics will be started. Plain radiographs of the involved area, ESR, CRP,

WBC, and blood cultures will be obtained. If there is no improvement within 72 hours, rapid progression of erythema, symptoms worsen, or if there are signs of systemic toxicity, parenteral antibiotics is warranted. If high fever or severe pain is present, nuchal rigidity, or progressive deterioration in level of consciousness, the participant should go to the hospital emergency room. All implanted hardware will be surgically removed if there is evidence of hardware infection.

Pain related to small craniotomies is usually self-limited. Pain scale ratings will be assessed every 4 hours and routine postoperative pain management will be used. This includes the following medications as needed: acetaminophen and/or Percocet (acetaminophen/hydrocodone). IV pain meds (morphine sulfate or dilaudid) will also be administered if needed. In our experience, most patients do not require IV analgesics beyond the first operative day for smaller craniotomies.

Study visits: As outlined above, system testing will occur through two phases. In the first phase, we will simply optimize the system; in the second phase, we will commence testing of the motor and communication neural interface systems.

In **Phase 1**, the main goal is to ensure reliable neural signal monitoring and optimization of the real-time systems. Initial study visits will occur at defined time points, similar to those normally used in clinical care. For example, we anticipate a visit at post-op day (POD) 10 and 14. During this time, we will simply monitor the neural signals and briefly test the real-time communication with the computer interface. We anticipate additional visits 1-3x/week, based on the patient's availability and preference, to continue to monitor signal stability and check for wound healing. During this phase, we will also offer in home testing of the system to minimize the burden. While the equipment may be kept at the subject's home, testing will only occur when study personnel are present. The total time period of Phase 1 will be approximately 1 months, the exact time will be customized for each patient.

In **Phase 2**, we will test feasibility for both neuroprosthetic control and for decoding speech from neural activity. We will begin to perform experimental testing with the system for control of a custom wearable hand exoskeleton robot that can be classified as a non-significant risk device. As shown in panel A, the exoskeleton system consists of a table top frame that allows x,y,z movement of the arm/hand (i.e. supported by a brace attached to the platform mount). Movements are limited to the natural workspace of each subject. The hand exoskeleton (panel B) will be mounted using a readily releasable magnetic mount. The hand system aims to allow control of the fingers and thumb using a motorized cable system attached to motors. This system will allow us to test restoration of reach to grasp functions in our subjects. As outlined in the Risk Analysis (Appendix D), we have multiple safety features to ensure that there is minimal risk for injury during the embodiment phase (i.e. the subjects interact with the exoskeleton).

Throughout this period, neural signals will be recorded and analyzed, and tasks will be performed toward the development, assessment, and improvement of the neural interface system. We will assess quality of performance using kinematic parameters while performing required tasks. We will analyze stability of neural recordings and performance. To measure stability of the neural representation we will analyze the neural correlates of imagined movements. We will also examine the stability of neural correlates on neuroprosthetic exoskeleton movements (e.g. spectral content, timing, spatial recruitment). We will assess changes in the spatial correlation scales and other redundancy measures during learning and stable performance. We anticipate that testing will be conducted in the outpatient office or home setting based on the patient's preference and needs.

In this phase, we will also continue to perform experimental testing with the system for control of a virtual communicating interface. Throughout this period, neural signals will be recorded and analyzed, and tasks will be performed toward the development, assessment, and improvement of the neural interface system. We will assess the ability to control a computer communication interface. To measure stability of the neural representation we will analyze the neural correlates of imagined speech. We will also examine the stability of neural correlates on a neuroprosthetic communication device (e.g. spectral content, timing, spatial recruitment).

Neural activity monitoring: Continuous neural signal data will be acquired from the 128-channel implanted PMT Subdural Cortical Electrodes and processed with the Digital NeuroPort system hardware by Blackrock Microsystems. Broadly, the neural data will consist of ECoG neural activity from neurons in the vicinity of each recording electrode. The neural data will be actively monitored and recorded via the graphical user interface associated with the NeuroPort hardware commercially available via Blackrock. Data read and download are non-invasive and will be performed with the patient comfortably rested.

The NeuroPort system has been successfully deployed in monitoring neural activity in patients with motor control disorders (e.g. (Hochberg et al. 2012a, Pandarinath et al. 2015)). Blackrock Microsystems provides commercial software that allows real-time filtering, recording, and visualization of acquired neural data and also allows interfacing with other programming languages such as MATLAB (Mathworks, MA). Together, this allows the ability to create custom software such as communication device based on neural spiking activity, detailed further in the following section. Overall, the mix of commercial and custom software will allow for real-time signal processing, synchronization and control of the peripheral communication device, with parallel data streams to store data for offline analyses.

Online BMI control of a Wearable Hand Robotic Exoskeleton Device:
ECoG signals will be filtered and processed in real-time using a customized portable multi-channel neurophysiology workstation NeuroPort Biopotential Signal Processing System. We will bandpass each channel into multiple bands. Past experiments, including our own, suggest that movement related information is encoded in these bands.

Initialization Phase. We will use an adaptive filter to create a 'decoder' that maps neural activity to movement of the wearable exoskeleton device. Recent experiments suggest that such a filter can rapidly allow control of neuroprosthetic devices. We will train the filter using 'imagined movements'. As paretic/paralyzed patients will not have access to normal overt movement related neural signals, we will use the neural basis of imagined movements for training. We anticipate that during the initial training phase, the filter will establish a set of weights between the object and neural signals. During this training phase, patients will observe a computer cursor on a screen. In order to compare visual versus visual + proprioceptive/tactile feedback signals during decoder conditioning, we will use the exoskeleton setup for decoder conditioning. The arm and hand will move in a stereotyped fashion while the subjects are instructed to 'imagine' actively tracking its path. Of note, we have experience with similar control of an exoskeleton system ((Ganguly et al. 2011, Ganguly et al. 2009)). As prior and as outlined below, the current system is developed by UC Berkeley. They have long-standing experience with kinematic monitoring, limb dynamics and exoskeleton development (Matthew et al. 2015, Matthew et al. 2016, Oskarsson et al. 2016).

Training Phase. Subjects will be allowed to practice tasks associated with arms/hand and object manipulation. The position of the exoskeleton end effector coordinates (x, y, z) will

be under direct neural control. Preliminary experiments with an exoskeleton system showed that end effector control (position and orientation of the wrist) was more intuitive and efficient then position control of individual joints. For this initial phase, the motion will be restricted to a 2-dimensional plane for reaching and grasping objects. There are currently 9 tasks which involve interactions with both static and dynamic environments with various fixtures. We will first limit movements to a 2D environment. The additional degree of freedom involving grasp will be included based on proficiency. In addition, given that 'motivation' and reward are known to influence the overall learning process, a gamed-based training environment with specific goals and scoring systems were developed to engage the subject intellectually and to provide additional enrichment during the training phase.

Testing Phase. To assess robustness and stability of control over days we will assess performance characteristics in three tasks. A) Standard center-out task where subjects have to move to the center, engage a grasp, then move to a target and disengage the grasp to release of an object such as a ball. The workspace will be at 95% of the patient's natural reach. Target size will be kept at 5 cm. B) Reaching from a randomized starting and end position in the workspace of Task A. C) Task B except with the need to plan around obstacles that are placed in the direct path.

Online BMI control: Also during phase 2, neural activity will be used to control a real-time communication device using state of the art closed-loop decoders based on rapid changes in neural activity (Shanechi, Orsborn and Carmena 2016). The main advantage of such a decoder lies in its enhanced ability in discriminating user intent and its speed of operating at every event. Such decoders operate at much higher speeds (typically at 200Hz) over previously developed decoders that rely on averaging neural activity (typically at 10Hz). In addition, we will compare this decoder to more standard decoders (e.g. the Weiner Filter, the LMS filter, the Kalman filter and variants). As documented below, the main outcome measure will be the rate of communication using these approaches.

A virtual communication effector will be presented on a computer screen, custom written in the MATLAB programming environment. The interface between MATLAB and the Digital NeuroPort system will be via commercial software provided by Blackrock Microsystems. The novel decoder will map neural activity to the communication interface. A language processing engine will be concurrently running in the background to model and predict the words and sentences. The following metrics will be utilized to measure performance of the decoder and communication device: selections per minute, accuracy, correct characters per minute (Bacher et al. 2015b) and the bitrate, an information theoretic approach to relate accuracy, time of task completion and complexity (Nuyujukian et al. 2014b, Thompson et al. 2014a). The performance of the novel decoder based communication device will be compared to traditional state space filtering decoders such as the Kalman Filter that has been previously successfully deployed in similar BMI paradigms (Gilja et al. 2012, Bacher et al. 2015b).

Development life cycle of the BMI motor control and communication software:
a) Scope: The intended use of the decoder is to allow the patient to achieve control of external devices and thereby select characters and letters to form sentences, as well as control a wearable hand exoskeleton. As such the operation of the BCI is therefore dependent on the functionality of the software.
b) Platform: The software will be developed on MATLAB (The Mathworks, MA) and MATLAB supported C/C++ complied programs (MEX files) and will be running on the data acquisition PC that interfaces with the Blackrock NeuroPort Array pedestal connector. We will use the

software libraries that are part of the Blackrock Digital NeuroPort system to stream neural data into MATLAB in real-time.

c) Inputs and outputs: The inputs to the software will be the neural signals from the PMT Subdural Cortical Electrodes grid and the output of the software will consist of user controlled (via the user's neural signals) effector position, selections of characters, letters and numbers for communication purposes, in addition to control of a movements of a wearable hand exoskeleton.

d) Components: There are four distinct aspects of the software, three that operate 'behind the scenes' and one that serves as a Graphical User Interface for display. First, is the decoder itself that translates neural signals into user intentions. Second, is another parallel decoder that serves to discriminate when the patient has made a particular selection (or e.g. grasping actions). Third, is the software engine, that keeps track of the current selections made and generates a list of probable options using a statistical model of movement direction and language (Nadkarni, Ohno-Machado and Chapman 2011b). The fourth and final aspect of the decoder is the Graphical User Interface (GUI) that displays and controls the real-time position of end effector.

e) Safety: The software provides only visual feedback to the user and does not directly interface with the neural signal data acquisition process. The software only serves to allow the use to control the communication interface and the exoskeleton position.

f) Planning phase: In the planning phase, we will identify off-the-shelf components (such as language processing engines) and will aim to further refine and customize it in-house concurrently with the decoders and GUI.

g) Development phase: In the development phase, all components will be developed in parallel as discrete subunits of the overall functional system. A code repository will be maintained to keep track of the life cycle versions and code will be commented wherever appropriate. During the development phase, debugging will be performed at every iteration and documented. The documentation and code will be maintained on secure hard drives.

h) Testing phase: There are two aspects of this phase. One is the performance testing of each of the four individual components and the other is the testing of the entire software. In lieu of actual neural signals, simulated neural signals will be delivered as input, with a known mapping between the input and output state as the ground truth is known a priori. This will allow testing the performance of the decoders (accuracy in estimated positions). The testing of the GUI and the software engine will be performed independently of the decoders by manually controlling the position of the effector. The testing of the GUI and the software engine will be assessed by the stability and reliability in updating effector position and exoskeleton movements, in the turnaround time of displaying the list of predictive words and actions based on current selections. The overall system testing will employ a combination of simulated neural signals and manual position control to assess the ability of the software in allowing a user to communicate and control a hand neuroprosthetic.

i) Error handling: Code will be written to specifically monitor potential sources of errors in real-time decoding due to either noise in neural signals or decoder weight drift that would necessitate recalibration and resetting of the GUI and software engine.

j) Software validation: The validation and formal design review for the overall software will be performed by members of the PI's laboratory not involved with the development and testing of the software prior to software deployment.

k) Resolution and maintenance: Active documentation and daily logs will be noted to keep track of the performance of the software and address issues such as version control, robustness and immediate resolution of unforeseen errors in the software.

Progression of phases: We anticipate phase 1 will last approximately 1month, but will vary based on each subject. Phase 2 will last at a minimum 10-11 months, a total amount of time of at least 1 year after PMT Subdural Cortical Electrodes implantation and neural interface monitoring and testing, a timeframe which has been performed or exceeded without adverse

effects by previous studies using the PMT Subdural Cortical Electrodes and Blackrock Microsystems NeuroPort Array pedestal and NeuroPort system. In this study, recordings were made over 666 days in a non-human primate with no adverse events related to the implanted devices (Degenhart et al. 2016). The ECoG array is identical to that used for subdural grid placement in patients with intractable epilepsy, which is well tolerated and has few complications (Chang et al. 2010).

Activation of the brain recording function: All data collection in the study visits will be initiated by the study staff.

Conclusion of study: For each enrolled subject, if there have been no serious adverse events, we will present the option to continue with the study at the end of a 1-year period. If the subject chooses, we will continue with testing for another year. We will formally present this option every year for a period of 5 years. Notably, the subject will be reminded that he/she will have the option for surgical removal of the device at any point.

Removal of the ECoG grid and Connector pedestal: At the conclusion of the study, or earlier if medically indicated, the subdural cortical electrodes and connector pedestal will be surgically removed. The skin incision and bone flap will be reopened and the electrode will be removed and discarded. The dura will be sutured tightly. The galea and scalp will be sutured closed. The expected blood loss is minimal (less than 10 cc), and the expected operative time is 30 minutes.

5.0 Clinical Measurements and Procedures

Primary

This is a pilot study to test feasibility in eight subjects.

For BCI motor control, we will use outcome measures that are frequently used in preclinical studies of neuroprostheses (e.g. accuracy and reliability of cursor and limb control). As outlined below, the primary goal will be to gather statistics regarding the best achievable control using ECoG signals and state-of-the-art methods to allow motor neuroprosthetic control. For each of the parameters below we aim to describe the statistics as the mean and the variance (Bacher et al. 2015a, Nadkarni et al. 2011a, Nuyujukian et al. 2014a, Shanechi et al. 2016, Thompson et al. 2014b). Ultimately, we will compare these values to a wealth of published data regarding movements in able-bodied subjects, e.g.(Bacher et al. 2015a).

1. *Quality of performance* will be assessed using kinematic parameters while performing the required tasks. We will assess stability of the trajectories in the tasks. We will then assess ability for generalization from any region in the workspace to another random point. Position errors from the selected trajectory of the task as well as velocity and acceleration will be studied in both joint space and the end effector space.

2. *Recording and Performance Stability.* We will use previously established metrics to analyze stability of neural recordings (Shanechi et al. 2016, Thompson et al. 2014b) and performance. To measure stability of the neural representation we will analyze the neural correlates of daily imagined movements. We will also examine the stability of the neural correlates of neuroprosthetic movements (e.g. spectral content, timing, spatial recruitment).

3. *Spatial Scale.* An important question for ECoG recordings is the optimal spatial scale for the electrode grid. This has implications for maximizing the amount of information that can be obtained from the recording setup but also for defining the design specifications of implantable electronics (e.g. power requirements could vary greatly depending on the spatial and temporal resolution of the neural data required). We will look at changes in the spatial correlation scales and other redundancy measures during learning and stable performance.

The following metrics will further be utilized to measure performance of the decoder and communication device: selections per minute, accuracy, correct characters per minute (Bacher et al. 2015b) and the bitrate, an information theoretic approach to relate accuracy, time of task completion and complexity (Nuyujukian et al. 2014b, Thompson et al. 2014a). Physiological measurements related to neural activity will include statistical assessments of z-scored activity from single electrodes as well as population dynamics. Physiological measurements related to oscillatory activity will include: wide spectrum power-spectral analysis as well as using specific frequency domain for mean log power (i.e. in the delta, theta, alpha, beta, gamma bands), coupling between the phase of low frequency rhythms and broadband gamma amplitude (phase-amplitude coupling, abbreviated PAC) (Canolty et al. 2006, Miller et al. 2010, Tort et al.).

Clinical measures and physiological measurements will be collected and recorded by members of the research and clinical team.

6.0 Data Management

All clinical and physiological data will be stored in encrypted and password-protected computers in the PI's laboratory that is always locked. If the Digital NeuroPort system is stored in the participant's residence, all research data will be maintained in accordance with UCSF standard encryption policy. In publications or presentations of the data, data will be grouped by case number in chronological order with no name identification. All patients will be asked to sign a separate consent for audio-video recording. When presenting videotape data at scientific conferences, we will utilize only videos from patients who have consented to have their videos shown. De-identified electrophysiological data may be shared with other researchers at other institutions.

7.0 Statistical Methods and Data Analysis

To assess the performance of the decoder, analyses will be performed on the kinematics associated with control, such as time to reach a target, trajectory curvature etc., in conjunction with measures associated with the communication device such as the bit rate, accuracy, characters per minute. The statistical reliability of the decoder will be assessed by non-parametric data permutation wherein the learned mapping between neural activity and the effector position will be artificially broken down and shuffled. Field potential data will be analyzed using wide spectrum power-spectral analysis as well as using specific frequency domain for mean log power (i.e. in the delta, theta, alpha, beta, gamma bands). We will also examine coherence and cross-frequency coupling between the channels (Canolty et al. 2006, Miller et al. 2010, Tort et al.). Using a repeated measures ANOVA statistical analysis, summary statistics for power in relevant frequency bands, control related power changes, and indices of phase-amplitude coupling will be compared at different time-points of control. Additionally, bootstrap statistical tests and general linear mixed models can be utilized to investigate potential statistical effects, given the small sample size. Mean, median, variance and median absolute deviation describing the statistics of each of the measured outcome (such as accuracy, effector position control) will be recorded for each subject.

Sample size calculation: This is a pilot study to assess the feasibility of an ECoG based implantable BCI device in patients with motor control disorders using intracranial recordings, a communication interface and a wearable hand exoskeleton. The collected pilot data will aid in determining the feasibility, reliability and future directions of the brain machine interface for communication and motor control. In addition, the pilot data will be used to formulate more detailed hypothesis on neural plasticity and BMI control in humans. As such, there is no formal sample size requirement for this pilot study.

Criteria for study success that would justify a larger subsequent trial:

1) Ability to use ECoG-based neural activity to control a neuroprosthetic device and communication interface.

2) No permanent serious adverse events occur (such as trauma with long-term motor deficit).

3) Benefits to the patient in regaining a sense of control over the ability to exert motor control and communicate in an efficient manner.

8.0 Regulatory Requirements

Prior to the start of the study, the following documents will be collected and filed:
- Signed protocol signature page
- Curriculum vitae of the PIs and Sub-investigators, updated within 2 years
- Current medical licenses for the PIs and all Sub-investigators
- Financial disclosure form signed by the PIs and all Sub-investigators
- Copy of the IRB approval letter for the study and the IRB Membership List
- Investigator Agreement

Investigator Obligations

Redacted will be responsible for ensuring that all study site personnel, adhere to all FDA regulations and guidelines regarding clinical trials, including guidelines for GCP (including the archiving of essential documents), both during and after study completion. Additionally, they are responsible for the subject's compliance to the study protocol.

All information obtained during the conduct of the study with respect to the patients' state of health will be regarded as confidential. This is detailed in the written information provided to the patient. An agreement for disclosure of any such information will be obtained in writing and will be signed by the patient.

Informed Consent

The investigators will obtain and document informed consent for each patient screened for this study. All patients will be informed in writing of the nature of the protocol and investigational therapy, its possible hazards, and their right to withdraw at any time, and will sign a form indicating their consent to participate prior to the initiation of study procedures.

Institutional Review Board

This protocol and relevant supporting data are to be submitted to the appropriate IRB for review and approval before the study can be initiated (UCSF, Human Research Protection Program, 3333 California Street, Suite 315, San Francisco, CA, 94118, FWA#00000068; IRB Registration 00000229, Lisa Denney, HRRP Director). Amendments to the protocol will also be submitted to the IRB prior to implementation of the change. The PIs are responsible for informing the IRB of the progress of the study and for obtaining annual IRB renewal. The IRB must be informed at the time of completion of the study and should be provided with a summary of the results of the study by the PIs. The PIs must notify the IRB in writing of any SAE or any unexpected AE according to ICH guidelines.

Data safety monitory board (DSMB) and safety monitoring plan

Treatment emergent adverse events that are assessed by the principal investigators as possibly, probably, or definitely related to surgical implantation or chronic cortical recording AND are unexpected or meet seriousness criteria (death, immediately life threatening, hospitalization >24 hours, persistent or significant disability, or significant intervention required to prevent one of the previously-stated outcomes) will be recorded and reported to the IRB, device manufacturer and the FDA via the MedWatch online voluntary reporting form within 10 working days of the study team's knowledge of the event.

All such events will also be reported to the data safety monitor board (DSMB), led by a neurosurgeon at our home institution, who does not have direct involvement in this study but who has expertise in implantable devices, pain management and neurosurgery. The DSMB will meet regularly to review data related to the clinical trial, provide guidance and feedback, and review any adverse event reports. Treatment-related adverse events assessed as definitely, probably, or possibly related to study procedures and either serious or unexpected, noted by any study personnel will be reported within 10 working days of their knowledge of the event to the DSMB. The DSMB will then advise the PI on potential changes in procedures to improve safety. The safety endpoint will consist of all adverse events.

Throughout the clinical trial, should a serious adverse event occur that is assessed to be related to the presence or surgical implantation of the electrode system, such as infection, the device will be removed and the study halted for the patient. Removal will be accomplished by re-opening the original incisions, temporary removal of the bone, and removal of the PMT Subdural Cortical Electrodes from the brain and NeuroPort Array pedestal from the skull. The dura will be re-sewn together and the bone fixed again with titanium screws

Furthermore, if there is a serious surgical or non-surgical adverse event, or with the onset of suicidality, the study will be halted for the patient.

If two patients meet one or more of these criteria (i. serious surgical or nonsurgery-related adverse event, or ii. onset of suicidality), the study will be halted until information is reviewed by the DSMB and FDA.

9.0 References

Ajiboye, A. B., J. D. Simeral, J. P. Donoghue, L. R. Hochberg & R. F. Kirsch (2012) Prediction of imagined single-joint movements in a person with high-level tetraplegia. *IEEE Trans Biomed Eng*, 59, 2755-65.

Anderson, K. D. (2004) Targeting recovery: priorities of the spinal cord-injured population. *J Neurotrauma,* 21, 1371-83.

Bacher, D., B. Jarosiewicz, N. Y. Masse, S. D. Stavisky, J. D. Simeral, K. Newell, E. M. Oakley, S. S. Cash, G. Friehs & L. R. Hochberg (2015a) Neural Point-and-Click Communication by a Person With Incomplete Locked-In Syndrome. *Neurorehabil Neural Repair,* 29, 462-71.

Bacher, D., B. Jarosiewicz, N. Y. Masse, S. D. Stavisky, J. D. Simeral, K. Newell, E. M. Oakley, S. S. Cash, G. Friehs & L. R. Hochberg (2015b) Neural point-and-click communication by a person with incomplete locked-in syndrome. *Neurorehabilitation and neural repair,* 29, 462-471.

Bensmaia, S. J. & L. E. Miller (2014) Restoring sensorimotor function through intracortical interfaces: progress and looming challenges. *Nat Rev Neurosci,* 15, 313-25.

Birbaumer, N., N. Ghanayim, T. Hinterberger, I. Iversen, B. Kotchoubey, A. Kubler, J. Perelmouter, E. Taub & H. Flor (1999) A spelling device for the paralysed. *Nature,* 398, 297-8.

Bouchard, K. E., N. Mesgarani, K. Johnson & E. F. Chang (2013) Functional organization of human sensorimotor cortex for speech articulation. *Nature,* 495, 327-32.

Bouton, C. E., A. Shaikhouni, N. V. Annetta, M. A. Bockbrader, D. A. Friedenberg, D. M. Nielson, G. Sharma, P. B. Sederberg, B. C. Glenn, W. J. Mysiw, A. G. Morgan, M. Deogaonkar & A. R. Rezai (2016) Restoring cortical control of functional movement in a human with quadriplegia. *Nature,* 533, 247-50.

Canolty, R. T., E. Edwards, S. S. Dalal, M. Soltani, S. S. Nagarajan, H. E. Kirsch, M. S. Berger, N. M. Barbaro & R. T. Knight (2006) High gamma power is phase-locked to theta oscillations in human neocortex. *Science,* 313, 1626-8.

Carmena, J. M., M. A. Lebedev, R. E. Crist, J. E. O'Doherty, D. M. Santucci, D. F. Dimitrov, P. G. Patil, C. S. Henriquez & M. A. Nicolelis (2003) Learning to control a brain-machine interface for reaching and grasping by primates. *PLoS Biol,* 1, E42.

Chang, E. F., J. W. Rieger, K. Johnson, M. S. Berger, N. M. Barbaro & R. T. Knight (2010) Categorical speech representation in human superior temporal gyrus. *Nat Neurosci,* 13, 1428-32.

Chao, Z. C., Y. Nagasaka & N. Fujii (2010) Long-term asynchronous decoding of arm motion using electrocorticographic signals in monkeys. *Front Neuroengineering,* 3, 3.

Chestek, C. A., V. Gilja, P. Nuyujukian, R. J. Kier, F. Solzbacher, S. I. Ryu, R. R. Harrison & K. V. Shenoy (2009) HermesC: Low-Power Wireless Neural Recording System for Freely Moving Primates. *Ieee Transactions on Neural Systems and Rehabilitation Engineering,* 17, 330-338.

Churchland, M. M., J. P. Cunningham, M. T. Kaufman, J. D. Foster, P. Nuyujukian, S. I. Ryu & K. V. Shenoy (2012) Neural population dynamics during reaching. *Nature,* 487, 51-6.

Collinger, J. L., S. Foldes, T. M. Bruns, B. Wodlinger, R. Gaunt & D. J. Weber (2013) Neuroprosthetic technology for individuals with spinal cord injury. *J Spinal Cord Med,* 36, 258-72.

Degenhart, A. D., J. Eles, R. Dum, J. L. Mischel, I. Smalianchuk, B. Endler, R. C. Ashmore, E. C. Tyler-Kabara, N. G. Hatsopoulos, W. Wang, A. P. Batista & X. T. Cui (2016) Histological evaluation of a chronically-implanted electrocorticographic electrode grid in a non-human primate. *J Neural Eng,* 13, 046019.

Ganguly, K. & J. M. Carmena (2009) Emergence of a stable cortical map for neuroprosthetic control. *PLoS Biol,* 7, e1000153.
Ganguly, K., D. F. Dimitrov, J. D. Wallis & J. M. Carmena (2011) Reversible large-scale modification of cortical networks during neuroprosthetic control. *Nat Neurosci,* 14, 662-7.
Ganguly, K., L. Secundo, G. Ranade, A. Orsborn, E. F. Chang, D. F. Dimitrov, J. D. Wallis, N. M. Barbaro, R. T. Knight & J. M. Carmena (2009) Cortical representation of ipsilateral arm movements in monkey and man. *J Neurosci,* 29, 12948-56.
Gilja, V., C. A. Chestek, I. Diester, J. M. Henderson, K. Deisseroth & K. V. Shenoy (2011) Challenges and opportunities for next-generation intracortically based neural prostheses. *IEEE Trans Biomed Eng,* 58, 1891-9.
Gilja, V., P. Nuyujukian, C. A. Chestek, J. P. Cunningham, M. Y. Byron, J. M. Fan, M. M. Churchland, M. T. Kaufman, J. C. Kao & S. I. Ryu (2012) A high-performance neural prosthesis enabled by control algorithm design. *Nature neuroscience,* 15, 1752-1757.
Hochberg, L. R., D. Bacher, B. Jarosiewicz, N. Y. Masse, J. D. Simeral, J. Vogel, S. Haddadin, J. Liu, S. S. Cash & P. van der Smagt (2012a) Reach and grasp by people with tetraplegia using a neurally controlled robotic arm. *Nature,* 485, 372-375.
Hochberg, L. R., D. Bacher, B. Jarosiewicz, N. Y. Masse, J. D. Simeral, J. Vogel, S. Haddadin, J. Liu, S. S. Cash, P. van der Smagt & J. P. Donoghue (2012b) Reach and grasp by people with tetraplegia using a neurally controlled robotic arm. *Nature,* 485, 372-5.
Hochberg, L. R., M. D. Serruya, G. M. Friehs, J. A. Mukand, M. Saleh, A. H. Caplan, A. Branner, D. Chen, R. D. Penn & J. P. Donoghue (2006) Neuronal ensemble control of prosthetic devices by a human with tetraplegia. *Nature,* 442, 164-71.
Homer, M. L., A. V. Nurmikko, J. P. Donoghue & L. R. Hochberg (2013) Sensors and decoding for intracortical brain computer interfaces. *Annu Rev Biomed Eng,* 15, 383-405.
Huggins, J. E., P. A. Wren & K. L. Gruis (2011) What would brain-computer interface users want? Opinions and priorities of potential users with amyotrophic lateral sclerosis. *Amyotroph Lateral Scler,* 12, 318-24.
Kennedy, P. R. (1994) 'Locked-in' patients. *Neurology,* 44, 366-7.
Kennedy, P. R. & R. A. Bakay (1998) Restoration of neural output from a paralyzed patient by a direct brain connection. *Neuroreport,* 9, 1707-11.
Kim, S. P., J. D. Simeral, L. R. Hochberg, J. P. Donoghue & M. J. Black (2008a) Neural control of computer cursor velocity by decoding motor cortical spiking activity in humans with tetraplegia. *Journal of Neural Engineering,* 5, 455-76.
--- (2008b) Neural control of computer cursor velocity by decoding motor cortical spiking activity in humans with tetraplegia. *J Neural Eng,* 5, 455-76.
Kubler, A., B. Kotchoubey, J. Kaiser, J. R. Wolpaw & N. Birbaumer (2001) Brain-computer communication: unlocking the locked in. *Psychol Bull,* 127, 358-75.
Leuthardt, E. C., K. J. Miller, G. Schalk, R. P. Rao & J. G. Ojemann (2006) Electrocorticography-based brain computer interface--the Seattle experience. *IEEE Trans Neural Syst Rehabil Eng,* 14, 194-8.
Leuthardt, E. C., G. Schalk, J. Roland, A. Rouse & D. W. Moran (2009) Evolution of brain-computer interfaces: going beyond classic motor physiology. *Neurosurg Focus,* 27, E4.
Leuthardt, E. C., G. Schalk, J. R. Wolpaw, J. G. Ojemann & D. W. Moran (2004) A brain-computer interface using electrocorticographic signals in humans. *J Neural Eng,* 1, 63-71.
Matthew, R. P., E. J. Mica, W. Meinhold, J. A. Loeza, M. Tomizuka & R. Bajcsy (2015) Initial investigation into the effect of an Active/Passive exoskeleton on hammer curl performance in healthy subjects. *Conf Proc IEEE Eng Med Biol Soc,* 2015, 3607-10.

Matthew, R. P., V. Shia, G. Venture & R. Bajcsy (2016) Generating physically realistic kinematic and dynamic models from small data sets: An application for sit-to-stand actions. *Conf Proc IEEE Eng Med Biol Soc,* 2016, 2173-2178.

Miller, K. J., D. Hermes, C. J. Honey, M. Sharma, R. P. Rao, M. den Nijs, E. E. Fetz, T. J. Sejnowski, A. O. Hebb, J. G. Ojemann, S. Makeig & E. C. Leuthardt (2010) Dynamic modulation of local population activity by rhythm phase in human occipital cortex during a visual search task. *Front Hum Neurosci,* 4, 197.

Monti, M. M., A. Vanhaudenhuyse, M. R. Coleman, M. Boly, J. D. Pickard, L. Tshibanda, A. M. Owen & S. Laureys (2010) Willful modulation of brain activity in disorders of consciousness. *N Engl J Med,* 362, 579-89.

Morrell, M. J. & R. N. S. S. i. E. S. Group (2011) Responsive cortical stimulation for the treatment of medically intractable partial epilepsy. *Neurology,* 77, 1295-304.

Nadkarni, P. M., L. Ohno-Machado & W. W. Chapman (2011a) Natural language processing: an introduction. *J Am Med Inform Assoc,* 18, 544-51.

Nadkarni, P. M., L. Ohno-Machado & W. W. Chapman (2011b) Natural language processing: an introduction. *Journal of the American Medical Informatics Association,* 18, 544-551.

Nicolelis, M. A. & M. A. Lebedev (2009) Principles of neural ensemble physiology underlying the operation of brain-machine interfaces. *Nat Rev Neurosci,* 10, 530-40.

Nuyujukian, D. S., J. Voutsinas, L. Bernstein & S. S. Wang (2014a) Medication use and multiple myeloma risk in Los Angeles County. *Cancer Causes Control,* 25, 1233-7.

Nuyujukian, P., J. C. Kao, J. M. Fan, S. D. Stavisky, S. I. Ryu & K. V. Shenoy (2014b) Performance sustaining intracortical neural prostheses. *Journal of neural engineering,* 11, 066003.

Oskarsson, B., N. C. Joyce, E. De Bie, A. Nicorici, R. Bajcsy, G. Kurillo & J. J. Han (2016) Upper extremity 3-dimensional reachable workspace assessment in amyotrophic lateral sclerosis by Kinect sensor. *Muscle Nerve,* 53, 234-41.

Pandarinath, C., V. Gilja, C. H. Blabe, P. Nuyujukian, A. A. Sarma, B. L. Sorice, E. N. Eskandar, L. R. Hochberg, J. M. Henderson & K. V. Shenoy (2015) Neural population dynamics in human motor cortex during movements in people with ALS. *Elife,* 4, e07436.

Pasley, B. N., S. V. David, N. Mesgarani, A. Flinker, S. A. Shamma, N. E. Crone, R. T. Knight & E. F. Chang (2012) Reconstructing speech from human auditory cortex. *Plos Biology,* 10, e1001251.

Schalk, G., K. J. Miller, N. R. Anderson, J. A. Wilson, M. D. Smyth, J. G. Ojemann, D. W. Moran, J. R. Wolpaw & E. C. Leuthardt (2008) Two-dimensional movement control using electrocorticographic signals in humans. *J Neural Eng,* 5, 75-84.

Schwartz, A. B. (2004) Cortical neural prosthetics. *Annu Rev Neurosci,* 27, 487-507.

Schwartz, A. B., X. T. Cui, D. J. Weber & D. W. Moran (2006) Brain-controlled interfaces: movement restoration with neural prosthetics. *Neuron,* 52, 205-20.

Selzer, M. E., S. Clarke, L. G. Cohen, G. Kwakkel & R. H. Miller. 2014. *Textbook of neural repair and rehabilitation*. Cambridge: Cambridge University Press.

Shanechi, M. M., A. L. Orsborn & J. M. Carmena (2016) Robust Brain-Machine Interface Design Using Optimal Feedback Control Modeling and Adaptive Point Process Filtering. *PLoS Comput Biol,* 12, e1004730.

Shenoy, K. V. & J. M. Carmena (2014) Combining decoder design and neural adaptation in brain-machine interfaces. *Neuron,* 84, 665-80.

Simeral, J. D., S. P. Kim, M. J. Black, J. P. Donoghue & L. R. Hochberg (2011) Neural control of cursor trajectory and click by a human with tetraplegia 1000 days after implant of an intracortical microelectrode array. *J Neural Eng,* 8, 025027.

Slutzky, M. W., L. R. Jordan, E. W. Lindberg, K. E. Lindsay & L. E. Miller (2011) Decoding the rat forelimb movement direction from epidural and intracortical field potentials. *J Neural Eng,* 8, 036013.

Spataro, R., M. Ciriacono, C. Manno & V. La Bella (2014) The eye-tracking computer device for communication in amyotrophic lateral sclerosis. *Acta Neurol Scand,* 130, 40-5.

Taylor, D. M., S. I. Tillery & A. B. Schwartz (2002) Direct cortical control of 3D neuroprosthetic devices. *Science,* 296, 1829-32.

Thompson, D. E., L. R. Quitadamo, L. Mainardi, S. Gao, P.-J. Kindermans, J. D. Simeral, R. Fazel-Rezai, M. Matteucci, T. H. Falk & L. Bianchi (2014a) Performance measurement for brain–computer or brain–machine interfaces: a tutorial. *Journal of neural engineering,* 11, 035001.

Thompson, D. E., L. R. Quitadamo, L. Mainardi, K. U. Laghari, S. Gao, P. J. Kindermans, J. D. Simeral, R. Fazel-Rezai, M. Matteucci, T. H. Falk, L. Bianchi, C. A. Chestek & J. E. Huggins (2014b) Performance measurement for brain-computer or brain-machine interfaces: a tutorial. *J Neural Eng,* 11, 035001.

Tort, A. B., R. Komorowski, H. Eichenbaum & N. Kopell (2010) Measuring phase-amplitude coupling between neuronal oscillations of different frequencies. *J Neurophysiol,* 104, 1195-210.

Wolpaw, J. R., N. Birbaumer, D. J. McFarland, G. Pfurtscheller & T. M. Vaughan (2002) Brain-computer interfaces for communication and control. *Clin Neurophysiol,* 113, 767-91.

Summary of Changes

1. Correction to our Clinical Protocol (page 30) wording to make it more precise and reflect the intended and appropriate device explantation criteria.

   Before Correction:
   "Throughout the clinical trial, should a serious adverse event occur that is assessed to be surgery related or not, or related to the presence of the electrode system, such as infection, the device will be removed and the study halted for the patient."

   After Correction:
   "Throughout the clinical trial, should a serious adverse event occur that is assessed to be related to the presence or surgical implantation of the electrode system, such as infection, the device will be removed and the study halted for the patient."

2. Change from the Blackrock Microsystems NeuroPort Biopotential Signal Processing system, otherwise known as the NeuroPort system (K060523, K090957), to utilize the newest versions of the Front End Amplifier and its power supply, and the Patient Cable, which are the Digital Hub 128 and its power supply and the NeuroPlex E, respectively.

3. Minor correction to the wording of one of the exclusion criteria.

   Before correction:

   - Co-morbidities including ongoing anticoagulation, uncontrolled hypertension, cancer, or major organ system failure.

   After correction:

   - Co-morbidities including uncontrolled hypertension, cancer, or major organ system failure.
   - Ongoing anticoagulation which cannot be stopped in the peri-procedural period.

4. Specified Photosensitive Epilepsy as a type of seizure disorder that is an exclusion criterion

Note about the exploratory nature of the clinical trial

This clinical trial is a Phase I single-center early feasibility study to evaluate the potential of ECoG-based neural interfaces for controlling advanced neuroprostheses that restore motor and communicative functions. Due to the exploratory nature of the trial and the limited number of trial participants, we did not pre-define specific methods and algorithms to evaluate using specific metrics. This is reflected in the primary endpoints for the efficacy assessments of the clinical trial, which are stated in the protocol as "Feasibility of control of a wearable exoskeleton device and a communication interface."

As a result, a variety of analysis methods will be applied to the datasets collected with the trial participants throughout the trial. Additionally, we did not formalize a statistical analysis plan alongside the protocol. In any reports (publications, presentations, etc.) of analyses that involve data collected as part of this clinical trial, the selection, measurement, statistical testing, and interpretation of outcome metrics will be informed by the relevant literature and performed to the highest standard of analytic and statistical rigor. This includes the present work, which describes a proof-of-concept spelling system controlled by silent attempts to speak and an ECoG-based neural interface with a single participant.
