# Supplementary material for: Generalizable spelling using a speech neuroprosthesis in an individual with severe limb and vocal paralysis
Source: Nat Commun. 2022 Nov 8;13:6510. doi: 10.1038/s41467-022-33611-3 (PMC9643551; doi:10.1038/s41467-022-33611-3)
Supplement: Supplementary file 3 — Description of Additional Supplementary Files [file 41467_2022_33611_MOESM3_ESM.pdf]

Description of Additional Supplementary Files

Supplementary Movie 1: Real-time spelling in a copy-typing task.

The participant spells out the prompted sentences using the spelling system. After each sentence prompt appears on the screen, he volitionally activates the system, spells the sentence by silently attempting to say the NATO code words associated with each letter, then volitionally deactivates the system, thereby finalizing the sentence. Both activation and deactivation of the system are controlled via decoded predictions from neural activity.

Supplementary Movie 2: Real-time spelling in a conversational question-and-answer task.

The participant spells out freely chosen responses to questions using silent attempts to say NATO code words associated with each letter in the response. As in the copy-typing task, he volitionally engages and disengages the spelling system. He nods his head after a decoded response was finalized to indicate that his response was correctly decoded.

Supplementary Movie 3: Real-time spelling in a conversational free-form task.

The participant spells out freely and spontaneously chosen sentences using the spelling system.

Supplementary Movie 4: Real-time plug-and-play spelling in a copy-typing task.

A researcher helps to prepare the participant for using the spelling system by connecting a digital headstage link to an implanted pedestal. After a brief task-setup period, and without any day-of model recalibration, the participant spells out the prompted sentences using the spelling system.
