# Supplementary material for: Generalizable spelling using a speech neuroprosthesis in an individual with severe limb and vocal paralysis
Source: Nat Commun. 2022 Nov 8;13:6510. doi: 10.1038/s41467-022-33611-3 (PMC9643551; doi:10.1038/s41467-022-33611-3)
Supplement: Supplementary file 8 — Reporting Summary [file 41467_2022_33611_MOESM8_ESM.pdf]

nature portfolio

Corresponding author(s): Edward Chang

Last updated by author(s): Jul 18, 2022

Reporting Summary

Nature Portfolio wishes to improve the reproducibility of the work that we publish. This form provides structure for consistency and transparency in reporting. For further information on Nature Portfolio policies, see our Editorial Policies and the Editorial Policy Checklist.

Statistics

For all statistical analyses, confirm that the following items are present in the figure legend, table legend, main text, or Methods section.

n/a	Confirmed	
☐	☒	The exact sample size (*n*) for each experimental group/condition, given as a discrete number and unit of measurement
☐	☒	A statement on whether measurements were taken from distinct samples or whether the same sample was measured repeatedly
☐	☒	The statistical test(s) used AND whether they are one- or two-sided<br>*Only common tests should be described solely by name; describe more complex techniques in the Methods section.*
☒	☐	A description of all covariates tested
☐	☒	A description of any assumptions or corrections, such as tests of normality and adjustment for multiple comparisons
☐	☒	A full description of the statistical parameters including central tendency (e.g. means) or other basic estimates (e.g. regression coefficient) AND variation (e.g. standard deviation) or associated estimates of uncertainty (e.g. confidence intervals)
☐	☒	For null hypothesis testing, the test statistic (e.g. *F*, *t*, *r*) with confidence intervals, effect sizes, degrees of freedom and *P* value noted<br>*Give P values as exact values whenever suitable.*
☒	☐	For Bayesian analysis, information on the choice of priors and Markov chain Monte Carlo settings
☒	☐	For hierarchical and complex designs, identification of the appropriate level for tests and full reporting of outcomes
☐	☒	Estimates of effect sizes (e.g. Cohen's *d*, Pearson's *r*), indicating how they were calculated

Our web collection on statistics for biologists contains articles on many of the points above.

Software and code

Policy information about availability of computer code

Data collection

Open source: Python 3.6

Commercial software: We used hardware manufactured by Blackrock Microsystems (along with the associated firmware and the NeuroPort Central Suite software package (version 7.0.4), the proprietary software suite used to control their hardware) to acquire and pre-process data from the neural implant.

Custom code: We used a real-time software package (referred to as rtNSR; described in prior literature that is referenced in the current manuscript) to process neural data in real time

Data analysis

Open source:
- Python 3.6
- NumPy 1.19.1
- scikit-learn 0.24.2
- pandas 0.25.3
- SciPy 1.5.4
- PyTorch 1.6.0
- NLTK 3.6.2
- lm-scorer 0.4.2

Custom code: We used custom scripts and modules to analyze the neural data and implement real-time prediction and language models.

Relevant code for reproducing figures and the main results have been made available on GitHub.

For manuscripts utilizing custom algorithms or software that are central to the research but not yet described in published literature, software must be made available to editors and reviewers. We strongly encourage code deposition in a community repository (e.g. GitHub). See the Nature Portfolio guidelines for submitting code & software for further information.

Data

Policy information about availability of data

All manuscripts must include a data availability statement. This statement should provide the following information, where applicable:

- Accession codes, unique identifiers, or web links for publicly available datasets
- A description of any restrictions on data availability
- For clinical datasets or third party data, please ensure that the statement adheres to our policy

Relevant data will be made available upon reasonable request, per the guidelines from our clinical-trial protocol which allow us to share de-identified data with researchers at other institutions but precludes us from making all of the data publicly available. Requests for relevant data can be made to Dr. Edward Chang (edward.chang@ucsf.edu). Responses can be expected within 3 weeks. Any provided data should be kept confidential and should not be shared with others unless approval to do so is obtained from Dr. Chang.

Source data to re-create the manuscript figures (including accuracies, statistical values, and cross-validation accuracies) are publicly provided with this publication.

Human research participants

Policy information about studies involving human research participants and Sex and Gender in Research.

Reporting on sex and gender	The single participant in the study is a male. Sex and gender effects were not researched in this study.
Population characteristics	The single participant in the study is a male of age 36 at the start of the study with severe spastic quadriparesis and anarthria caused by brainstem stroke.
Recruitment	Participants with motor impairments secondary to neurological disorders were recruited from clinics specializing in the treatment of stroke, ALS, and general neurological disorders, at UCSF and the San Francisco VA Medical Center.<br><br>Prior to enrollment into the study, an informal phone interview to schedule an office-based evaluation takes place, followed by three outpatient screening visits. During the first outpatient visit, we describe the trial in detail and answer all questions. Should the participant choose to continue, we schedule another visit to conduct a physical exam and to perform screening to determine eligibility. An MRI and CT of the brain is obtained for future surgical planning and to determine further eligibility. Additionally, an ECG and chest X-ray are also obtained. We schedule a third follow-up visit to review this data and answer remaining questions prior to enrollment in the trial.<br><br>To meet the eligibility criteria for enrollment in our trial, participants must fit specific clinical characteristics (see attached clinical protocol for full eligibility criteria). Therefore, we do not expect any noteworthy self-selection bias in this study or other studies that are part of this clinical trial, as participants who volunteer to participate will not differ from non-volunteers in any relevant clinical characteristics.
Ethics oversight	The study protocol was approved by the FDA, UCSF IRB, and the NIH.

Note that full information on the approval of the study protocol must also be provided in the manuscript.

Field-specific reporting

Please select the one below that is the best fit for your research. If you are not sure, read the appropriate sections before making your selection.

☒ Life sciences ☐ Behavioural & social sciences ☐ Ecological, evolutionary & environmental sciences

For a reference copy of the document with all sections, see nature.com/documents/nr-reporting-summary-flat.pdf

Life sciences study design

All studies must disclose on these points even when the disclosure is negative.

Sample size	Sample sizes for tests were determined by the amount of available data with the participant. The amount of data collected with the participant was dependent on our estimation of how much data (e.g. how many trials of the tasks) would be required to reasonably estimate the measurements of interest and perform statistical comparisons.
Data exclusions	Some sentence-spelling trials were excluded from the primary performance analyses if the participant reported making a mistake or, in the conversational condition of the sentence-spelling task, an intended word that the participant was attempting to spell was outside of the 1,152-word vocabulary. These data exclusions are described in more detail in the Methods section. We established these exclusion criteria

	after collecting the first sentence-spelling trials but before collecting the final sentence-spelling trials and before analyzing any of the data. For completeness, we also included measurements of the key performance metrics when these trials were included in the Methods section, and the outcomes were very similar.
Replication	During real-time evaluation of the spelling system, the participant attempted to use the spelling system during many different experimental blocks and in a variety of task conditions. Performance across blocks were reasonably consistent, as described in the manuscript. During offline analyses, we trained new decoding models on subsets of the available data multiple times during cross-validation. This is also described in the manuscript. However, these do not provide definitive evidence of reproducibility. True replication of the results might require deployment of a functionally equivalent system in another participant with a similar level of paralysis and anarthria, which was not feasible here because we only have one active clinical-trial participant for this proof-of-concept study at the time of writing.
Randomization	Randomization was not critical to this single-participant study.
Blinding	Because the goal of the study was to demonstrate the feasibility of a spelling system controlled by a paralyzed person's silent-speech attempts, blinding was not relevant to this study. The study participant could see the sentence-spelling predictions in real time to emulate a real-world clinical use case.

Reporting for specific materials, systems and methods

We require information from authors about some types of materials, experimental systems and methods used in many studies. Here, indicate whether each material, system or method listed is relevant to your study. If you are not sure if a list item applies to your research, read the appropriate section before selecting a response.

Materials & experimental systems

n/a	Involved in the study
☒	☐ Antibodies
☒	☐ Eukaryotic cell lines
☒	☐ Palaeontology and archaeology
☒	☐ Animals and other organisms
☐	☒ Clinical data
☒	☐ Dual use research of concern

Methods

n/a	Involved in the study
☒	☐ ChIP-seq
☒	☐ Flow cytometry
☒	☐ MRI-based neuroimaging

Clinical data

Policy information about clinical studies

All manuscripts should comply with the ICMJEguidelines for publication of clinical research and a completedCONSORT checklist must be included with all submissions.

Clinical trial registration	Clinicaltrials.gov NCT03698149
Study protocol	A description of the study can be found at https://clinicaltrials.gov/ct2/show/NCT03698149. The full clinical-trial protocol can be found as a supplementary file associated with our previous publication with the same participant (https://www.nejm.org/doi/full/10.1056/NEJMoa2027540). The direct link to the study protocol is https://www.nejm.org/doi/suppl/10.1056/NEJMoa2027540/suppl_file/nejmoa2027540_protocol.pdf .
Data collection	Data collection occurred in the participant's bedroom or in a small office nearby. The clinical trial began in November 2018. The participant was recruited and enrolled in the study shortly afterwards, and he was implanted with the study device in February 2019 at UCSF Medical Center. The timeline of the proceeding data-collection sessions is provided in the supplementary materials with this manuscript. The data safety monitoring board agreed to the release of results in the manuscript prior to completion of the trial.<br><br>For the overall clinical trial, the currently approved duration of the clinical trial is 6 years, with the expectation that all 8 patients will be recruited in the first 4–5 years and data collection, device development, and analysis will be completed in 6 years. This information can be found in the attached clinical-trial protocol.
Outcomes	This clinical trial is a phase I single-center early feasibility study to evaluate the potential of ECoG-based neural interfaces for controlling advanced neuroprotheses that restore motor and communicative functions. Due to the exploratory nature of this trial and the limited number of trial participants, we did not pre-define specific secondary outcomes. Our primary endpoint which was pre-defined to assess the efficacy of the trial, and is stated in the protocol as "Feasibility of control of a wearable exoskeleton device and a communication interface." As such, a variety of analysis methods will be applied with trial participants throughout the trial with the aim to fully assess the efficacy of an ECoG-based neural interface for motor and communication restoration.
